# Activity-Based DNA-Encoded Library Screening for Selective Inhibitors of Eukaryotic Translation

**DOI:** 10.1021/acscentsci.4c01218

**Published:** 2024-10-04

**Authors:** Huda Barhoosh, Anjali Dixit, Wesley G. Cochrane, Valerie Cavett, Robin N. Prince, Brooke O. Blair, Fred R. Ward, Kim F. McClure, Phillip A. Patten, Margot G. Paulick, Brian M. Paegel

**Affiliations:** † Department of Pharmaceutical Sciences, University of California, Irvine, California 92697, United States; ¶ Initial Therapeutics, South San Francisco, California 94080, United States; § Departments of Chemistry & Biomedical Engineering, University of California, Irvine, California 92697, United States

## Abstract

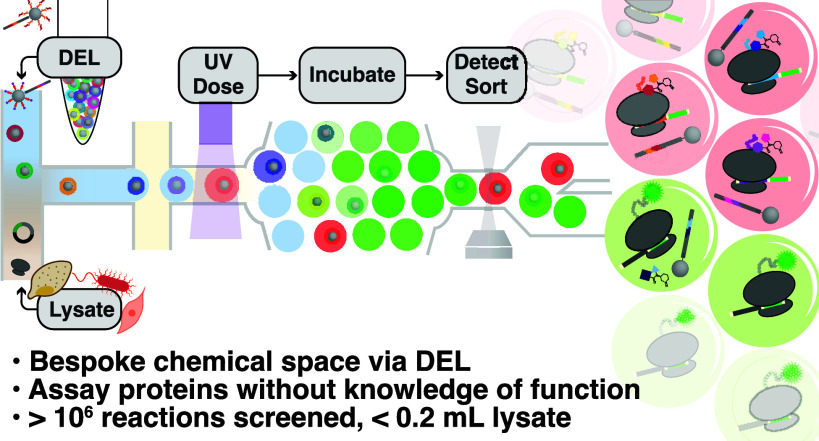

Small molecule probes exist for only ∼2% of human proteins because most lack functional binding pockets or cannot be assayed for high-throughput screening. Selective translation modulation circumvents canonical druggability and assay development constraints by using in vitro transcription–translation (IVTT) as a universal biochemical screening assay. We developed an IVTT activity assay by fusing a GFP reporter to various target gene sequences and screened the target sequences for inhibitors in microfluidic picoliter-scale droplets using a 5,348-member translation inhibitor DNA-encoded library (DEL). Screening a proof-of-concept PCSK9-GFP reporter yielded many hits; 6/7 hits inhibited PCSK9-GFP IVTT (IC_50_ 1–20 μM), and the lead hit reduced PCSK9 levels in HepG2 cells. Preliminary selectivity was informed by counterscreening the DEL against a frameshift mutant PCSK9-GFP reporter. A plug-and-play approach to assay development and screening was demonstrated by scouting the DEL for activity using reporter genes of targets with difficult-to-assay or even unknown function (RPL27, KRAS^G12D^, MST1, USO1). This microfluidic IVTT DEL screening platform could scale probe discovery to the human proteome and perhaps more broadly across the tree of life.

## Abstract 2

Microfluidic in vitro translation activity-based DNA-encoded library screening is a potentially scalable tool to discover small molecule probes across the human proteome and even perhaps the tree of life.

## Introduction

The human genome was originally sequenced to establish a genetic blueprint for mapping the proteome, understand the role of proteins in human disease, and prosecute those disease-related protein targets for drug discovery with high-throughput molecular screening (HTS).^1^ A network of NIH-funded HTS centers furnished ∼400 selective small molecule “probes” over a decade,^2^ but HTS does not scale to the ∼20,000+ genome-encoded proteins. The vast majority of proteins lack function that can be assayed by HTS or even suitable binding pockets. This “undruggable” proteome is rich in protein–protein interfaces (PPIs) and protein-nucleic acid interactions that are the foundation of much biological function.^3,4^

One such historically difficult-to-drug and high-value target is the cardiovascular disease biomarker proprotein convertase subtilisin/kexin type 9 (PCSK9). PCSK9 negatively regulates the low-density lipoprotein receptor (LDL-R) via a PPI with LDL-R,^5^ trafficking LDL-R to the lysosome for degradation.^6^ Consequently, current PCSK9-targeted therapeutics are not conventional small molecule therapeutics. Examples include monoclonal antibodies,^7,8^ the siRNA inclisiran,^9^ and oral cyclic peptide inhibitors of PCSK9.^10,11^ Pfizer conducted a cellular phenotypic high-throughput screen for small molecule inhibitors of PCSK9 and discovered (*R*)-*N*-(isoquinolin-1-yl)-3-(4-methoxyphenyl)-*N*-(piperidin-3-yl)propanamide (R-IMPP),^12^ a compound that decreases PCSK9 levels in cells. Mechanistic, structural, and animal studies revealed that the R-IMPP analogue PF-06446846 (PF846) binds adjacent the human ribosome exit tunnel, where it perturbs the PCSK9 nascent chain structure, inducing a conformation change in tRNA that selectively arrests PCSK9 mRNA translation and attenuates LDL-cholesterol (LDL-c) levels in vivo.^13−15^

Subsequent extensive medicinal chemistry efforts suggested a potentially general therapeutic modality via selective translation modulation. Fewer than 30 transcripts are sensitive to PF846, including the strongly stalled ribosomal protein L27 (RPL27), macrophage stimulating 1 (MST1), and others; subtle modifications of the PF846 structure can dramatically alter the sensitivity of different gene sequences.^14,16^ These observations hint at an opportunity to target novel ribosome nascent chains without requiring small molecule engagement. Reporter gene in vitro translation (IVT) was sufficient as a primary assay to evaluate both compound activity using the target PCSK9-luciferase transcript and compound selectivity using a panel of off target-luciferase fusions.^13^ Scaling this target-agnostic approach to the proteome would require IVT screening across a relatively narrow swath of chemical space as the structure–activity data around PF846 delineated fairly steep activity cliffs, particularly for the piperidine and tertiary amide moieties. Steep activity cliffs result in sparse structure–activity landscapes that are challenging for medicinal chemistry efforts, but DNA-encoded libraries (DELs) have recently proven useful in investigating such confounding RNA-based targets.^17^ Previously, DELs built in focused chemical spaces have also successfully identified high-potency benzamidine trypsin inhibitors,^18^ fragment pair inhibitors of PTP1B and TCPT,^19^ and probes of NAD^+^-binding pockets leading to the development of specific inhibitors of PARP15 and SIRT6.^20^

Here we describe a microfluidic activity-based DNA-encoded library (DEL) screening technology that grants access to a large collection of tertiary amides and picoliter-scale lysate in vitro transcription–translation (IVTT) assays to screen for selective inhibitors of translation. DELs, combinatorial small molecule collections in which each member is associated with a unique DNA barcode,^21−23^ were synthesized on microscopic beads for activity-based screening in droplets.^24−26^ The 5,348-member DEL was screened using a microfluidic droplet-scale assay for inhibitors of eukaryotic IVTT. PCSK9 was used as a proof-of-concept target nascent chain with its frameshift mutant as a counter screening target. Hits from these first screens, which featured novel tertiary amide structures, selectively inhibited PCSK9 translation in vitro and reduced PCSK9 protein levels in HepG2 cells. The assay was easily adapted to screen multiple target nascent chains, demonstrating a “plug and play” approach that does not require prior knowledge of target function and a scalable path forward to probing the proteome.

## Results and Discussion

### Adaptation of Plant Cell-Free Translation as an Activity Assay for Screening

**Figure 1:**
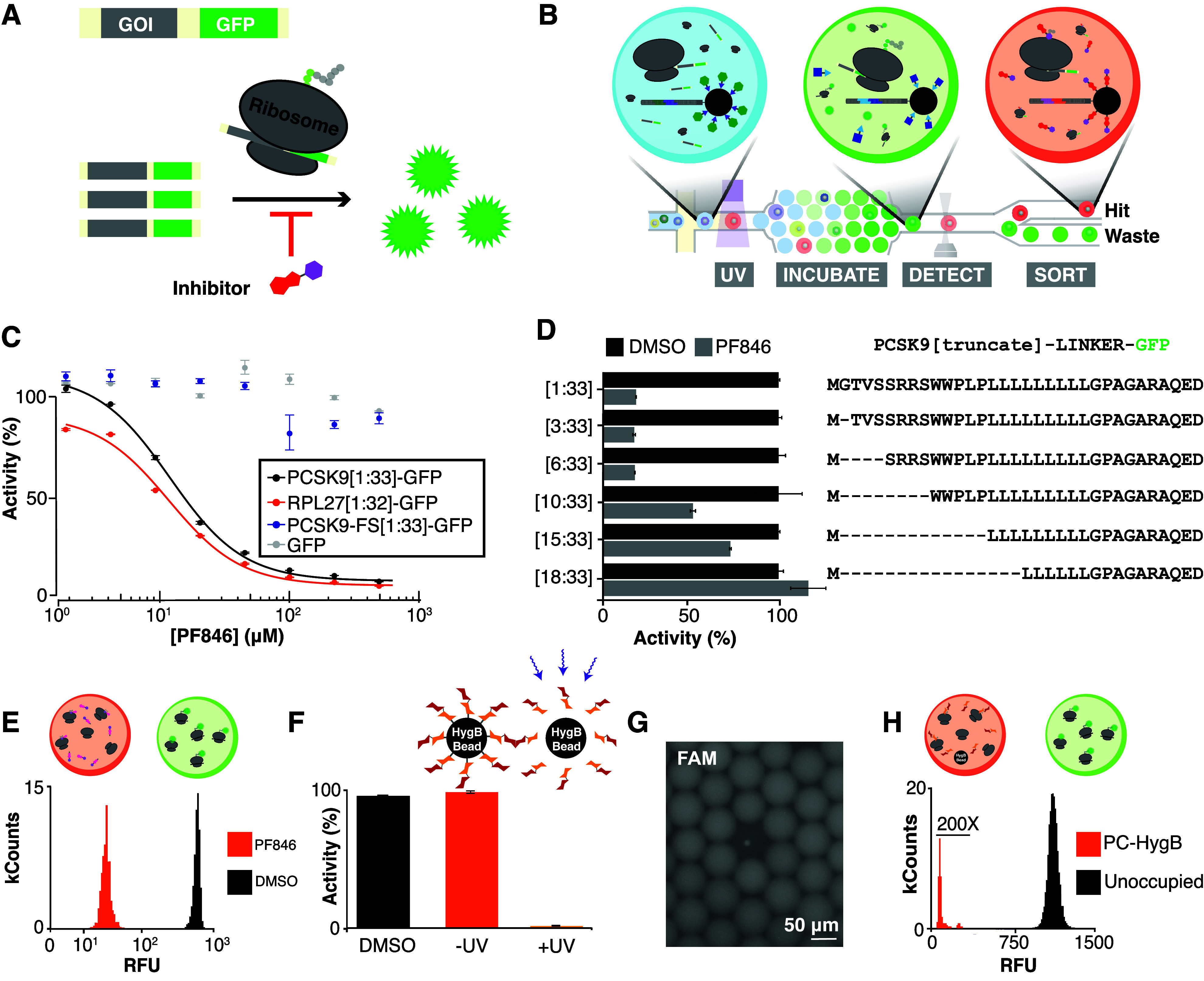
WG IVTT activity assay development and miniaturization. (A) DNA encoding the target gene of interest (GOI) is fused to a GFP reporter. A translation inhibitor decreases GFP fluorescence. (B) Microfluidic activity-based DEL screening entails library bead encapsulation in IVTT droplets, DEL member photocleavage from the bead, droplet incubation, and droplet sorting based on GFP fluorescence. High-fluorescence droplets contain inactive compounds or are empty (green) and default to waste while low-fluorescence hit droplets (red) contain active compounds and are sorted. (C) PF846 dose–response IVTT activity assays of PCSK9[1:33]-GFP, PCSK9-FS[1:33]-GFP, RPL27[1:32]-GFP, and the GFP reporters and (D) end point assays (100 μM PF846) of PCSK9[1:33]-GFP N-terminal deletions were conducted to determine sensitivity to PF846 in WG IVTT (% activity relative to DMSO control). (E) Microfluidic droplets of PCSK9[1:33]-GFP IVTT reaction and DMSO control (black) or 100 μM PF846 (orange) were generated and analyzed to calculate assay *Z*′ = 0.7. (F) PC-HygB beads were photocleaved, and the photocleavage supernatant was used in a PCSK9[1:33]-GFP WG IVTT assay. (G) Microfluidic droplets containing PC-HygB beads were imaged using epifluorescence. (H) PC-HygB positive control translation inhibitor beads were loaded in droplets of PCSK9[1:33]-GFP WG IVTT. HygB-beaded droplets (orange) and unoccupied droplets (black) were used to calculate *Z*′ = 0.75 (bead-occupied population magnified 200×).

A commercially available wheat germ lysate (WG) IVTT system was employed to assay translation inhibition. Translation activity was detected via a GFP reporter, and the assay was miniaturized to picoliter-scale microfluidic droplets (Figure 1A,B). To validate the assay, ribosome nascent chains and mutants previously known to exhibit various sensitivities to PF846 were constructed and evaluated in WG IVTT.^13^ PCSK9 and RPL27 were truncated to the first 33 and 32 amino acids, respectively (PCSK9[1:33] and RPL27[1:32]). The sequences contain distinct PF846 stall sites and were both fused to a GFP reporter. The GFP reporter and a frameshift mutant of PCSK9[1:33] (PCSK9-FS[1:33]), which mutates the nascent chain sequence while maintaining a nearly identical mRNA sequence, were included as negative controls.^13^ PF846 treatment inhibited the translation of PCSK9[1:33]-GFP and RPL27[1:32]-GFP but did not affect the translation of controls (Figure 1C). Sequence sensitivity was further confirmed by an N-terminal deletion series of PCSK9[1:33]-GFP; deletion of N-terminal residues increasingly ablated PCSK9[1:33]-GFP translation sensitivity to PF846 (Figure 1D). The WG IVTT assay was miniaturized using a previously described microfluidic circuit modified for longer incubation times (Figure S1)^27^ using PF846 as a positive control compound, and assay statistical quality was calculated (Z′=0.7) (Figure 1E). To confirm detection of translation inhibition from library beads, hygromycin B, a nonselective aminoglycoside translation inhibitor, was coupled to beads displaying a photocleavable linker (PC-HygB). UV irradiation releases hygromycin from the bead, allowing it to interact with the WG IVTT assay in the droplet. PC-HygB bead photocleavage supernatant inhibited PCSK9[1:33]-GFP translation both in a microplate assay and in microfluidic droplets (Figure 1F,G, S2). Assay statistical quality using PC-HygB positive control beads was similarly calculated (Z′ = 0.75, Figure 1H).

The WG IVTT assay performance was excellent in microplates and microfluidic droplets, recapitulating known PF846 activity on human ribosomes using a plant-based system. An assay Z^′^ > 0.5 gives sufficient separation between negative and positive control to conduct a screen;^28^ the assay quality here (Z^′^ = 0.7 for PF846 in PCSK9[1:33]-GFP IVTT droplets, Z^′^ = 0.75 for PC-HygB beads in PCSK9[1:33]-GFP IVTT droplets) is on par with or superior to other biochemical assays that we have miniaturized from microplate to picoliter-scale droplets (Z^′^ = 0.6–0.9).^25,29^ Prior translation inhibition studies used HeLa lysate with a luciferase (Luc) reporter, but the WG IVTT system was conducive to microfluidic screening. The PCSK9-GFP reporter nascent chain sequence sensitivity to PF846 using plant ribosomes is consistent with known activity in PCSK9 translation reactions using other eukaryotic ribosomes (human, rabbit, plant, fungal), and even more granular gene structure–activity is conserved. Alteration of the gene sequence through systematic deletions of the N terminus results in almost identical sensitivity to PF846 between plant and human ribosomes: short deletions of the N terminus preserve PF846 activity, but deletion of 15 or more residues completely eliminates PF846 activity.^13^ Nonselective inhibitors of ribosomal protein synthesis such as HygB are also agnostic of ribosome source, and thus HygB is an ideal positive control for screening in anticipation of pursuing gene targets that are insensitive to PF846.

### DEL Synthesis Furnishes a Large Library of Translation Inhibitor Analogues for Screening

**Figure 2:**
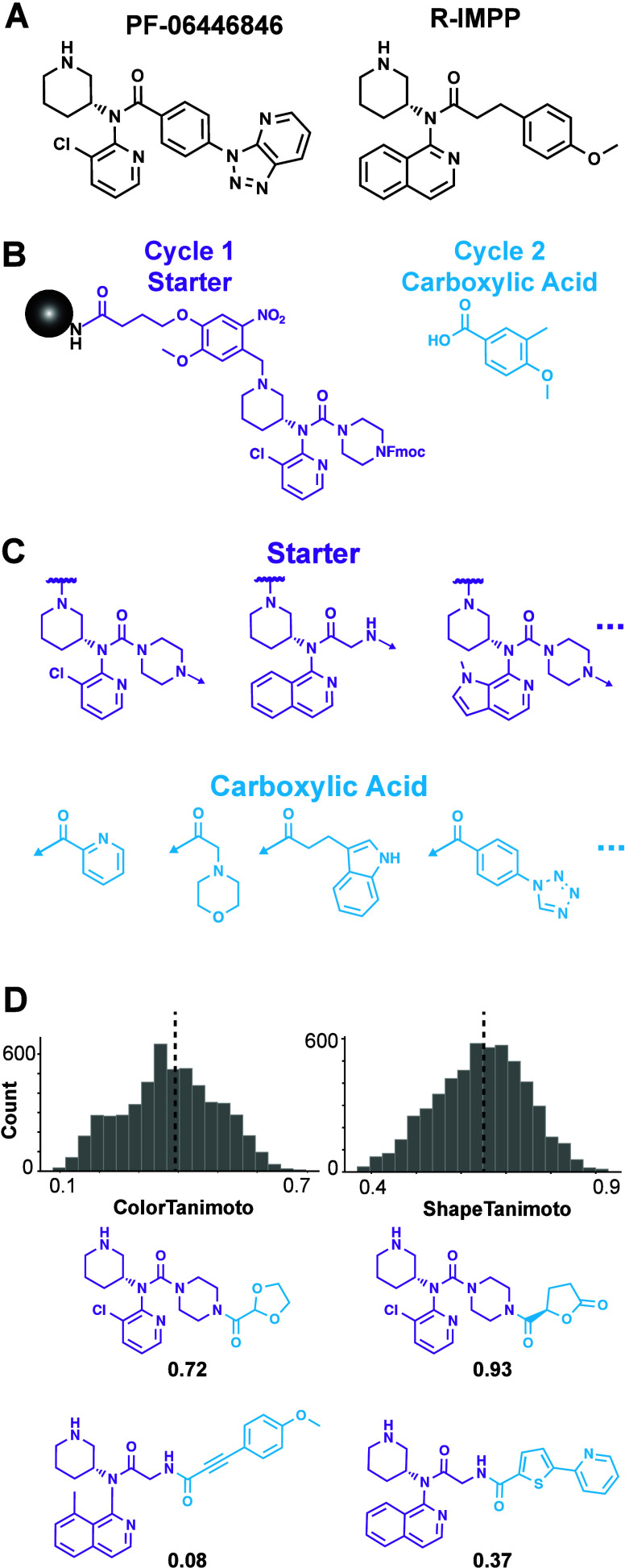
Bespoke translation inhibitor DEL design. (A) PF846 and R-IMPP inhibit PCSK9 translation. (B) Fmoc-protected custom “starter” BBs (14, purple) were synthesized on a photocleavable linker. Fmoc was removed and starters were acylated with carboxylic acids (382, cyan), yielding 5,348 DEL members. (C) Example starters and acids are shown. Starters preserved the piperidine but contained various N-substituted tertiary amides and an Fmoc-amine for carboxylic acid elaboration (3 example starters shown). The set of carboxylic acids was structurally diverse (4 example acids shown). Arrows indicate the location of coupling between cycle 1 and cycle 2 BBs. (D) 3D color and shape Tanimoto similarities of the library members were calculated referencing energy-minimized PF846. Example library members with the highest and lowest scores are depicted.

A bespoke DEL was designed and synthesized to explore the chemical space around known selective translation inhibitors. PF846 and R-IMPP^12,13^ share an amino piperidine/heterocyclic N-substituted tertiary amide ”headgroup” responsible for binding to rRNA in the 80S exit tunnel^15^ and vary in their ”tail” (Figure 2A). Head group building blocks (BBs) (14) were prepared as photocleavable Fmoc-protected cycle 1 “starters” for DEL synthesis, and carboxylic acid tail BBs (382) were coupled in cycle 2 (Figure 2B). Head groups explored various N-substituted aromatic heterocycles,^16^ but all retained the amino piperidine moiety. The carboxylic acid BB set contained diverse aromatic and aliphatic features and was filtered by molecular weight (<200 Da), structural flags,^30^ and stereochemistry (Figure 2C). DEL members (5,348) ranged in 3D Tanimoto color (0.1–0.7; median = 0.38) and shape similarity (0.4–0.9; median = 0.65) using energy-minimized PF846 as the reference (Figure 2D). The DEL was synthesized and validated (Figures S3–S6) according to previously published protocols.^24^

Previous SAR and cryo-EM structural analyses of PF846 binding highlighted key interactions with the translating ribosome, and the library was designed with these interactions in mind. PF846 engages with rRNA residues through specific interactions with the amino piperidine and chloropyridine.^15^ All starter BBs therefore preserved positioning of the amino piperidine, but varied the amide N-aryl group.^16^ The triazolopyridine tail of PF846 projects into the exit tunnel to interact with and attenuate the translation of select nascent chains, and therefore cycle 2 BBs included a set of acids to address diverse target nascent chains. We were not structurally enabled due to the dynamic nature of the nascent chain, so we chose tails with drug-like properties. Fully enumerated compounds lie in near-normal distributions of Tanimoto shape and color similarity to PF846, providing a sampling of chemical space neither extremely similar to nor extremely different from PF846 and R-IMPP.

### DEL Activity Is Highly Gene-Specific, with High Hit Rate for PCSK9 Compared to Its Frameshift Mutant

**Figure 3:**
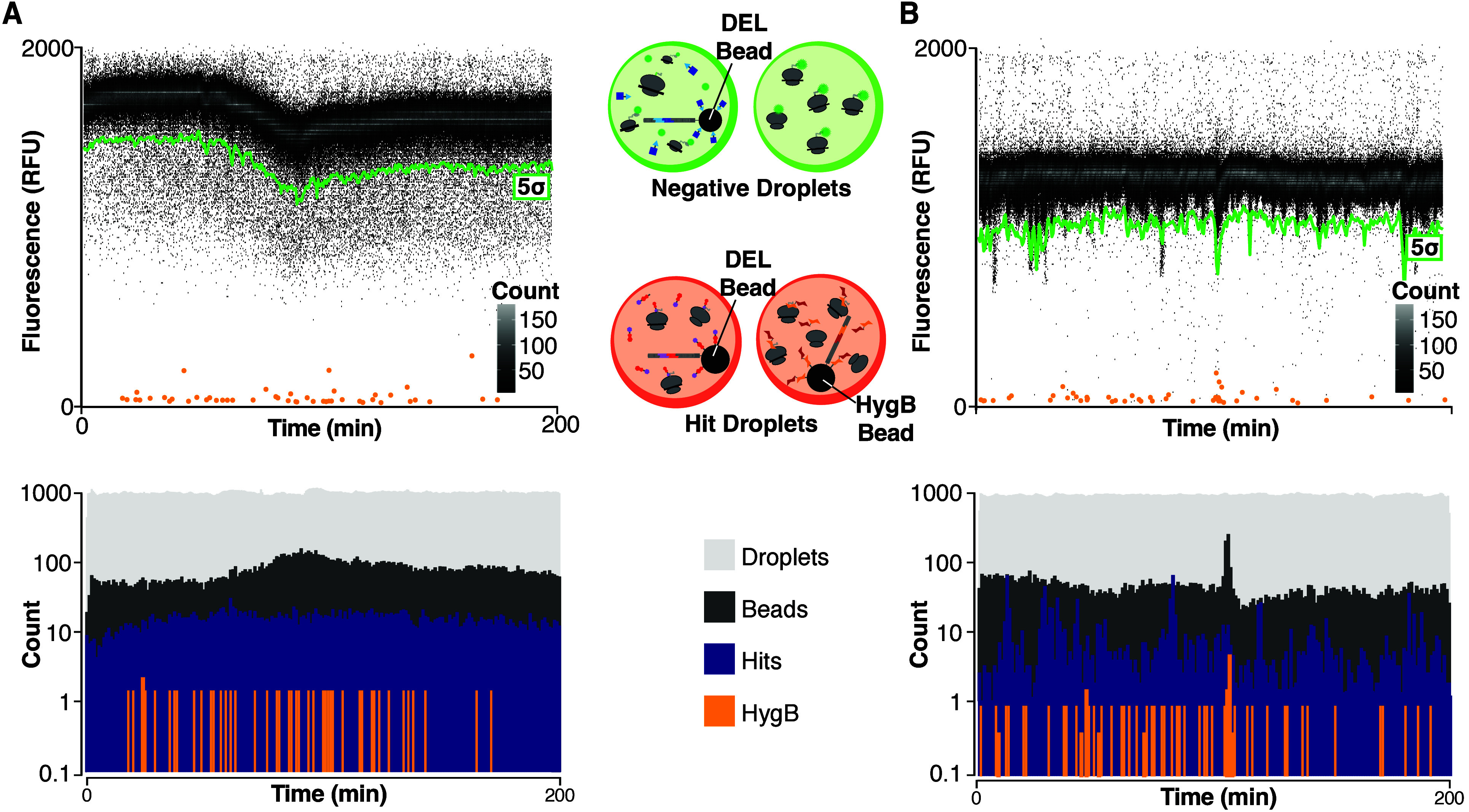
Microfluidic activity-based DEL screens for inhibitors of PCSK9[1:33]-GFP and PCSK9-FS[1:33]-GFP WG IVTT. (A) PCSK9[1:33]-GFP or (B) PCSK9-FS[1:33]-GFP IVTT droplets were combined with DEL beads and PC-HygB control beads. Transient histogram visualization of the screen shows population of negative droplets, which determine the dynamic sorting threshold (green, 5σ below the mean), and hit droplets containing active DEL or HygB control beads (orange). Droplet data were binned by time and fluorescence (30 s, 7 RFU, top). Droplet generation, bead introduction, and library and control bead hit rate were consistent throughout the screen (bottom).

The validated PF846-inspired library was screened to discover novel translation inhibitors. DEL beads were encapsulated in microfluidic droplets of WG IVTT and irradiated with UV in flow to liberate DEL members into the droplets. The photodosed droplets were incubated and sorted based on fluorescence.^25^ PCSK9[1:33]-GFP was chosen as a proof-of-concept target, and PCSK9-FS[1:33]-GFP was used as a counter screen target to identify nonselective compounds. PC-HygB were mixed with DEL beads and screened in the microfluidic device for inhibitors of translation. Beads that statistically significantly suppressed droplet IVTT fluorescence (5 standard deviations, 5σ) were isolated, screening 90,000 beads in ∼1.5MM droplets using just ∼200 μL of IVTT. PCSK9[1:33]-GFP and PCSK9-FS[1:33]-GFP exhibited hit rates of 17% and 3%, respectively (Figure 3, top). Droplet generation, bead introduction, and library and control bead hit rate were uniform during the screen (Figure 3, bottom).

The microfluidic activity-based DEL screen performance is robust, generating stable translation activity signal that reliably identifies highly active positive control beads and prospective hit DEL beads. The consistent assay signal enables dynamic statistical hypothesis testing and thereby rigorous identification and sorting of hit droplets with statistically significantly attenuated fluorescence intensity. Putative PC-HygB-containing droplets were observed at regular cadence throughout the screen, increasing confidence in screening performance irrespective of library hit rate. The dramatic difference in hit rate between PCSK9[1:33]-GFP and PCSK9-FS[1:33]-GFP screens implies the presence of DEL members that selectively inhibit PCSK9[1:33]-GFP translation at the PF846 stall site of PCSK9.

### DEL Screen Deconvolution Identifies Strong Hit Families That Validate in IVTT and Cellular Assays

**Figure 4:**
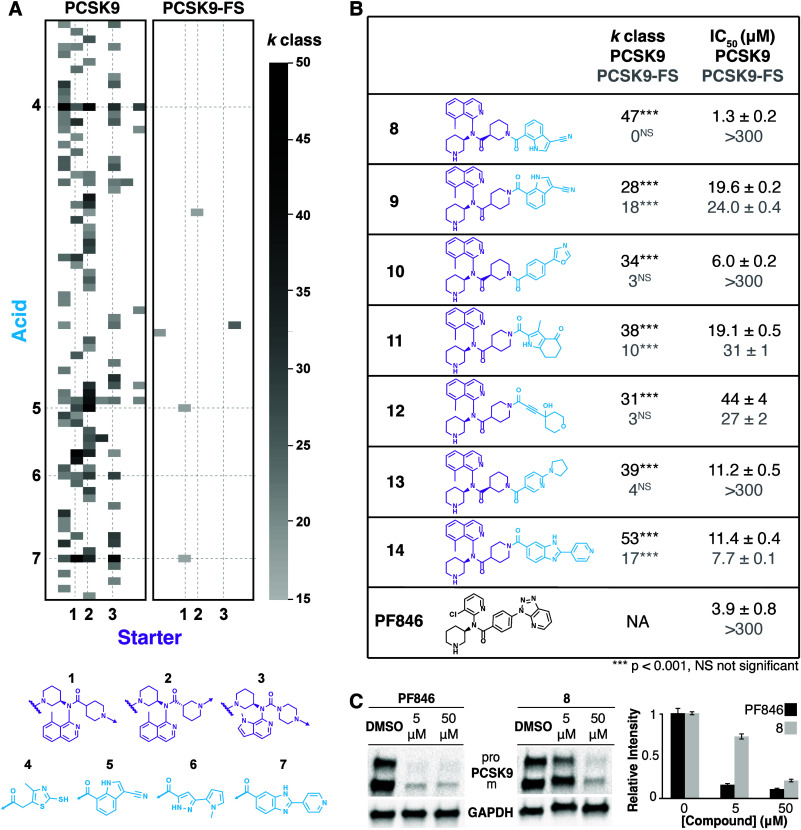
DEL screening hit *k* class analysis and validation. (A) Hits from the DEL screens of reporters PCSK9[1:33]-GFP and PCSK9-FS[1:33]-GFP were visualized as a heat map by cycle 1 starter and cycle 2 acid (replicate *k* class indicated in grayscale). Starters **1**–**3** and acids **4**–**7** were statistically overrepresented in the hit collection (*p* < 0.0001). Arrows indicate the location of coupling between cycle 1 (purple) and cycle 2 (cyan) BBs. (B) Hit structures **8**–**14** were synthesized and validated in WG IVTT assays in triplicate; *k* class (listed by gene target) and WG IVTT IC_50_ values are summarized. Statistical significance of *k* class values is summarized as *p* < 0.001 (***) or not significant (^NS^). Undetectable IC_50_ values are marked >300. (C) PCSK9 expression in HepG2 cells exposed for 16 h to PF846 or **8** was measured in triplicate and analyzed by Western blot. Gel band quantitation normalized to vehicle control (DMSO) is shown at right. All uncertainties are standard deviation of the mean.

Hit collection DNA tags from the PCSK9[1:33]-GFP and PCSK9-FS[1:33]-GFP screens were sequenced to deduce the structures of molecules that inhibited PCSK9[1:33]- or PCSK9-FS[1:33]-GFP translation. Hit structure *k* class, the number of times a given hit structure is observed on distinct beads,^31^ was summed across screens and visualized as a heat map (Figure 4A). Random samples of the DEL approximating the average hit bead collection size (3 × 1500-bead samples) were also sequenced to establish a false discovery matrix; hits of *k* ≥ 3 had a false discovery rate <2% (Figure S7). The combined hit sets for the two templates contained 121 unique hits with *k* ≥ 17 across the two screening campaigns. The most frequently observed cycle 1 starter BBs were **1** and **2**, which share an 8-methylisoquinoline moiety, but differ in the vector for cycle 2 carboxylic acid connection, and **3**, which contains an *N*-methylazaindole moiety. The most frequently identified cycle 2 tails included carboxylic acids **4**–**7**. Acid **4**, a mercapto-methylthiazole, was observed with 6 starters, and 3-cyano-indole acid **5** appeared with starters **1** and **3**. Substituted pyrazole **6** and pyridyl-benzimidazole **7** also appeared frequently with the aforementioned starters. There were 5 hits that appeared with high *k* class in both PCSK9[1:33]-GFP and PCSK9[1:33]-FS-GFP screening campaigns.

Several hit compounds were synthesized and assayed for activity in WG IVTT using both PCSK9[1:33]-GFP and PCSK9-FS[1:33]-GFP templates. IC_50_ and dose–response profiles varied for hit compounds (Figures 4B and S8). Compound **8** moderately inhibited PCSK9[1:33]-GFP translation, with an IC_50_ similar to that of PF846, and was inactive on PCSK9-FS[1:33]-GFP translation. Compounds **10** and **13** were observed at a higher *k* class in PCSK9[1:33]-GFP screens when compared to PCSK9-FS[1:33]-GFP screens. They modestly inhibited PCSK9[1:33]-GFP translation with minimal activity on PCSK9-FS[1:33]-GFP. Hits **9**, **11**, **12**, and **14** were similarly active on both templates with all but one having high *k* class in both the primary and counter screens. Compound **8**, the most potent hit, and its regioisomer **9** were further investigated in a HeLa-derived IVTT assay; both compounds were active in human IVTT (Figure S9). When translation was initiated from mRNA rather than plasmid DNA, compound **8** was active while compound **9** was inactive. PCSK9[1:33]-GFP in vitro transcription reactions treated with **9** showed no visible transcript product (Figure S10). In HepG2 cells, overnight treatment with PF846 or **8** significantly reduced PCSK9 protein levels (Figures 4C and S12).

Hits generally validated, and target selectivity correlated with screening data. The *k*-class-dependent false discovery rate (FDR) established statistical thresholds that informed decisions to commit further synthesis resources for hit follow up.^31^ In an analysis of random samples of the DEL (3 × 1500-bead), ∼2% of all called hit structures are *k* ≥ 3, constituting an acceptable FDR (<5%) for synthesis. Hit selection was also prioritized according to implied SAR from DEL screening data. For example, BBs that frequently appeared in the PCSK9[1:33]-GFP screening campaign but not in PCSK9-FS[1:33]-GFP should exhibit template-specific inhibition. Synthesized hits displayed varying selectivity profiles that were consistent with the primary screening data. Abundantly observed compound **8** was not detected in any PCSK9-FS[1:33]-GFP screens and was found to inhibit PCSK9[1:33]-GFP translation but not PCSK9[1:33]-FS translation. The *k* class also informed hit synthesis of **10** and **13**, which had higher *k* class for PCSK9[1:33]-GFP compared to PCSK9-FS[1:33]-GFP. Hit **9**, a regioisomer of **8**, was selected as we anticipated regiochemistry would profoundly alter activity, not necessarily selectivity, as structure–activity studies uncovered steep cliffs in the geometry of the linker connecting the tertiary amide to the triazolopyridine tail.^16^ That **9** universally and completely inhibited the translation of all reporters prompted investigation of alternative mechanisms of action. Compound **9** is inactive when IVTT is initiated with RNA, and it completely eliminates RNA yield from in vitro transcription reactions, collectively suggesting that 9 may be an RNA polymerase inhibitor. Such compounds that inhibit IVTT indiscriminately can be identified by counter screens, and hits that are significantly enriched in both primary and counter screens can be deprioritized for hit synthesis. Compound **8**’s selectivity profile diverges from PF846 in that it is inactive on MST1, one of the strongest PF846 off targets (Figure S11), demonstrating that DEL screening can identify translation inhibitors with altered target selectivity. Activity in HeLa and WG IVTT tracked, in agreement with both our study of PCSK9[1:33]-GFP truncates and prior results demonstrating conservation of activity across a spectrum of eukaryotic ribosome sources.^13^ Compound **8**’s inhibition of PCSK9[1:33]-GFP IVTT reactions reproduced in a cellular assay, where HepG2 cells treated with **8** produced lower levels of endogenous PCSK9. Compound potency was lower in the cellular assay, likely owing to differences in cellular membrane permeation or other common pharmacokinetic parameters that are not relevant in vitro but become critical in cellular or animal studies.

### Microfluidic IVTT Screening Obviates Gene-Specific Assay Development

**Figure 5:**
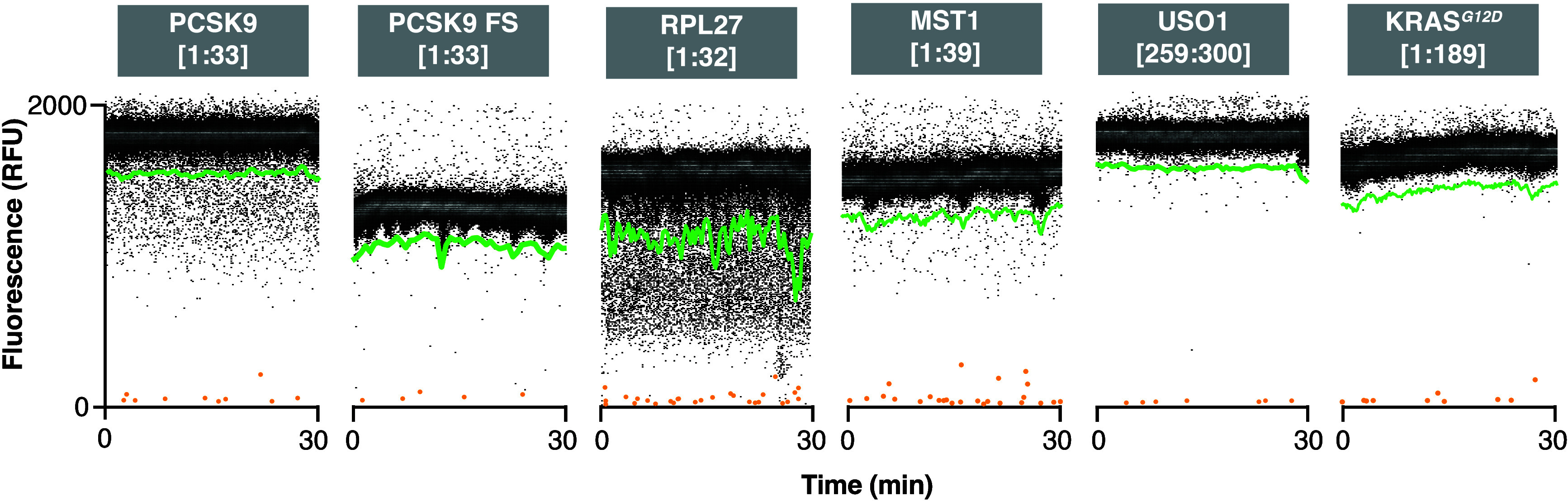
Target protein function-agnostic scouting. Transient histogram visualization of microfluidic in vitro translation activity-based DEL screens of PCSK9[1:33]-GFP, PCSK9-FS[1:33]-GFP, RPL27[1:32]-GFP, MST1[1:39]-GFP, USO1[259:300]-GFP, and KRAS G12D[1:189]-GFP shows the population of negative droplets, which determine the dynamic sorting threshold (green, 5σ below the mean), and hit droplets containing active DEL or HygB control beads (orange). Droplet data were binned by time and fluorescence (30 s, 7 RFU).

To test the feasibility of target-agnostic screening, several nascent chains were analyzed in ”scouting” mode using the same DEL. Known targets of PF846, RPL27[1:32]-GFP, MST1[1:39]-GFP, and USO1[259:300]-GFP were screened along with KRAS G12D[1:189]-GFP against the DEL. Hit rate varied with the templates screened (Figure 5). RPL27[1:32]-GFP had the highest hit rate followed by PCSK9[1:33]-GFP. MST1[1:39]-GFP and USO1[259:300]-GFP exhibited moderate and low hit rates, respectively. PCSK9-FS[1:33]-GFP and KRAS G12D[1:189]-GFP had a minimal number of hits. PC-HygB control beads were observed in all screens.

The platform was successfully adapted to screen different targets without requiring additional assay development for each gene. This is an important feature as assay development for new targets can require substantial resources and may not be possible or feasible if the target’s function is unknown. As an example, KRAS^G12D^, an important and difficult oncology target, was successfully translated in droplets as the full-length protein without compromising the assay dynamic range. Screening full-length constructs is advantageous when scouting new target genes that have unknown windows of compound sensitivity, unlike PCSK9[1:33]. Hit rate varied between target genes, suggesting that opportunities exist for discovering scaffolds with novel gene selectivity such as PCSK9 screening hit **8**, which exhibited such altered selectivity.

Discovering hits with altered target gene selectivity is a major goal and requirement for translation stalling to be therapeutically applied across the proteome. Structure–activity analysis of the DEL screens reveals selectivity trends that could seed new lead optimization campaigns. For example, the orientation of the nitrile tail in regioisomers **8** and **9** confers very different activity; **8** is an inhibitor of PCSK9[1:33]-GFP translation, while **9** nonselectively inhibits transcription. Compound **8** additionally differs from PF846’s chloropyridine headgroup and phenyl linker. While these structural changes maintain activity for PCSK9[1:33]-GFP with similar potency as PF846, off-target activity varies between the two compounds. Compound **8** is inactive against MST1[1:39]-GFP and most active against RPL27[1:32]-GFP. This is in contrast to PF846, which more potently inhibits MST1[1:39]-GFP translation than PCSK9[1:33]-GFP and RPL27[1:32]-GFP translation.^13^ The tail of compound **8** may interact with the peptide nascent chains differently than PF846. These subtle changes in selectivity could impact the toxicity profile for **8**, specifically in the context of hematopoietic toxicity.^16^ Since MST1 plays an integral role in hematopoietic stem cell regulation,^32,33^**8**’s lack of activity on MST1 translation may result in an improved safety profile. Although the in vitro and cellular data support the hypothesis that **8** exhibits comparable selective translation modulation activity to PF846, a full analysis of off targets via ribosome profiling is necessary to substantiate **8** as a more promising point to initiate further structure–activity studies.^13^

We are further emboldened to design, synthesize, and screen more structurally diverse DELs in an effort to depart from the tertiary amide scaffold used here. This proposal has not previously been warranted or even feasible as the initial discovery of R-IMPP resulted from a 2.5-million-compound screen wherein only one hit exhibited the desired activity. Parallel medicinal chemistry efforts were also understandably conservative as the tertiary amide scaffold presented synthetic challenges and, until recently, structural data did not exist. Complicating matters further, PF846 and its analogues^14,34^ engage with the rRNA through the chloropyridine and piperidine moieties, and the triazolopyridine tail group interacts in an as yet unknown fashion with the peptide nascent chain in the ribosome exit tunnel. This interaction drives a gross misalignment of the tRNA at the ribosome peptidyl transferase catalytic center and concomitant translation stalling.^15^ DEL synthesis and microfluidic library screening dramatically lower the stakes of exploring chemical space more liberally.

The microfluidic IVTT screening platform generally expands the scalability of drug discovery from multiple perspectives. DEL synthesis grants access to appropriate and highly customized chemical matter in a ”consumable” format. Furthermore, the combination of modular in vitro translation assay programming and microfluidic miniaturization renders both assay development and screening scalable. This combination has been highly potent in realizing numerous cell-free expression-driven platforms for mutational landscape analysis, genetic circuit optimization, and pioneering studies in directed evolution.^35−39^ Translation modulation as a prospective modality leverages analogous advantages in terms of library synthesis and assay generality, but now in the context of drug discovery. The pilot DEL synthesis yielded ∼100k equivalents of library that would have been sufficient to screen ∼5,000 target genes, with each target requiring 500 μL lysate. Importantly, no *a priori* knowledge of target function is necessary as target gene sequences are simply fused to the reporter construct using standard synthetic biology tactics.

Screening for translation modulators is poised to become a general probe discovery tool with utility in other human disease therapy areas and even across the tree of life.^40^ Homoharringtonine, for example, is a clinical translation inhibitor used in the treatment of chronic myeloid leukemia which functions by preventing initial elongation of protein synthesis globally.^41^ The rocaglates, which target eIF4A initiating translation on purine-rich transcripts, are more sequence-selective than homoharringtonine.^42^ Substantial optimization of the rocaglamide A scaffold yielded zotatifin,^43^ which exhibits promising activity in triple-negative breast cancer and is in ongoing clinical trials. Prokaryotic translation inhibition is already a well-known antibacterial drug mechanism of action,^44^ thus screening bacterial IVTT against appropriate DELs may identify novel molecular scaffolds that are simpler than the macrolides or bacterial gene sequence targets that are particularly prone to small molecule-mediated stalling.^45−47^ Extending this approach to viral translation is also potentially fruitful. SARS-CoV-2, for example, is particularly susceptible to translation modulation.^48^ Finally, modulating plant translation is an emerging strategy for the development of herbicides,^49^ and altering key gene expression levels dramatically increases crop yield.^50^ As we explore these and other branches of the phylogenetic tree, we will learn the extent to which small molecule modulation of ribosomal protein synthesis is conserved, and whether proteome-wide drug discovery via this platform will pay dividends beyond the confines of human biology.

## Supporting Information Available

Materials, experimental methods, synthetic routes, library and structural characterization, biological activity assay data (PDF)
