# Supplementary material for: Activity-Based DNA-Encoded Library Screening for Selective Inhibitors of Eukaryotic Translation
Source: ACS Cent Sci. 2024 Oct 4;10(10):1960–8. doi: 10.1021/acscentsci.4c01218 (PMC11503492; doi:10.1021/acscentsci.4c01218)
Supplement: Supplementary file 1 — oc4c01218_si_001.pdf [file oc4c01218_si_001.pdf]

Supporting Information: Activity-Based DNA-Encoded Library Screening for Selective Inhibitors of Eukaryotic Translation

Huda Barhoosh,[†,‡] Anjali Dixit,[†,‡] Wesley G. Cochrane,[†] Valerie Cavett,[†]
1 Robin N. Prince,[¶] Brooke O. Blair,[¶] Fred R. Ward,[¶] Kim F. McClure,[¶]
Phillip A. Patten,[¶] Margot G. Paulick,[*,¶] and Brian M. Paegel[*,†,§]

†*Department of Pharmaceutical Sciences, University of California, Irvine*

‡*H.B. and A.D. contributed equally to this paper*

¶*Initial Therapeutics, South San Francisco, CA*

§*Departments of Chemistry & Biomedical Engineering, University of California, Irvine*

E-mail: mpaulick@initialtx.com; bpaegel@uci.edu

Contents

Materials **S-5**

Buffers **S-6**

Methods **S-7**

Bifunctional Resin Synthesis and Characterization S-7

Azido Headpiece DNA Synthesis, Purification, and Characterization S-8

Encoded Library Synthesis S-9

DNA-encoded library resin barcoding S-9

Cycle 1 Chemistry S-9

HDNA Click and Characterization S-10

Opening Sequence (OS) Ligation S-11

Cycle 1 Encoding S-11

Cycle 2 (24xx, 15xx) Encoding S-12

Cycle 2 Chemistry S-13

Bead-Specific Barcoding (26xx) S-13

Library Quality Control S-13

HygB Control Bead Synthesis S-15

Microfluidic Device Fabrication S-16

Microfluidic Device Operation and Acquisition S-16

Droplet Incubation Analysis S-18

Droplet-Scale Flow Injection Analysis of WG IVTT Assay Quality S-18

Library Screening in Droplets S-18

Hit Bead Preparation for Illumina High-Throughout Sequencing S-19

NGS Decoding S-20

Microplate IVTT Assays S-21

Target Genes S-22

HepG2 Cellular Assay and Western Blot S-22
Coding Oligonucleotides S-24
Compound Synthesis and Characterization S-25
Compound **1** Synthesis S-25
Compound **2** Synthesis S-29
Compound **3** Synthesis S-32
Synthesis of Tert-butyl (R)-3-(N-(8-methylisoquinolin-1-yl)piperidine-4-carboxamido) piperidine-1-carboxylate (Intermediate 1) S-36
Synthesis of Compound **9** S-37
Synthesis of Compound **11** S-39
Synthesis of Compound **12** S-41
Synthesis of Compound **14** S-43
Synthesis of Benzyl (S)-3-(((R)-1-(tert-butoxycarbonyl)piperidin-3-yl)(8-methylisoquinolin-1-yl)carbamoyl)piperidine-1-carboxylate (Intermediate 2) S-44
Synthesis of Compound **8** S-46
Synthesis of (S)-N-(8-methylisoquinolin-1-yl)-1-(4-(oxazol-5-yl)benzoyl)-N-((R)-piperidin-3-yl)piperidine-3- carboxamide (Compound **10**) S-47
Synthesis of Compound **13** S-49

Supporting Figures **S-51**
Supporting Figure S1 . . . . S-51
Supporting Figure S2 . . . . S-52
Supporting Figure S3 . . . . S-53
Supporting Figure S4 . . . . S-54
Supporting Figure S5 . . . . S-55
Supporting Figure S6 . . . . S-56
Supporting Figure S7 . . . . S-57
Supporting Figure S8 . . . . S-58

Supporting Figure S9 . . . . S-59
Supporting Figure S10 . . . . S-60
Supporting Figure S11 . . . . S-61
Supporting Figure S12 . . . . S-62

Supporting Tables **S-63**
High-Resolution Mass Spectra S-63
Vector S-64
Target Gene Sequences S-65

References **S-67**

Materials

Material	Abbreviation	Source
N,N-Dimethylformamide	DMF	ThermoFisher Scientific (Waltham, MA)
N,N-Dimethylacetamide	DMA	ThermoFisher Scientific (Waltham, MA)
Dichloromethane	DCM	ThermoFisher Scientific (Waltham, MA)
N,N'-Diisopropylcarbodiimide	DIC	ThermoFisher Scientific (Waltham, MA)
N,N-Diisopropylethylamine	DIEA	ThermoFisher Scientific (Waltham, MA)
Acetonitrile	ACN	ThermoFisher Scientific (Waltham, MA)
Water (HPLC Grade)	H2O	ThermoFisher Scientific (Waltham, MA)
Scintillation vials		ThermoFisher Scientific (Waltham, MA)
Adhesive PCR plate foils		ThermoFisher Scientific (Waltham, MA)
10,000X SYBR Gold		ThermoFisher Scientific (Waltham, MA)
Magnesium chloride hexahydrate	$MgCl_2$	ThermoFisher Scientific (Waltham, MA)
N-Hydroxysuccinimide	NHS	ThermoFisher Scientific (Waltham, MA)
Sodium acetate anhydrous	NaOAc	ThermoFisher Scientific (Waltham, MA)
Linear polyacrylamide		ThermoFisher Scientific (Waltham, MA)
Ascorbic acid		ThermoFisher Scientific (Waltham, MA)
Adhesive PCR Plate Foils		ThermoFisher Scientific (Waltham, MA)
N-α-Fmoc-N-ε-7-methoxycoumarin-4-acetyl-L-lysine	Fmoc-Lys(mca)-OH	Sigma Aldrich (St. Louis, MO)
4-Methylpiperidine	4-MePip	Sigma Aldrich (St. Louis, MO)
Acetic anhydride	AcOAc	Sigma Aldrich (St. Louis, MO)
Trifluoroacetic acid	TFA	Sigma Aldrich (St. Louis, MO)
Triisopropylsilane	TIPS	Sigma Aldrich (St. Louis, MO)
α-Cyano-4-hydroxycinnamic acid	HCCA	Sigma Aldrich (St. Louis, MO)
Molecular sieves (3 Å)		Sigma Aldrich (St. Louis, MO)
1,3-Bis[tris(hydroxymethyl)methylamino]propane	Bis-Tris Propane	Sigma Aldrich (St. Louis, MO)
Betaine		Sigma Aldrich (St. Louis, MO)
Tween-20		Sigma Aldrich (St. Louis, MO)
Sodium dodecyl sulfate	SDS	Sigma Aldrich (St. Louis, MO)
Ethylenediaminetetraacetic acid	EDTA	Sigma Aldrich (St. Louis, MO)
Sodium chloride	NaCl	Sigma Aldrich (St. Louis, MO)
Tris base	Tris	Sigma Aldrich (St. Louis, MO)
Hydrochloric acid	HCl	Sigma Aldrich (St. Louis, MO)
2',4',6'-Trihydroxyacetophenone monohydrate	THAP	Sigma Aldrich (St. Louis, MO)
Sodium hydroxide	NaOH	Sigma Aldrich (St. Louis, MO)
Ethanol	EtOH	Sigma Aldrich (St. Louis, MO)
Triethylamine acetate (2.0 M)	TEAA	Sigma Aldrich (St. Louis, MO)
5-Azidopentanoic acid	5-APA	Sigma Aldrich (St. Louis, MO)
Copper(II) sulfate	CuSO4	Sigma Aldrich (St. Louis, MO)
Disodium hydrogen phosphate	Na2HPO4	Sigma Aldrich (St. Louis, MO)
Sodium phosphate monobasic monohydrate	NaH2PO4	Sigma Aldrich (St. Louis, MO)
TentaGel M NH2 (10 µm monosized)		Rapp Polymere (Tübingen, Germany)
TentaGel MB RAM (200 µm monosized)		Rapp Polymere (Tübingen, Germany)
(S)-2-((((9H-Fluoren-9-yl)methoxy)carbonyl)amino)-5-(3-((2,2,4,6,7-pentamethyl-2,3-dihydrobenzofuran-5-yl)sulfonyl)guanidino)pentanoic acid	Fmoc-Arg(pbf)-OH	Combi-Blocks (San Diego, CA)
Tris[(1-benzyl-1H-1,2,3-triazol-4-yl)methyl]amine]	TBTA	Combi-Blocks (San Diego, CA)
2-((((9H-Fluoren-9-yl)methoxy)carbonyl)amino)acetic acid	Fmoc-Gly-OH	Ambeed, Inc. (Arlington Hts, IL)
1-Ethyl-3-(3-dimethylaminopropyl)carbodiimide	EDC	Oakwood Chemicals (Estill, SC)
(2S)-2-({[(9H-fluoren-9-yl)methoxy]carbonyl}amino)pent-4-ynoic acid	Fmoc-Pra-OH	Millipore Sigma (Burlington, MA)
96 well MultiScreen Solvinert Filter Plate, Hydrophilic, 0.45 µm		Millipore (Billerica, MA)
Dimethyl sulfoxide	DMSO	VWR (Radnor, PA)
Mobicol Spin Columns		Boca Scientific (Dedham, MA)
Large 10-µm Pore Size Frit		Boca Scientific (Dedham, MA)
Syringes (25 mL - 1 mL)		Fisher Scientific (Hampton, NJ)
Syringe frits (25 mL - 1 mL)		Biotage (Uppsala, Sweden)
Easy Glide Blunt Needle (18 G)		Global Medical Products (Port St Lucie, FL)
Biopsy Punch		World Precision Instruments, Inc. (Sarasota, FL)
Safe Soap		Gold Biotechnologies Inc. (St. Louis, MO)
Norland Optical Adhesive 71	NOA71	Norland Products (Jamesburg, New Jersey)
384-well Black Plate, Low Volume		Greiner Bio-One (Monroe, NC)
Plastic Syringes		BD Medical (Franklin Lakes, NJ)
PTFE Coated Magnets		V&P Scientific Inc. (San Diego, CA)
Leur-lock Needle (30 gauge)		Ellsworth Adhesives (Germantown, WI)
Tygon Tubing (0.01 in. i.d. X 0.03 in. o.d.)		Saint Gobain (Valley Forge, PA)
3-hydroxytriazolo[4,5-b]pyridine	HOAt	APExBio (Houston, TX)
Microcentrifuge tube (polypropylene, clear, 2 mL)		Axygen (Union City, CA)
Taq DNA polymerase	*Taq*	New England Biolabs (Ipswich, MA)
10X Standard *Taq* Buffer		New England Biolabs (Ipswich, MA)
T4 DNA Ligase (400,000 U/mL)		New England Biolabs (Ipswich, MA)
NcoI-HF (20,000 U/mL)		New England Biolabs (Ipswich, MA)
pF3A WG (BYDV) Flexi® Vector		Promega Corp. (Milwaukee, WI)
DH5α Competent Cells		New England Biolabs (Ipswich, MA)
PT7CFE Vector		ThermoFisher Scientific (Waltham, MA)
Flexi® Enzyme Blend (*SgfI* & *PmeI*)		Promega Corp. (Milwaukee, WI)
NdeI (20,000 U/mL)		New England Biolabs (Ipswich, MA)
2′-Deoxyribonucleotide triphosphate (dATP, dTTP, dGTP, dCTP)	dNTPs	Promega Corp. (Milwaukee, WI)
Acrylamide/Bis 19:1 (40% (w/v) solution)		Bio-rad (Hercules, CA)
Ammonium persulfate	APS	Bio-rad (Hercules, CA)
Tetramethylethylenediamine	TEMED	Bio-rad (Hercules, CA)
Hard-Shell® 96-Well PCR Plates, (low profile, thin wall, skirted)		Bio-rad (Hercules, CA)
Mini-PROTEAN® Tetra Vertical Electrophoresis Cell (4-gel, for 1.0 mm thick handcast gels)		Bio-rad (Hercules, CA)
Tris-Borate-EDTA Buffer, 5X	5X TBE	Biotium (Fremont, CA)
15 ml Conical Centrifuge Tubes (polypropylene)		Genesee Scientific (San Diego, CA)
150 µm Celltrics Filter		Sysmex (Irvine, CA)
Coding oligonucleotides (5' phosphorylated, /5Phos/)		Integrated DNA Technologies, Inc (Coralville, IA)
Amino-modified DNA headpiece (/5Phos/GAGTCA/iSp9//iUniAmM//iSp9/TGACTCCC)	NH_2-HDNA	Integrated DNA Technologies, Inc (Coralville, IA)
Waters 4.6 x 150 mm 3.5 µm BEH XBridge		Thomas Scientific
Pico-Surf™ fluorosurfactant (5% solution in Novec-7500™)		Sphere Fluidics (Cambridge, UK)
Novec-7500™	HFE	3M (Saint Paul, MN)
3-[(3-Cholamidopropyl)-dimethylammonio]-1-propanesulfonate hydrate	CHAPS	G-Biosciences (St. Louis, MO)
[5-(and-6)-Carboxytetramethylrhodamine]	TMR	AnaSpec (Fremont, CA)
Poly(dimethylsiloxane)	PDMS	Dow Corning (Midland, MI)
Aquapel		Pittsburgh Glass Words (Cranberry Township, PA)
TnT® SP6 High-Yield Wheat Germ Protein Expression System		Promega (Madison, WI)
Dextran 70,000 MW		Sigma Aldrich (St. Louis, MO)
MagBio HighPrep PCR		MagBio Genomics (Gaithersburg MD)
2,4,6-Trimethylpyridine	TMP	Sigma Aldrich (St. Louis, MO)
1-Hydroxy-7-azabenzotriazole	HOAt	Sigma Aldrich (St. Louis, MO)
PF-06446846 hydrochloride	PF846	Sigma Aldrich (St. Louis, MO)
ZymoPure II Plasmid Midi Prep Kit		Zymo Research (Tustin, CA)
Phosphate-Buffered Saline Tablets	PBS	ThermoFisher Scientific (Waltham, MA)
Tris-Buffered Saline Tablets	TBS	ThermoFisher Scientific (Waltham, MA)
Triton X-100		Sigma Aldrich (St. Louis, MO)
cOmplete mini Protease Inhibitor Cocktail		Sigma Aldrich (St. Louis, MO)
12-well Tissue Culture Treated Plates, Clear		Corning (Corning, NY)
Turbo DNAse (2 U/µL)		ThermoFisher Scientific (Waltham, MA)
Dulbecco's Modified Eagle Medium	DMEM	Corning (Corning, NY)
Fetal Bovine Serum	FBS	Sigma Aldrich (St. Louis, MO)
Penicillin-streptomycin (10,000 U/mL)		ThermoFisher Scientific (Waltham, MA)
100X Non-Essential Amino Acid Mixture	NEAA	ThermoFisher Scientific (Waltham, MA)
4-20% mini-PROTEAN TGX gel		Bio-rad (Hercules, CA)
0.2 µm Nitrocellulose Membrane		Bio-rad (Hercules, CA)
Nonfat Dry Milk		Lab Scientific (Danvers, MA)
Direct-Blot™ HRP anti-GAPDH Antibody		BioLegend (San Diego, CA)
PCSK9 Rabbit Monoclonal Antibody		Abcam (Cambridge, UK)
Anti-rabbit HRP-linked Antibody		Cell Signaling Technology (Danvers, MA)
ECL Western Blotting Substrate		ThermoFisher Scientific (Waltham, MA)
Bovine Serum Albumin	BSA	Sigma Aldrich (St. Louis, MO)

Buffers

Buffer	Abbreviation	Recipe
Click Reaction Buffer	CRB	50% DMSO<br>30 mM TEAA<br>0.04% Tween-20<br>pH 7.65
Bis-Tris-Propane Wash Buffer	BTPWB	50 mM NaCl<br>0.04% Tween 20<br>10 mM Bis-Tris<br>pH 7.6
Bis-Tris-Propane Breaking Buffer	BTPBB	100 mM NaCl<br>1% SDS<br>1% Tween 20<br>10 mM Bis-Tris<br>pH 7.6
10X Bis-Tris-Propane Ligation Buffer	10X BTPLB	500 mM NaCl<br>100 mM $MgCl_2$<br>10 mM ATP<br>0.2% Tween 20<br>100 mM Bis-Tris propane<br>pH 7.6
qPCR Buffer		1 M Betaine<br>6% DMSO<br>0.2X SYBR Green<br>0.2 mM each dNTP
Crush and Soak Buffer	C&S	500 mM NaCl<br>1 mM EDTA<br>10 mM Tris<br>pH 7.6
20% PEG Binding Buffer		20% PEG8000<br>2.5 M NaCl<br>10 mM Tris-HCl<br>1 mM EDTA<br>0.05% Tween-80<br>pH 8
PBS-T		1X PBS<br>0.1% Tween-20<br>pH 7.5
RIPA Buffer		50 mM Tris-HCl<br>150 mM NaCl<br>0.1% SDS<br>0.1% Triton-X-100<br>0.5% sodium deoxycholate<br>1X Complete protease inhibitor cocktail<br>pH 8

Methods

Bifunctional Resin Synthesis and Characterization

Bifunctional resin was prepared similarly to previous work.[S1] Briefly, TentaGel Rink amide resin (200-µm, 200 mg; 10-µm, 300 mg) was transferred to a fritted syringe (10 mL) and swelled in DMA (1 h, RT). Linker construction proceeded via iterative cycles of manual solid-phase peptide synthesis. Each cycle included: (1) 9-fluorenylmethoxycarbonyl (Fmoc) deprotection (2×15 min, RT, 8 mL, 20% 4-MePip); (2) N-α-Fmoc-amino acid (1.4 mmol) activation with DIC/HOAt/DIEA (1.4 mmol/1.4 mmol/2.8 mmol; 9.3 mL DMA), and incubation (2 min, RT); (3) N-α-Fmoc-amino acid coupling to resin by transferring activated acid (9.3 mL) to resin and incubating with rotation (1 h, 50 °C, 8 rpm). After each deprotection and coupling step, reactants were expelled and the resin was washed ($3 \times$ DMA; $3 \times$ DCM; $3 \times$ DMA). N-α-Fmoc-Lys(Mca)-OH, N-α-Fmoc-Arg(pbf)-OH, N-α-Fmoc-Gly-OH, N-α-Fmoc-Pra-OH, and N-α-Fmoc-Gly-OH were coupled sequentially to 200-µm resin. The 200-µm resin was combined with 10-µm resin and synthesis proceeded with N-α-Fmoc-Gly-OH, N-α-Fmoc-Pra-OH, and N-α-Fmoc-Gly-OH coupling to the mixed-scale resin. After N-α-Fmoc-Lys(Mca)-OH and the first N-α-Fmoc-Gly-OH couplings, unreacted amine sites were capped (20% acetic anhydride, 30 min, RT, 8 mL). The resin was washed ($3 \times$ DMA; $3 \times$ DCM; $3 \times$ DMA) and an aliquot (0.05 mg) was transferred to a clean fritted Mobicol spin column, washed ($2 \times$ DCM), and dried *in vacuo*. Cleavage cocktail (95:2.5:2.5 TFA/TIPS/H_2O, 300 µL) was added to the dried resin and incubated (30 min, RT, 8 rpm). Cleaved linker was expelled, concentrated *in vacuo*, resuspended (1:1 0.1% TFA in H_2O/ACN v/v, 50 µL), and yield was determined using reversed-phase LC (Eclipse XDB-C18, 4.6×15 mm 3.5-µm, Agilent) with gradient elution (mobile phase A= HPLC H_2O, 0.1% TFA; mobile phase B = ACN; 5–85% B over 5 min), UV absorbance detection (λ = 320 nm). The singly charged $[M + H]^+$ ion (m/z = 949) was isolated with collision-induced dissociation enabled (5 keV, ISQ™ EM Single Quadrupole

Mass Spectrometer, ThermoFisher Scientific).

Azido Headpiece DNA Synthesis, Purification, and Characterization

Amine headpiece DNA (NH_2-HDNA, 0.5 µmol) was suspended in DI H_2O (250 µL). 5-azidopentanoic acid NHS ester was prepared by dissolving NHS (20 µmol), EDC (20 µmol) and 5-APA (30 µmol) in DMF (70 µL) and incubating with rotation (30 min, 65 °C, 8 rpm). NH_2–HDNA acylation reaction was assembled by combining 5-azidopentanoic acid NHS ester solution (70 µL) with NH_2-HDNA (0.5 µmol), DI H_2O (292 µL) and phosphate buffer (1 M, pH 8.0, 400 µL) in a 15-mL conical tube and vortexed. The reaction was incubated (2 h, RT, 8 rpm). A fresh solution of 5-azidopentanoic acid N-hydroxysuccinimidyl ester was prepared as described above, added to the acylation reaction, and the reaction was incubated (2 h, RT, 8 rpm). The reaction was quenched (1 M Tris, pH 7.6, 100 µL) and incubated (5 min, 60 °C, 8 rpm). Azido-HDNA (N_3–HDNA) product was combined with NaOAc solution (3 M, pH 5.2, 1 mL) and linear polyacrylamide (15 µL), and precipitated twice in ethanol (16 h, -20 °C, 5 volumes). Supernatant was removed and the pellet was suspended (15 µL linear polyacrylamide, 1 mL DI H_2O) and precipitated (30 min, -20 °C, 5 volumes). The pellet was resuspended (500 µL DI H_2O) and yield was determined using reversed-phase HPLC (X-Bridge BEH C18 column, 10 mm $\times$ 150 mm, 130 Å, 5 µm, Waters Corp., Milford, MA) with gradient elution (mobile phase A = HPLC H_2O, 20 mM TEAA, pH 8; mobile phase B = ACN; 5–20% B over 22 min). Relative peak AUC between starting material and product peaks was used to calculate yield. A product fraction aliquot (1 µL) was spotted to a MALDI-TOF MS target plate, dried, covered with matrix solution comprising 2',4',6'-trihydroxyacetophenone monohydrate (18 mg/mL) and ammonium citrate dibasic (7 mg/mL) prepared in 1:1 ACN/0.1% TFA, and mass analyzed via MALDI-TOF MS (Microflex, Bruker Daltonics Inc., Billerica, MA).

Encoded Library Synthesis

DNA-encoded library resin barcoding

Library synthesis and handling were carried out in a UV-free room. DNA-encoded solid phase synthesis (DESPS) has been previously described.[S1,S2] Oligonucleotides are indicated in bold with ”≈”. Numeric identifiers have been previously described.[S3] Sequences are included in **Coding Oligonucleotides**. Stock solutions of oligonucleotide pairs (OP) were prepared with complementary oligonucleotides (60 µM [+], 60 µM [-], 50 mM NaCl, 1 mM Bis-Tris pH 7.6), which were heated (5 min, 60 °C) and cooled to ambient (5 min, RT) before each use. OP stocks are designated with [±], indicating ”double-stranded.”

Cycle 1 Chemistry

Mixed-scale resin was washed (3 × DMA) and Fmoc was removed (20% 4-MePip, 2 × 15 min, RT, 8 mL). Resin was washed (3 × DMA; 3 × DCM; 3 × DMA; 3 × DMA) prior to acylation. Resin was split into 14 × 6-mL syringes (12 × 40 mg, 11.2 µmol; 1 × 20 mg, 5.6 µmol cycle 1 BB). Activation mix was prepared by combining DIC (143 µmol), HOAt (143 µmol) and DIEA (286 µmol) in reaction solvent (DMA, 3.57 mL). Activation mix was incubated (5 min, RT) and aliquotted to separate tubes containing cycle 1 BBs (5 eq, 56 µmol). Reaction mix was aspirated into the respective syringe aliquots and incubated (2 h, 37 °C, 8 rpm). Reaction mix was expelled from the syringe and the resin was washed (3 × DMA; 3 × DCM; 3 × DMA). A small aliquot of resin (0.05 mg) was removed from each coupling and placed into separate fritted spin columns. Resin aliquots were dried (DCM, *in vacuo*). Cleavage cocktail (95:2.5:2.5 TFA/TIPS/H_2O, 300 µL) was incubated with the resin (30 min, RT, 8 rpm) then expelled, concentrated *in vacuo*, resuspended (1:1 0.1% TFA/ACN v/v, 50 µL) and analyzed using LC-MS as described.

HDNA Click and Characterization

Resin aliquots were further split into 2 aliquots (24 × 20 mg, 5.6 µmol) and were equilibrated with CRB (1 mL, 1 h, RT). Each aliquot was suspended in sparged CRB. Catalyst mix was prepared by combining $CuSO_4$ (169 µmol), TBTA (2.86 µmol), and ascorbic acid (819 µmol) in DMSO (3.12 mL). Catalyst mix was incubated (5 min, 37 °C) and added to each aliquot of suspended resin (120 µL), mixed, and incubated (5 min, 37 °C, 15 rpm). N_3–HDNA (630 nmol) and ascorbic acid (1.76 µmol) were dissolved in TEAA buffer (100 mM, pH 7.5, 1.18 mL) and N_3–HDNA solution was added to ach resin aliquot. The reaction was immediately mixed and incubated with rotation (2 h, 37 °C, 15 rpm). Resin was centrifuged (30 s, 1000 × g), the supernatant removed, the resin washed (6 × BTPBB with 100 mM EDTA) and incubated with rotation (16 h, 37 °C, 15 rpm). Resin was washed (3 × BTPWB) and a small aliquot was removed for ligation and qPCR analysis (0.1 mg, 0.11 nmol HDNA). The resin aliquot was transferred to a first enzymatic oligonucleotide ligation reaction, consisting of ≈**OS[**±**]** (0.3 nmol), ≈**1101[**±**]** (0.3 nmol), ≈**2202[**±**]** (0.3 nmol), ≈**1303[**±**]** (0.3 nmol), and T4 DNA ligase (2 µg), was combined in BTPLB (50 µL) and aliquoted to bifunctional–HDNA resin. Resin samples were incubated with rotation (4 h, 18 °C, 8 rpm), then washed (6 × BTPWB). A second ligation reaction, consisting of ≈**2404[**±**]** (0.3 nmol), ≈**1505[**±**]** (0.3 nmol), ≈**2606[**±**]** (0.3 nmol), ≈**RS[**±**]** (0.3 nmol), and T4 DNA ligase (2 µg) was assembled in BTPLB (50 µL) and aliquoted to bifunctional–HDNA resin. Resin samples were incubated with rotation (4 h, 18 °C, 8 rpm), then washed in aqueous (6 × BTPWB) and organic solvent (1 × DMA, 1 h) and transferred to aqueous buffer (BTPWB). Quantitative PCR (qPCR) mixture contained *Taq* DNA polymerase (0.05 U/µL), oligonucleotide primers 5′-GCCGCCCAGTCCTGCTCGCTTCGCT AC-3′ and 5′-GTGGCACAACAACTGGCGGGCAAAC-3′ (0.2 µM each), and SYBR Green (0.2X, Life Technologies) in qPCR buffer (1X). Single resin particles (200 µm) and 100-bead aliquots (10 µm) in BTPWB (1 µL) were added to separate amplification reaction wells (20 µL, 3 replicates for bifunctional 200-µm resin sample, 3 replicates for bifunctional 10-µm

resin sample). Resin supernatant (1 µL) was added to respective negative control reaction wells (20 µL). Template standards (1 fmol, 100 amol, 10 amol, 1 amol, 100 zmol in BTPWB) were added to separate reaction wells (20 µL). The reaction plate was thermally cycled (95 °C, 2 min; [95 °C, 20 s; 68 °C, 30 s, 72 °C, 30 s] $\times$ 30 cycles) with fluorescence monitoring (CFX-96 Real-Time System, Bio-Rad). Samples were quantitated (CFX Manager, version 3.1, Bio-Rad) using baseline correction Cq determination mode. Supernatant background was subtracted from respective single-particle measurements. Background-subtracted replicates were averaged for molecule per bead (MPB) counts and % relative standard deviation was calculated.

Opening Sequence Ligation

Characterized resin was incubated in BTPLB (1 h, RT). Ligation mix was prepared by combining **OS [±]** (2.1 µmol) and T4 DNA ligase (81 µg) in BTPLB (22.46 mL) on ice. Buffer was removed from resin and ligation mix (1 mL) was added to each tube. Resin was incubated (16 h, 18 °C, 600 rpm), then ligation mix was removed. Each aliquot of resin was washed (6 $\times$ BTPWB).

Cycle 1 Encoding

Each aliquot of resin was pooled by cycle 1 BB (BTPWB, 800 µL). Each aliquot was combined with BTPWB (1 mL) and incubated (3 h, RT). Each aliquot of characterized resin was split equally into 8 wells of a 96-well MultiScreen Solvinert Filter Plate (5 mg resin/ 100 µL, 1.4 µmol). Ligation master mix was prepared by combining T4 DNA ligase (130 µg) with BTPLB (14 mL) on ice. ≈**11xx[±]** oligonucleotide tags (11 nmol, 150 µM stock) were added to respective wells and combined with ligation master mix (130 µL, total volume = 300 µL). Resin was incubated (4 h, 18 °C, 600 rpm), then ligation mix was removed by vacuum filtration. Wells were washed (6 $\times$ BTPWB).

Each resin sample was washed and all resin samples were pooled by cycle 1 BB (1:1

DCM/DMA v/v, 800 µL). Each aliquot was combined with BTPWB (1 mL) and incubated (3 h, RT). Each aliquot was split equally into 8 wells of a 96-well MultiScreen Solvinert Filter Plate (5 mg resin/ 100 µL, 1.4 µmol). Ligation master mix was prepared by combining T4 DNA ligase (130 µg) with BTPLB (14 mL) on ice. ≈**22xx[±]** oligonucleotide tags (11 nmol, 150 µM stock) were added to respective wells and combined with ligation master mix (130 µL, total volume = 300 µL). Resin was incubated (4 h, 18 °C, 600 rpm), and ligation mix was removed by vacuum filtration. Wells were washed (6 × BTPWB).

Each well of resin was washed from the well and pooled by cycle 1 BB (1:1 DCM/DMA v/v, 800 µL). Each aliquot was combined with BTPWB (1 mL) and incubated (3 h, RT). Each aliquot was split equally into 8 wells of a 96-well MultiScreen Solvinert Filter Plate (5 mg resin/ 100 µL, 1.4 µmol). Ligation master mix was prepared by combining T4 DNA ligase (130 µg) with BTPLB (14 mL) on ice. ≈**13xx[±]** oligonucleotide tags (11 nmol, 150 µM stock) were added to respective wells and combined with ligation master mix (130 µL, total volume = 300 µL). Resin was incubated (4 h, 18 °C, 600 rpm) and ligation mix was removed by vacuum filtration. Wells were washed (6 × BTPWB).

Cycle 2 (24xx, 15xx) Encoding

Resin from all of the wells was pooled in a 25 mL fritted syringe (1:1 DCM/DMA v/v, 10 mL) and mixed. The solution was expelled and resin was combined with BTPWB (10 mL) and incubated (3 h, RT). Resin was washed (3 × BTPWB) and aliquotted equally among (4) 96-well MultiScreen Solvinert Filter Plates (1.25 mg resin/ 25 µL, 1.4 nmol HDNA sites) and dried by vacuum filtration. Resin was suspended in DI H_2O (25 µL). Ligation master mix was prepared by combining T4 DNA ligase (160 µg) with BTPLB (14 mL) on ice. ≈**24xx[±]** and ≈**15xx[±]** oligonucleotide tags (3 nmol, 150 µM stock) were added to respective wells. Ligation master mix (35 µL, total volume = 100 µL) was added to each well. Resin was incubated (4 h, 18 °C, 600 rpm) and ligation mix was removed by vacuum filtration. Wells were washed (6 × BTPWB).

Cycle 2 Chemistry

Resin in each well was suspended in DMA (100 µL). Acylation reaction was prepared by adding cycle 2 BBs (5 µmol in 100 µL DMA) to their respective wells. Activation mix was prepared by combining DIC (4.8 mmol), HOAt (4.8 mmol) and DIEA (9.6 mmol) in DMA (40 mL). Activation mix was incubated (5 min, RT) and aliquotted to each well containing acids (100 µL). Plates were covered with an adhesive aluminum film and incubated(1.25 h, 37 °C, 600 rpm). Reaction mix was removed via vacuum filtration. Resin was washed (3 × DMA; 3 × DCM; 3 × DMA).

Bead-Specific Barcoding (26xx)

Resin in all of the wells was pooled in a 25-mL fritted syringe (1:1 DCM/DMA v/v, 10 mL) and mixed. The solution was expelled and resins were combined with BTPWB (10 mL) and incubated (3 h, RT). Resin was washed (3 × BTPWB), resuspended in DI H_2O (9.6 mL) and aliquotted equally into each well of a 96-well MultiScreen Solvinert Filter Plate (5 mg resin/ 100 µL, 1.4 µmol). Ligation master mix was prepared by combining T4 DNA ligase (120 µg) with BTPLB (10 mL) on ice. ≈**26xx[±]** oligonucleotide tags (12.8 nmol, 150 µM stock) were added to respective wells and combined with ligation master mix (115 µL, total volume = 300 µL). Resin was incubated (4 h, 18 °C, 600 rpm), then ligation mix was removed by vacuum filtration. Wells were washed (6 × BTPWB).

Library Quality Control

qPCR library QC proceeded as previously described.[S1,S2] A qPCR matrix contained *Taq* DNA polymerase (0.05 U/µL), oligonucleotide primers 5′-GCCGCCGCCTTCGTCCTTC TCAGCGAC-3′ (0.2 µM) and 5′-GTGGCACAACAACTGGCGGGCAAAC-3′ (0.2 µM), and qPCR buffer (1X). Individual 200-µm library QC beads (BTPWB, 1 µL) were added to separate amplification wells containing qPCR matrix (20 µL, 20 QC beads). Supernatant

for each resin sample (1 µL) was added to separate amplification wells (20 µL, 2 replicates). Template standard solutions (1 fmol, 100 amol, 10 amol, 1 amol, 100 zmol, each in 1 µL BTPWB) were added to separate amplification reactions (20 µL). Reactions were thermally cycled (95 °C, 2 m; [95 °C, 20 s; 62 °C, 30 s, 72 °C, 30 s] × 30 cycles) with fluorescence monitoring (channel 1, CFX96 Real-Time System, Bio-Rad) and quantitated (CFX Manager, Version 3.1, Bio-Rad, baseline subtracted).

Single library QC beads (20) were retrieved via pipet from PCR plate wells and deposited into a 96-well microplate (MeOH, 0.1 mL). Each library QC bead PCR sample (10 µL) was purified by native PAGE (6%, 1 × TBE, 12 W, 30 min). Gel slices containing 138-bp DNA products were excised, suspended in C&S (0.1 mL), and incubated (16 h, RT, 8 rpm). A qPCR matrix contained *Taq* DNA polymerase (0.05 U/µL), oligonucleotide primers 5′-GTTTTCCCAGTCACGAC-3′ (0.3 µM), 5′-GTGGCACAACAACTG-3′ (0.28 µM), and 5′-CGCCAGGGTTTTCCCAGTCACGACCAACCACCCAAACCACAAACCC AAACCCCAAACCCAACACACAACAACAGCCGCCGCCTTCGTCCTTCTCAGCGA C-3′ (0.02 µM, FOX primer), and qPCR buffer (1X). PAGE-purified PCR products (0.5 µL) were added to separate amplification reactions (20 µL) and thermally cycled (95 °C, 2 min; [95 °C, 20 s; 68 °C, 30 s; 72 °C, 30 s] × 15 cycles. PCR sample (10 µL) was purified by native PAGE (6%, 1 × TBE, 12 W, 30 min). Gel slices containing 221-bp DNA products were excised, eluted in C&S as described, and sequenced using the primer 5′-GTTTTCC CAGTCACGAC-3′.

Sequencing reads were trimmed to remove all called bases prior to the opening primer sequence (5′-GCCGCCGCCTTCGTCCTTCTCAGCGAC-3′). Sequences were aligned to a degenerate reference sequence (5′-GCCGCCGCCTTCGTCCTTCTCAGCGACATGGNN NNNNNNTCANNNNNNNNGTTNNNNNNNNCTANNNNNNNNTTCNNNNNNNN NCGCNNNNNNNNGCCTCCCAAAC-3′) and the encoding regions (5′-NNNNNNNN-3′) were matched to the BB alpha-numeric identifier lookup table to assign the synthesis history for each compound (Table S3).

Resin cleavage and MALDI-TOF MS analysis were previously described.[S1,S2] Briefly, individual library QC beads (MeOH, 0.1 mL) were dried *in vacuo* (60 °C). Cleavage cocktail (95:2.5:2.5 TFA/TIPS/DCM v/v, 50 µL) was added to dried single library QC bead samples. Cleavage reactions were incubated with shaking (2 h, RT, 100 rpm) then dried *in vacuo* (60 °C). Compound was resuspended (1:1 ACN/0.1% TFA in H_2O v/v, 50 µL), and an aliquot (1 µL) was spotted to a MALDI-TOF MS target plate, dried, covered with HCCA matrix solution (1 µL), dried, and analyzed via MALDI-TOF MS (Microflex, Bruker Daltonics, Inc., Billerica, MA, Figure S2).

HygB Control Bead Synthesis

Synthesis QC beads were prepared by transferring Tentagel Rink Amide resin (160 µm dia., 0.4 mmol/g, 120 mg) to a fritted syringe (25 mL) and swelling in DMF (1 h, RT). Fmoc was removed (20% piperidine in DMF, 5 mL, 1 x 5 min, 1 x 15 min, RT, 8 rpm) and resin was washed (3 × DMF; 3 × DCM; 3 × DMF). N-α-Fmoc-K(Mca)-OH (150 µmol) was activated with COMU/DIEA (300/150 µmol) in DMF (1 mL), combined with resin, and incubated (1 h, 50 °C, 8 rpm). The resin was washed (3 × DMF; 3 × DCM; 3 × DMF). Unreacted amines were capped with acetic anhydride (5 mL, 20% in DMF, 30 min, 50 °C, 8 rpm, 2 ×) and the resin was washed (5 × DMF; 5 × DCM; 5 × DMF). Fmoc was removed (20% piperidine in DMF, 5 mL, 1 × 5 min, 1 × 15 min, RT, 8 rpm) and resin was washed (3 X DMF; 3 X DCM; 3 X DMF). N-α-Fmoc-R(Pbf)-OH (150 µmol) was activated with COMU/DIEA (300/150 µmol) in DMF (1 mL), combined with resin, and incubated (1 h, 50 °C, 8 rpm, 2 ×). The resin was washed (3 × DMF; 3 × DCM; 3 × DMF).

Screening resin (TentaGel amino-functionalized, monosized 10 µm dia., 0.26 mmol/g, 100 mg) was added to a syringe (25 mL) fitted with two frits (10 µm polyethylene, 20 mm dia.), combined with DMF (5 mL, 1 h, RT, 8 rpm), and incubated. An aliquot of synthesis QC resin (50 mg) was transferred to the syringe. Fmoc was removed (20% piperidine in DMF, 5 mL, 1 x 5 min, 1 x 15 min, RT, 8 rpm) and resin was washed (3 × DMF; 3 ×

DCM; 3 × DMF). Photocleavable linker (25 µmol) was activated with HOAt/DIC/TMP (25/30/60 µmol) in DCE/DMA (150 µL, 50/50), added to the resin, and incubated (1 h, 50 °C, 8 rpm). The resin was washed (3 × DMF; 3 × DCM; 3 × DMF). HygB solution was prepared (1 M in H_2O, 250 µL) was combined with mixed scale resin and incubated (16 h, 50 °C, 8 rpm). The resin was washed (3 × DMF; 3 × DCM; 3 × DMF).

Microfluidic Device Fabrication

Microfluidic PDMS devices were prepared from a master wafer using soft lithography.[S4] Feature heights (30, 50, and 600 µm) were measured with a stylus profilometer (DektakXT, Bruker, Billerica, MA). PDMS prepolymer (44 g, 10:1 base/curing agent) was degassed *in vacuo*, poured over the master mold, polymerized (1 h, 80 °C), cooled (10 min, RT), and peeled. Channel ports were punched with a biopsy punch (0.75 mm dia). Degassed PDMS prepolymer (2 g) was placed on glass slides, spun using a spin coater (500 rpm, 30, Brewer Scientific), and cured (1 h, 80 ºC). PDMS molds and PDMS-coated slides were cleaned with a 2% Safe-Soap solution, rinsed with DI H_2O and isopropyl alcohol, plasma treated (300 mTorr, 60 s, Harrick Plasma), and bonded. Before use, the droplet generation and sorting regions were treated with Aquapel, incubated (30 s), and dried with N_2. Integrated waveguides were fitted into devices and secured with cured NOA71 (30 min, long wave UV) as previously described.[S5] The waveguide calibration channel was filled with trimethylsiloxy-terminated PDMS.

Microfluidic Device Operation and Data Acquisition

Aqueous solutions (AQ1, AQ2) and oils (OIL1, OIL2, OIL3) were loaded into syringes (1/1/1/10/10 mL, respectively) that were fitted with blunt-tip Luer-Lok needles. AQ1 syringes were also loaded with a PTFE-coated magnet (3 mm dia.) when beads were included in the experiment. The magnet was agitated periodically (∼20 min) throughout

the droplet experiment to homogenize the bead suspension. Syringes were connected to fluidic inputs with microbore Tygon tubing (0.01″ x 0.03″ IC × OD). Droplet generation oil (OIL1) was 1% picosurf in HFE. Spacing (OIL2) and pinning (OIL3) oils were neat HFE. Assay reagents were driven by displacement syringe pumps (Legato 100, KD Scientific, Holliston, MA) through the circuit. OIL2 and OIL3 were initially flowed (22 µL/min and 12 µL/min, respectively) and hit and waste shunt tubing were clamped to backfill the circuit. Once the incubator was filled with oil, the hit and waste tubing were unclamped and back shunts were clamped. AQ1, AQ2, and OIL1 flows were initiated (960 nL/min, 640 nL/min, and 1200 nL/min, respectively) and held constant during the screen. Once droplet generation equilibrated (∼10 min), the main shunt was clamped and droplets started to fill the incubator. When droplets filled the incubator and entered the sorting region, data acquisition and screening was initiated.

The custom confocal LIF detection setup and LabView code were previously described.[S3] Briefly, photon counting PMTs (H7828, Hamamatsu, Middlesex, NJ) were used to detect droplet fluorescence emission using a custom two-channel (520 and 570 nm) confocal microscope with laser excitation (488 nm, 20 mW, OBIS-488 20LS, Coherent Inc., Santa Clara, CA). A data acquisition board (DAQ, NI, USB-6341, National Instruments) was used to digitize PMT signal, which was binned into counts, and in-house LabView code was used to control acquisition and execute droplet sorting decisions. Signal was smoothed in real-time using median-filter smoothing (width = 3). Droplets were identified by the 570 nm channel signal and the maximum signal in the 520 nm channel (max = $counts_{n-1}$ when $counts_n < counts_{n-1}$) was set as the droplet peak value. A roaming sort threshold was used to identify droplets with statistically significantly reduced droplet fluorescence in the 520 nm channel. The threshold was calculated as $\mu - 5\sigma$, where μ and σ were the mean and standard deviation of the previous 300 droplets' fluorescence values, respectively. Hit droplets were not included in the calculation of droplet mean and standard deviation. A detected hit droplet (droplet fluorescence $< \mu - 5\sigma$ counts), LabView output a TTL pulse

from the DAQ board to a waveform generator (Agilent 33210A, Agilent Technologies, Santa Clara, CA), triggering a square wave pulse output (5V, 10 kHz, 800 cycles) that was amplified (gain = 100 V/V, TREK Model 2210 high-voltage power amplifier, TREK Inc., Lockport, NY) and conducted through liquid metal electrode channels (VAC).

Droplet Incubation Analysis

Droplets containing low and high dye concentrations were generated by alternating aqueous input flow rates. Both inputs contained TMR (300 nM) and a low concentration of fluorescein (300 fM) in buffer (10 mM Tris-HCl, pH 7.5, 0.02% CHAPS, 10.7% dextran 70 kDa). AQ1 contained additional fluorescein (200 nM). Flow rates were initially set at 3:1 (AQ1:AQ2, 1200 nL/min and 400 nL/min, respectively) and were alternated to transition from high-to-low fluorescence droplets. The rates were reversed for low-to-high fluorescence transitions. OIL1 was kept constant (1200 nL/min) throughout the experiment. Analysis of incubation time was performed as described.[S6]

Droplet-Scale Flow Injection Analysis of WG IVTT Assay Quality

WG lysate (AQ2) containing 0.02% CHAPS was used in all droplet conditions. AQ1 for DMSO vehicle control droplets contained 0.02% CHAPS, 2 µM TMR, 62.5 ng/µL PCSK9[1:33]-GFP plasmid, 9% dextran, and 0.25% DMSO. AQ1 for PF846 positive control droplets contained 0.02% CHAPS, 2 µM TMR, 62.5 ng/µL PCSK9[1:33]-GFP plasmid, 9% dextran, and 250 µM PF846. Droplet data were acquired for 30 min for each condition.

Library Screening in Droplets

DEL bead screening was performed as previously described,[S6,S7] with some modifications. DEL beads ligated with ≈**RS[±]** closing primer were filtered through a 20-µm Celtrics filter and a previously described microfluidic filter device,[S7] then counted with a

hemocytometer. AQ1 contained 0.02% CHAPS, 2 μM TMR, 9% dextran, and DEL beads (7̃00 beads/μL). AQ1 for all droplet experiments contained 62.5 ng/μL plasmid, except for RPL27[1:32]-GFP and $KRAS^{G12D}$[1:189]-GFP, which contained 50 and 150 ng/μL plasmid, respectively. AQ2 contained WG lysate with 0.2% CHAPS.

Hit Bead Preparation for Illumina High-Throughout Sequencing

Hit beads were collected into Costar Spin-X filters (0.22 μm, cellulose acetate, Corning). Filters were washed and centrifuged with BTPWB (500 μL, 14,000 rcf, 30 s, 10 ×) until the emulsion was broken and the filter cleared of oil. *NcoI*-HF digestion mix (100 μL, 0.2 U/μL) was added to the beads and incubated with rocking (1 h, 37 ºC). Digest was collected by placing the filter into a clean 1.5-mL tube and centrifuging (2690 g, 30 s). Hit bead DNA tags were amplified and sequencing barcodes and Illumina adapters (P5, P7) were installed in two separate PCRs. The first qPCR consisted of *Taq* DNA polymerase (0.05 U/μL), oligonucleotide primers 5′-CGCCAGGGTTTTCCCAGTCACGAC-3′ (0.2 μM) and 5′-GTGGCACAACAACTGGCGGGCAAAC-3′ (0.2 μM), and qPCR buffer (1X). Hit bead digest (1 μL) was added to individual wells with qPCR matrix (20 μL). Standard solutions (1 fmol, 100 amol, 10 amol, 1 amol, 100 zmol, 10 zmol, 1 zmol, each in 1 μL BTPWB) were added to separate wells with qPCR matrix (20 μL). Reactions were thermally cycled (95 °C, 2 min; [95 °C, 20 s; 68 °C, 30 s; 72 °C, 30 s] × 45 cycles) with fluorescence monitoring (SYBR channel, QuantStudio™ 3, ThermoFisher), and the reaction was stopped when amplification signal from wells containing hit digest reached maximum fluorescence. Wells with amplified hit sequences from the same screening day were pooled.

The second qPCR matrix contained forward primer 5′-AATGATACGGCGACCACC GAGATCTACACTCTTTCCCTACACGACGCTCTTCCGATCTGCCGCCGCCTTCGTC CTTCTCAGCGAC-3′ (0.2 μM). Hit collections corresponding to separate screening days were amplified with a reverse primer 5′-CAAGCAGAAGACGGCATACGAGATXXXX

XXGTGACTGGAGTTCAGACGTGTGCTCTTCCGATCTGTGGCACAACAACTGGCG GGCAAAC-3′ (0.2 µM), introducing a unique barcode (5′-XXXXXX-3′) to identify each screening set. The matrix also consisted of *Taq* DNA polymerase (0.05 U/µL) and qPCR buffer (1X). Hit tag amplicon (1 µL) was added to individual wells with qPCR matrix (50 µL). Amplified standard solutions (1 fmol, 100 amol, 10 amol, 1 amol, 100 zmol, 10 zmol, 1 zmol, 1 µL each) were added to separate wells with qPCR matrix (50 µL). Reactions were thermally cycled (95 °C, 2 min; [95 °C, 20 s; 68 °C, 30 s; 72 °C, 30 s] × 45 cycles) with fluorescence monitoring (SYBR channel, QuantStudio™ 3, ThermoFisher), and the reaction was stopped when amplification signal from wells reached maximum fluorescence. Wells with amplified hit sequences from the same screening day were pooled into clean 1.5 mL microtubes, and 1.8× volumes of 20% PEG binding buffer was added. Mag-Bio beads were added (0.2× sample volume) to each microtube. After incubation (5 m, RT), microtubes were placed on a magnet and supernatant was removed. Samples were washed twice with ice-cold 80% EtOH (500 µL) and allowed to dry (15 min, RT). Hit sequence amplicons were eluted with 25–50 µL 10 mM Tris, pH 7.5. Purified amplification product was resolved by native PAGE (6%, 19:1 acrylamide/bisacrylamide, 1X TBE, 150 V, 30 min) and visualized with SYBR Gold staining. Samples with 264-bp DNA product were sent for Illumina sequencing (MiSeq v2 Nano, paired end).

NGS Decoding

DNA sequences were aligned to a degenerate reference sequence (5′-GCCGCCGCCTTCG TCCTTCTCAGCGACATGGNNNNNNNNTCANNNNNNNNGTTNNNNNNNNCTA NNNNNNNNTTCNNNNNNNNCGCNNNNNNNNGCCTCCCAAACNNNNNNNN GTTTGCCCGCCAGTTGTTGTGCCAC–3′) using a mismatch score of -1 and an internal gap score of -10. The first 6 degenerate sequence positions (5′-NNNNNNNN-3′) define the DNA encoding regions, while the position 7 degenerate sequence (5′-NNNNNNNN-3′) contains the unique molecular identifier (UMI) to tag each DNA molecule. Encoding

region sequences were matched to an alpha numeric DNA region identifier (11xx, 22xx, 13xx, 24xx, 15xx, 26xx, respectively), while allowing a 1-bp mutation per encoding region. No insertions or deletions were tolerated. Sequences were discarded that (1) did not match a known encoding region identifier at all six encoding positions (2) contained a single point mutation in more than 4 of the DNA encoding regions (3) contained a UMI length <8 bp (4) did not contain an expected combination of encoding region identifiers presented in the library or positive control designs or (5) contained a minimum Phred quality score less than 20. A sum of UMI counts for all remaining unique combinations of encoding region identifiers was then determined using a UMI aggregation algorithm; 1 bp mutations were considered the same UMI. Encoding region identifiers were then converted to library BB identifiers per the library design with an associated UMI count. Any encoding region/ bead-specific barcode combinations with a UMI count below a manually determined threshold (10) were discarded. The remaining "valid beads" were aggregated by cycle 1/2 BB combinations and the number of unique encoding regions observed for each BB combination defined the *k*-class for that BB combination.[S8]

Microplate IVTT Assays

Wheat germ lysate IVTT plate assays were prepared as 5 µL reactions. Wheat germ lysate (3 µL) was added to test compound or vehicle control (1 µL) in a 384-well black microplate and the reaction was incubated (10 min, RT). Template (1 µL) was added and reaction progress was immediately monitored (λ_{ex} = 480 nm, λ_{em} = 530 nm, 27 °C, 2 h). Relative activity was calculated using the slope of fluorescence increase over time (at 15–30 min post reaction initiation) for compound samples relative to that of the DMSO vehicle control. All microplate experiments contained 25 ng/µL plasmid, except for assays with RPL27[1:32]-GFP and KRAS^{G12D}[1:189]-GFP, which contained 20 and 60 ng/µL plasmid, respectively. HeLa lysate IVTT plate assays were prepared as 5.5 µL reactions. HeLa lysate (2.5 µL) and accessory proteins (0.5 µL) were added to test compound or vehicle

control (1 µL) in a 384-well black microplate and the reaction was incubated (10 min, RT). Reaction buffer (1 µL) and template (0.5 µL) were added and reaction progress was immediately monitored (λ_{ex} = 480 nm, λ_{em} = 530 nm, 27 °C, 2 h). Relative activity was calculated using the slope of fluorescence increase over time (15–30 min post reaction initiation) for compound samples relative to that of the DMSO vehicle control. All microplate experiments contained 25 ng/µL plasmid, except for assays with RPL27[1:32]-GFP and KRAS^{G12D}[1:189]-GFP, which contained 20 and 60 ng/µL plasmid, respectively.

Cloning of Target Genes

All templates for WG IVTT were prepared in the pF3A plasmid, which contains *Sgf*I and *Pme*I restriction endonuclease recognition sites. Gene blocks were purchased from IDT consisting of the pF3A Kozak sequence, target gene, linker, and GFP reporter. *Sgf*I and *Pme*I sites flanked the insert with 6–8 nt spacers. Digestion with *Sgf*I and *Pme*I allowed for directional insertion of the gene block into the pF3A vector. Sequence-verified plasmids were cloned into DH5α *E. coli* for standard large-scale DNA plasmid preparation.

All templates for HeLa IVTT were prepared in the pT7CFE plasmid, which contains *Nde*I and *Xho*I restriction endonuclease recognition sites. Gene blocks were purchased from IDT consisting of the pT7CFE Kozak sequence, target gene, linker, and GFP reporter. *Nde*I and *Xho*I sites flanked the insert with 6–8 nt spacers. Digestion with *Nde*I and *Xho*I allowed for directional insertion of the gene block into the pT7CFE vector. Sequence-verified plasmids were cloned into DH5α *E. coli* for standard large-scale DNA plasmid preparation.

HepG2 Cellular Assay and Western Blot

HepG2 cells were seeded in a clear 12-well plate (2.2 x 10^5 cells/well) containing media (4.5 g/L high-glucose DMEM, 2 mM L-glutamine, 10% fetal bovine serum, 1% penicillin-

streptomycin, 1X NEAA mixture) and incubated (16 h, 37 ºC, 5% CO_2, and 95% humidity). Growth media was replaced with serum-free media with compound treatment or control (DMSO), and cells were incubated (16 h, 37 ºC, 5% CO_2, 95% humidity). Following treatment, cells were kept on ice and washed (3× TBS). Cells were lysed (100 µL RIPA buffer, 1X Turbo DNAse). Protein lysates (24 µg) were separated by denaturing polyacrylamide gel electrophoresis (4-20% mini-PROTEAN TGX gel, 150 V) and transferred to a nitrocellulose membrane (0.2 µm) using a Trans-Blot Turbo Transfer System (Bio-Rad). The membrane was blocked (5% nonfat milk in PBS-T, 1 h) then incubated with primary antibodies overnight (1:6000 GAPDH direct conjugate antibody, 5% BSA in PBS-T; 1:1000 PCSK9 rabbit monoclonal antibody, 5% BSA in PBS-T). The PCSK9 probed membrane was then washed (3 × PBS-T) and probed with secondary detection antibody (1:2000 HRP-linked anti-rabbit, 5% BSA in PBS-T, 1 h, RT). HRP-labeled bands were visualized using ECL Western blot detection reagents.

Coding Oligonucleotides

xx	5' to 3'			
	x1xx, x3xx, x5xx		x2xx, x4xx, x6xx	
	11xx[+]	11xx[-]	22xx[+]	22xx[-]
1	TGGAAAGT	ACTTTCCA	CCTCCTAA	TTAGGAGG
2	ACGGAGCA	TGCTCCGT	AACCTCAA	TTGAGGTT
3	TTGGAGTT	AACTCCAA	AATCCCAT	ATGGGATT
4	AAGGAGGT	ACCTCCTT	AACCCTAC	GTAGGGTT
5	AGAAAGCA	TGCTTTCT	ATCCTCTC	GAGAGGAT
6	ACAGAACT	AGTTCTGT	CATTTCAA	TTGAAATG
7	TAAGGAGT	ACTCCTTA	CGCCTTCA	TGAAGGCG
8	ATGGGAGT	ACTCCCAT	CGTTCCTG	CAGGAACG
9	TGAAGGAA	TTCCTTCA	TTCTTCAT	ATGAAGAA
10	TTGAGGAT	ATCCTCAA	TCCTCTTA	TAAGAGGA
11	AAAGAAAC	GTTTCTTT	AACCTTCG	CGAAGGTT
12	AAAGGACA	TGTCCTTT	AACTCCCG	CGGGAGTT
13	AAAGGGAT	ATCCCTTT	AACTCTTT	AAAGAGTT
14	AAGAAACC	GGTTTCTT	AATCCTCA	TGAGGATT
15	AAGAGGCA	TGCCTCTT	AATCTCCC	GGGAGATT
16	ACAAAGAC	GTCTTTGT	AATCTTGT	ACAAGATT
17	ACAAGAAA	TTTCTTGT	AATTCCGA	TCGGAATT
18	ACAAGGCT	AGCCTTGT	ACCCTCCT	AGGAGGGT
19	ACAGGGTA	TACCCTGT	ACCCTTGA	TCAAGGGT
20	ACGAAAGA	TCTTTCGT	ACCTCCAA	TTGGAGGT
21	ACGAGATT	AATCTCGT	ACCTCTCC	GGAGAGGT
22	ACGAGGGC	GCCCTCGT	ACCTTCGC	GCGAAGGT
23	ACGGAATC	GATTCCGT	ACTCCCGC	GCGGGAGT
24	ACGGGAAG	CTTCCCGT	ACTCCTTT	AAAGGAGT
25	AGAAGACC	GGTCTTCT	ACTTCCCT	AGGGAAGT
26	AGGAAGGG	CCCTTCCT	AGCCCTCT	AGAGGGCT
27	AGGGAAAT	ATTTCCCT	AGCTCCTC	GAGGAGCT
28	ATAAGGGA	TCCCTTAT	AGTCTCTA	TAGAGACT
29	ATAGAGCC	GGCTCTAT	AGTTCTGT	ACAGAACT
30	CAAAGACT	AGTCTTTG	AGTTTCAT	ATGAAACT
31	CAAAGGAC	GTCCTTTG	ATCCCTTA	TAAGGGAT
32	CAAGAAGA	TCTTCTTG	ATCTCCGT	ACGGAGAT
33	CAAGAGTC	GACTCTTG	ATCTTCCA	TGGAAGAT
34	CAGAAGGA	TCCTTCTG	ATTCTCGG	CCGAGAAT
35	CAGAGAAA	TTTCTCTG	ATTTCCAC	GTGGAAAT
36	CAGGGACG	CGTCCCTG	CACCCTGA	TCAGGGTG
37	CCGAAACT	AGTTTCGG	CACCTCGC	GCGAGGTG
38	CCGAGGAG	CTCCTCGG	CACCTTAT	ATAAGGTG
39	CCGGAGGG	CCCTCCGG	CACTCCAT	ATGGAGTG
40	CGAGAACC	GGTTCTCG	CATCCCTA	TAGGGATG
41	CGAGGAGG	CCTCCTCG	CCCTCCGG	CCGGAGGG
42	CGAGGGCA	TGCCCTCG	CCCTTCTA	TAGAAGGG
43	CGGGAATA	TATTCCCG	CCCTTTCG	CGAAAGGG
44	CGGGAGCT	AGCTCCCG	CCTCTCAT	ATGAGAGG
45	CTGAAGCC	GGCTTCAG	CCTTTCCC	GGGAAAGG
46	CTGGAAAC	GTTTCCAG	CGCTCCCA	TGGGAGCG
47	GAGAGGGT	ACCCTCTC	CGTCCCAC	GTGGGACG
48	GAGGAACA	TGTTCCTC	CGTCCTGG	CCAGGACG
49	GAGGGAAT	ATTCCCTC	CGTCTCCG	CGGAGACG
50	GCAAAGGG	CCCTTTGC	CTCCCTCG	CGAGGGAG
51	GCAGAGAA	TTCTCTGC	CTTCCCGT	ACGGGAAG
52	GCAGGACC	GGTCCTGC	GACTCCGC	GCGGAGTC
53	GCGGAAGT	ACTTCCGC	GCCCTCGG	CCGAGGGC
54	GCGGGATA	TATCCCGC	GCCCTTCC	GGAAGGGC
55	GGAAGAGA	TCTCTTCC	GCCTCCTT	AAGGAGGC
56	GGAGAGGT	ACCTCTCC	GCTCCCTG	CAGGGAGC
57	GGAGGATT	AATCCTCC	GGCCCTAA	TTAGGGCC
58	GGAGGGAC	GTCCCTCC	GGCTCTCG	CGAGAGCC
59	GGGAAACG	CGTTTCCC	GGCTTCCC	GGGAAGCC
60	GGGAAGAA	TTCTTCCC	GGTCCCGA	TCGGGACC
61	GGGAGGTC	GACCTCCC	GGTTTCGG	CCGAAACC
62	GTAAAGTC	GACTTTAC	GTTTCCCG	CGGGAAAC
63	GTAGAAGC	GCTTCTAC	TACCTCTT	AAGAGGTA
64	GTAGGAAA	TTTCCTAC	TACTTCGA	TCGAAGTA
65	GTAGGGCT	AGCCCTAC	TCCCTCAC	GTGAGGGA
66	GTGGAGGA	TCCTCCAC	TCCTTTGT	ACAAAGGA
67	TAAGAGCA	TGCTCTTA	TCTCCTCC	GGAGGAGA
68	TCAAAGTT	AACTTTGA	TCTTCCTC	GAGGAAGA
69	TCGGAGAC	GTCTCCGA	TGTCCCTT	AAGGGACA
70	TCGGGACT	AGTCCCGA	TGTCTTCT	AGAAGACA
71	TTAGGGTC	GACCCTAA	TGTTCTAA	TTAGAACA
72	TTGAGAGA	TCTCTCAA	TTCCCTAT	ATAGGGAA

Compound Synthesis and Characterization

Synthesis of (R)-4-(4-((3-(1-(((9H-fluoren-9-yl)methoxy)carbonyl)-N-(8-methylisoquinolin-1-yl)piperidine-4-carboxamido)piperidin-1-yl)methyl)-2-methoxy-5-nitrophenoxy)butanoic acid (Compound 1).

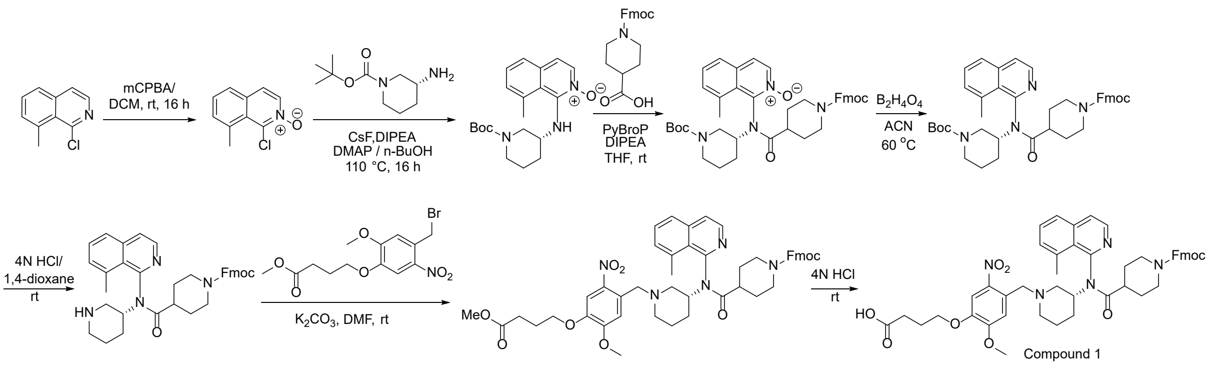

Step 1: To a stirred solution of 1-chloro-8-methylisoquinoline (11 g, 62.1 mmol) in DCM (200 mL) was added mCPBA (34.8 g, 201.7 mmol) portion-wise at 0 °C, and then the reaction was stirred at room temperature for 16 h. The reaction mixture was basified with aqueous sodium bicarbonate (150 mL), and then the product was extracted with DCM (2 x 300 mL). The combined organic layers were dried over Na_2SO_4 and concentrated. The product was purified by Combi-Flash, eluting with ethyl acetate to afford 1-chloro-8-methylisoquinoline 2-oxide (3 g, 15.5 mmol) as a brown solid.

Step 2: To a stirred solution of 1-chloro-8-methylisoquinoline 2-oxide (22.2 g, 115 mmol) and tert-butyl(R)-3-aminopiperidine-1-carboxylate (37.2 g, 186 mmol) in n-butanol (150 mL) were added cesium fluoride (18.8 g, 124 mmol), DMAP (1.5 g, 12.4 mmol), and DIEA (44 ml, 248 mmol) at room temperature. The reaction was then stirred at 110 °C for 16 h, after which the reaction was diluted with water (300 mL). The product was extracted with ethyl acetate (3 x 500 mL), and the combined organic layers were dried over Na_2SO_4 and concentrated. The product was purified by silica gel (60-120 mesh)

using 4% MeOH in DCM to afford (R)-1-((1-(tert-butoxycarbonyl)piperidin-3-yl)amino)-8-methylisoquinoline 2-oxide (22 g, impure) as brown solid.

Step 3: To a stirred solution of (R)-1-((1-(tert-butoxycarbonyl)piperidin-3-yl)amino)-8-methylisoquinoline 2-oxide (380 mg, 1.1 mmol) and 1-(((9H-fluoren-9-yl) methoxy) carbonyl) piperidine-4-carboxylic acid (373 mg, 1.1 mmol) in THF (12 mL) were added DIEA (0.6 mL, 3.2 mmol) and then PyBroP (743 mg, 1.6 mmol) at 0 °C. The reaction was stirred at room temperature for 2 h. The reaction mixture was then diluted with water (60 mL) and extracted with ethyl acetate (2 x 80 mL). The organic layers were washed with saturated NaCl (20 mL), dried over Na_2SO_4, and concentrated. The product was purified by Combi-Flash, eluting with ethyl acetate to afford (R)-1-(1-(((9H-fluoren-9-yl)methoxy) carbonyl)-N-(1-(tert-butoxycarbonyl)piperidin-3-yl)piperidine-4-carboxamido)-8-methylisoquinoline 2-oxide (300 mg, 0.43 mmol) as an off-white solid. LC-MS: [M+H$^+$] = 691.4.

Step 4: To a stirred solution of (R)-1-(1-(((9H-fluoren-9-yl)methoxy)carbonyl)-N-(1-(tert-butoxycarbonyl)piperidin-3-yl)piperidine-4-carboxamido)-8-methylisoquinoline 2-oxide (300 mg, 0.43 mmol) in ACN (15 mL) was added hypodiboric acid (120 mg, 1.34 mmol) at room temperature, and the reaction was stirred at 60 °C for 2 h. The reaction was then diluted with EtOAc (20 mL) and washed with water (40 mL). The organic layer was washed with saturated NaCl (20 mL), dried over Na_2SO_4, and concentrated. The product was purified by Combi-Flash, eluting with 35% EtOAc to afford tert-butyl (R)-3-(1-(((9H-fluoren-9-yl)methoxy)carbonyl)-N-(8-methylisoquinolin-1-yl)piperidine-4-carboxamido)piperidine-1-carboxylate (170 mg, 0.25 mmol) as an off-white solid. LC-MS: [M+H$^+$] = 675.4.

Step 5: To a stirred solution of tert-butyl (R)-3-(1-(((9H-fluoren-9-yl)methoxy)carbonyl)-N-(8-methylisoquinolin-1-yl)piperidine-4-carboxamido)piperidine-1-carboxylate (170 mg, 0.25 mmol) was added 4M HCl in 1,4-dioxane (3 mL), and the reaction was stirred for 2 h at room temperature. The reaction was then concentrated to afford (9H-fluoren-9-yl)methyl (R)-4-((8-methylisoquinolin-1-yl)(piperidin-3-yl)carbamoyl)piperidine-1-

carboxylate (150 mg, crude) as a light yellow solid. LC-MS: [M+H$^+$] = 575.3.

Step 6: To a stirred solution of (9H-fluoren-9-yl)methyl (R)-4-((8-methylisoquinolin-1-yl)(piperidin-3-yl)carbamoyl)piperidine-1-carboxylate (150 mg, 0.26 mmol) in DMF (8 mL) were added K_2CO_3 (108 mg, 0.78 mmol) and methyl 4-(4-(bromomethyl)-2-methoxy-5-nitrophenoxy)butanoate (94 mg, 0.26 mmol) at 0 °C. The reaction was stirred at room temperature for 4 h. The reaction mixture was poured into ice cold water and a solid precipitated out. The isolated solid was filtered, washed with water, and dried. The product was purified by Prep HPLC (Conditions: Column: X-BRIDGE PREP OBD C18 250mm x 19 mm, 5µm, Mobile phase A: 10mM NH_4OAc in water, Mobile phase B: 100 % acetonitrile, Gradient: T/%B: 0/65, 10/95, Flow: 10mL/min). The product was dried to afford (9H-fluoren-9-yl)methyl (R)-4-((1-(5-methoxy-4-(4-methoxy-4-oxobutoxy)-2-nitrobenzyl)piperidin-3-yl)(8-methylisoquinolin-1-yl)carbamoyl)piperidine-1-carboxylate (80 mg, 0.09 mmol) as an off-white solid. LC-MS: [M+H$^+$] = 856.4.

Step 7: To a stirred solution of methyl (9H-fluoren-9-yl)methyl (R)-4-((1-(5-methoxy-4-(4-methoxy-4-oxobutoxy)-2-nitrobenzyl)piperidin-3-yl)(8-methylisoquinolin-1-yl)carbamoyl) piperidine-1-carboxylate (80 mg, 0.09 mmol) in THF (2 mL) at 0 °C was added 4N HCl (2 mL). The reaction was stirred at room temperature for 16 h. The reaction was concentrated to afford Compound 1 (80 mg, 0.08 mmol) as a pale yellow solid. LC-MS: [M+H$^+$] = 842.95.

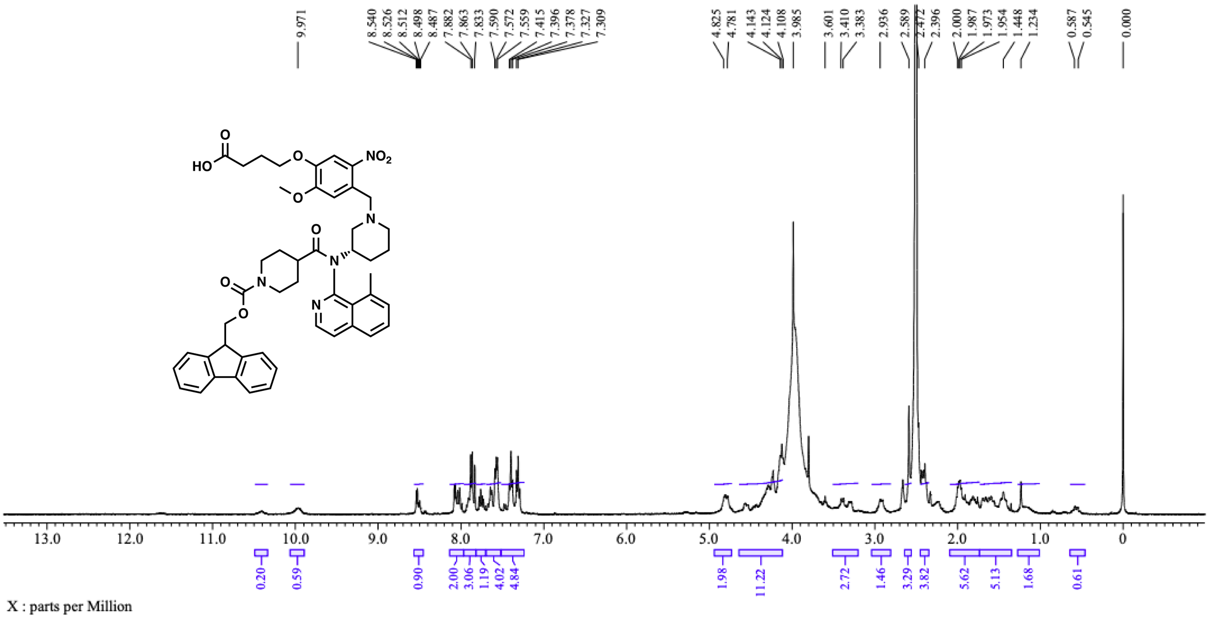

^{1}H NMR (DMSO-d_6, 400 MHz) δ 8.54-8.49 (m, 1H), 8.08-7.99 (m, 2H), 7.90-7.83 (m, 3H), 7.78-7.73 (m, 1H), 7.67-7.57 (m, 4H), 7.41-7.29 (m, 4H), 4.83-4.81 (m, 1H), 4.35-4.12 (m, 5H), 3.98 (s, 2H), 3.87-3.78 (m, 2H), 3.65 (s, 3H), 3.33-3.30 (m, 2H), 2.68-2.66 (m, 4H), 2.61 (s, 3H), 2.43-2.24 (m, 3H), 1.98-1.82 (m, 5H), 1.70-1.59 (m, 3H), 1.48-1.45 (m, 2H).

Synthesis of (4-(((R)-3-((S)-1-(((9H-fluoren-9-yl)methoxy)carbonyl)-N-(8-methylisoquinolin-1-yl)piperidine-3-carboxamido) piperidin-1-yl)methyl)-2-methoxy-5-nitrophenoxy)butanoic acid (Compound 2)

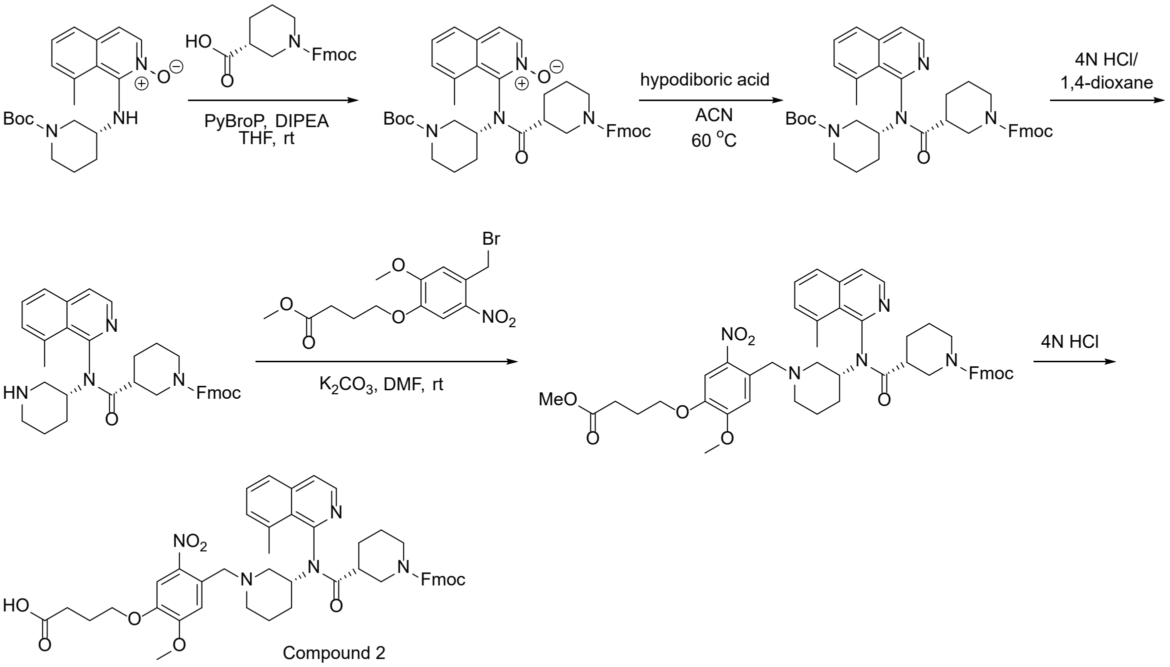

Compound 2

Step 1: To a stirred solution of (R)-1-((1-(tert-butoxycarbonyl)piperidin-3-yl)amino)-8-methylisoquinoline 2-oxide (350 mg, 0.97 mmol) and (R)-1-(((9H-fluoren-9-yl)methoxy) carbonyl)piperidine-3-carboxylic acid (344 mg, 0.97 mmol) in THF (12 mL) at 0 °C were added DIEA (0.54 mL, 2.93 mmol) and then PyBroP (685 mg, 1.46 mmol). The reaction was stirred at room temperature for 30 min. The reaction mixture was diluted with water (60 mL) and extracted with ethyl acetate (2 x 80 mL). The combined organic layers were washed with ice cold water, dried over Na_2SO_4, filtered, and concentrated. The residue was purified by Combi-Flash, eluting with 60% EtOAc to afford 1-((R)-1-(((9H-fluoren-9-yl) methoxy) carbonyl)-N-((R)-1-(tert-butoxy carbonyl) piperidin-3-yl) piperidine-3-carboxamido)-8-methylisoquinoline-2-oxide (340 mg, 0.49 mmol) as an off-white solid. LC-MS: $[M+H^+]$ = 691.3.

Step 2: To a stirred solution of 1-((R)-1-(((9H-fluoren-9-yl)methoxy)carbonyl)-N-((R)-1-(tert-butoxycarbonyl)piperidin-3-yl)piperidine-3-carboxamido)-8-methylisoquinoline-2-oxide (340 mg, 0.49 mmol) in ACN (6 mL) at room temperature was added hypodiboric acid (132 mg, 1.47 mmol), and then the reaction mixture was stirred at 60 °C for 1 h. The reaction mixture was diluted with EtOAc (20 mL) and washed with water (40 mL) and brine (20 mL). The organic layer was dried over Na_2SO_4 and concentrated. The residue was purified by Combi-Flash, eluting with 50% EtOAc in hexane, followed by prep HPLC (Conditions: Chiral Pak IA (250 x 20 x 5μm), Mobile phase A: 0.1 % DEA in MTBE, Mobile phase B: ethanol (100%), Gradient (T/%B): 0/ 75/25, Flow: 15 mL/min.) to afford (9H-fluoren-9-yl)methyl (R)-3-(((R)-1-(tert-butoxycarbonyl)piperidin-3-yl)(8-methylisoquinolin-1-yl)carbamoyl)piperidine-1-carboxylate) (100 mg, 0.14 mmol) as a white solid. LC-MS: $[M+H^+]$ = 675.25.

Step 3: (9H-fluoren-9-yl)methyl (R)-3-(((R)-1-(tert-butoxycarbonyl)piperidin-3-yl)(8-methylisoquinolin-1-yl)carbamoyl)piperidine-1-carboxylate (100 mg, 0.14 mmol) was dissolved in 4M HCl in 1,4-dioxane (2 mL) at 0 °C, and the reaction was stirred at room temperature for 2 h. The reaction mixture was concentrated and the obtained compound was washed with n-pentene to afford ((9H-fluoren-9-yl)methyl (R)-3-((8-methylisoquinolin-1-yl)((R)-piperidin-3-yl)carbamoyl)piperidine-1-carboxylate (80 mg, 0.13 mmol) as an off-white solid. LC-MS: $[M+H^+]$ = 576.

Step 4: To a stirred solution of (9H-fluoren-9-yl)methyl(R)-3-((8-methylisoquinolin1-yl)((R)-piperidin-3yl)carbamoyl)piperidine-1-carboxylate (80 mg, 0.13 mmol) in DMF (6 mL) at 0 °C were added K_2CO_3 (57 mg, 0.41 mmol) and methyl 4-(4-(bromomethyl)-2-methoxy-5-nitrophenoxy)butanoate (50 mg, 0.13 mmol). The reaction was then stirred at room temperature for 2 h. The reaction mixture was poured into ice cold water, and a solid precipitated out. The solid was isolated by filtration and purified by Combi-Flash, eluting with 50% EtOAc in hexane to afford (9H-fluoren-9-yl)methyl(R)-3-(((R)-1-(5-methoxy-4-(4-methoxy-4-oxobutoxy)-2 nitrobenzyl)piperidin-3-yl)(8-methylisoquinolin-1-yl)

carbamoyl)piperidine-1-carboxylate (100 mg, 0.12 mmol) as an off-white solid LC-MS: [M+H$^+$] = 856.70.

Step 5: To a stirred solution of (9H-fluoren-9-yl)methyl (R)-3-(((R)-1-(5-methoxy-4-(4-methoxy-4-oxobutoxy)-2-nitrobenzyl)piperidin-3-yl)(8-methylisoquinolin-1yl)carbamoyl) piperidine-1-carboxylate (100 mg, 0.11 mmol) in THF (2 mL) at 0 °C was added 4 N HCl (2 mL). The reaction was then stirred at room temperature for 16 h, after which the reaction was concentrated. The product was washed with ethyl acetate (5 mL) and dried to afford Compound 2 (80 mg, 0.10 mmol) as a pale yellow solid. LC-MS: [M+H$^+$] = 842.65.

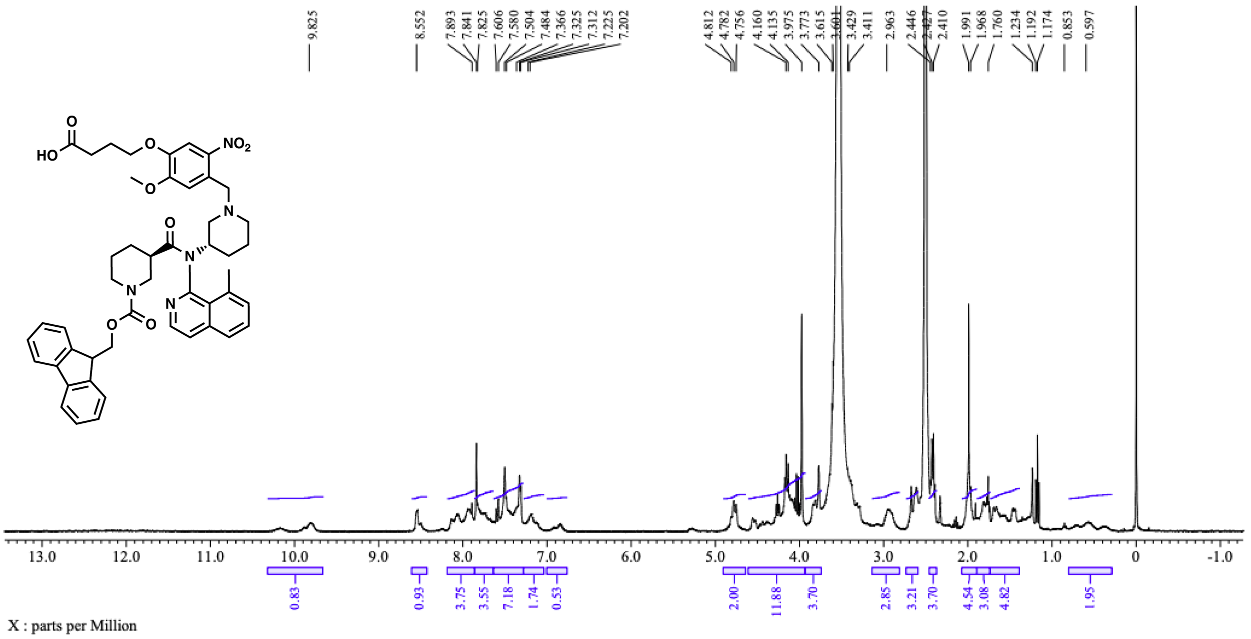

^{1}H-NMR (400 MHz, DMSO-d$_6$) δ 9.83 (m, 1H, J=12.0 Hz); 8.55 (s, 1H); 8.83-7.89 (m, 3H); 7.58-7.61 (d, 2H); 7.49 (s, 1H); 7.20-7.37 (m, 11H); 7.75-4.81 (t, 4H); 4.14-4.16 (m, 3H); 3.98 (s, 3H); 3.77 (s, 1H); 2.96 (m, 5H, J=12 Hz); 1.98 (s, 3H); 1.86 (m, 1H); 1.17-1.23 (m, 4H); 0.60-0.85 (m, 8H)

Synthesis of (R)-4-(4-((3-(4-(((9H-fluoren-9-yl)methoxy)carbonyl)-N-(1-methyl-1H-pyrrolo[2,3-c]pyridin-7-yl)piperazine-1-carboxamido) piperidin-1-yl) methyl)-2-methoxy-5-nitrophenoxy)butanoic acid (Compound 3)

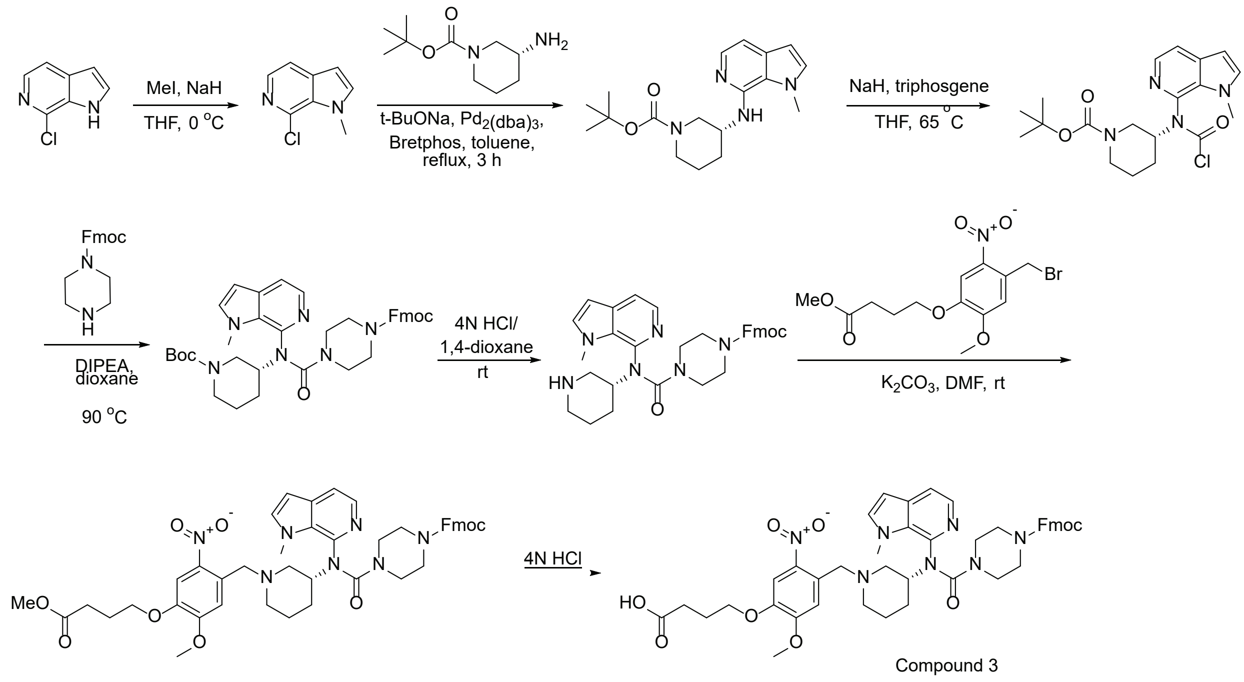

Step 1: To a stirred solution of 7-chloro-1H-pyrrolo[2,3-c] pyridine (15 g, 98.3 mmol) in THF (150 mL) at 0 °C was added NaH (4.71 g, 117.9 mmol) portion-wise, and the reaction was stirred at 0 °C for 30 min. Then methyl iodide (13.4 ml, 216.2 mmol) was added to the reaction dropwise at 0 °C. The reaction was stirred at 0 °C for 1 h and then at room temperature for 2 h. The reaction mixture was diluted with water (300 mL), and the product was extracted with EtOAc (2 x 500 mL). The combined organic layers were dried over Na_2SO_4 and concentrated. The product was purified by silica gel (60-120 mesh) using 20% EtOAc in hexane to afford 7-chloro-1-methyl-1H-pyrrolo[2,3-c] pyridine (14 g, 84 mmol) as an off-white solid. LC-MS: $[M+H^+]$ = 166.90.

Step 2: To a stirred solution of 7-chloro-1-methyl-1H-pyrrolo[2,3-c]pyridine (16 g, 96.0 mmol) and tert-butyl(R)-3-aminopiperidine-1-carboxylate (23 g, 115.3 mmol) in toluene

(150 mL) at room temperature were added sodium tert-butoxide (18.4 g, 192.1 mmol), Bretphos (10.3 g, 19.2 mmol), and $Pd_2(dba)_3$ (8.79 g, 9.60 mmol). The reaction mixture was stirred at 110 °C for 3 h. The reaction mixture was diluted with water (300 mL), and the product was extracted with ethyl acetate (3 x 500 mL). The combined organic layers were dried over Na_2SO_4 and concentrated. The product was purified by silica gel (60-120 mesh) using 50% EtOAc in hexane to afford tert-butyl (R)-3-((1-methyl-1H-pyrrolo[2,3-c] pyridin-7-yl) amino)piperidine-1-carboxylate (23 g, 69.6 mmol) as a brown solid. LC-MS: $[M+H^+]$ = 331.25.

Step 3: To a stirred solution of tert-butyl (R)-3-((1-methyl-1H-pyrrolo[2,3-c] pyridin-7-yl) amino) piperidine-1-carboxylate (0.5 g, 1.51 mmol) in THF (10 mL) at room temperature was added NaH (84 mg, 2.11 mmol), and the reaction was stirred at room temperature for 30 min. Then, triphosgene (314 mg, 1.05 mmol) in toluene (1 mL) was added to the reaction, and the reaction mixture was stirred at 60 °C for 1 h. The reaction mixture was diluted with water (50 mL), and the product was extracted with ethyl acetate (2 x 100 mL). The combined organic layers were dried over Na_2SO_4 and concentrated. The product was purified by Combi-Flash, eluting with 20% ethyl acetate in hexane to afford tert-butyl (R)-3-((chlorocarbonyl)(1-methyl-1H-pyrrolo[2,3-c]pyridin-7-yl)amino)piperidine-1-carboxylate (282 mg, 0.72 mmol) as a yellow liquid. LC-MS: $[M+H^+]$ = 393.10.

Step 4: To a solution of (9H-fluoren-9-yl)methyl piperazine-1-carboxylate (263 mg, 0.76 mmol) in 1,4-dioxane (20 mL) were added DIEA (0.7 mL, 3.81 mmol) and tert-butyl (R)-3-((chlorocarbonyl)(1-methyl-1Hpyrrolo[2,3-c]pyridin-7-yl)amino)piperidine-1-carboxylate (250 mg, 0.63 mmol). The reaction mixture was stirred at 90 °C for 3 h. The reaction was then diluted with water and extracted with EtOAc (2 x 80 mL). The combined organic layers were dried over Na_2SO_4 and concentrated. The product was purified by Combi-Flash, eluting with 50% EtOAc in hexane to afford (9H-fluoren-9-yl)methyl (R)-4-((1-(tert-butoxycarbonyl)piperidin-3-yl)(1-methyl-1H-pyrrolo[2,3-c]pyridin-7-yl)carbamoyl)

piperazine-1-carboxylate (270 mg, 0.40 mmol) as an off-white solid. LC-MS: [$M+H^+$] = 665.25.

Step 5: To a solution of (9H-fluoren-9-yl)methyl (R)-4-((1-(tert-butoxycarbonyl)piperidin-3-yl)(1-methyl-1H-pyrrolo[2,3- c]pyridin-7-yl)carbamoyl)piperazine-1-carboxylate (230 mg, 0.34 mmol) in DCM (2 mL) at 0 °C was added 4M HCl in 1,4-dioxane (4 mL). The reaction was stirred at room temperature for 2 h, after which it was concentrated to afford (9H-fluoren-9-yl)methyl (R)-4-((1-methyl-1H-pyrrolo[2,3-c]pyridin-7-yl)(piperidin-3-yl) carbamoyl)piperazine-1-carboxylate (200 mg, 0.33 mmol) as an off-white solid. LC-MS: [$M+H^+$] = 565.65.

Step 6: To a stirred solution of (9H-fluoren-9-yl)methyl (R)-4-((1-methyl-1H-pyrrolo[2,3-c]pyridin-7-yl)(piperidin-3- yl)carbamoyl)piperazine-1-carboxylate (200 mg, 0.35 mmol) in DMF (10 mL) at 0 °C were added K_2CO_3 (146 mg, 1.06 mmol) and methyl 4-(4-(bromomethyl)-2-methoxy-5-nitrophenoxy)butanoate (127 mg, 0.35 mmol). The reaction was stirred at room temperature for 2 h, after which the reaction mixture was poured into ice cold water, and the resulting solid was filtered. The product was purified by Combi-Flash, eluting with 80% EtOAc in hexane to afford (9H-fluoren-9-yl)methyl (R)-4-((1-(5-methoxy-4-(4-methoxy-4-oxobutoxy)-2-nitrobenzyl)piperidin-3-yl)(1-methyl-1H-pyrrolo[2,3-c]pyridin-7-yl)carbamoyl)piperazine-1-carboxylate (150 mg, 0.17 mmol) as an off-white solid. LC-MS: [$M+H^+$] = 846.7.

Step 7: To a solution of (9H-fluoren-9-yl)methyl (R)-4-((1-(5-methoxy-4-(4-methoxy-4-oxobutoxy)-2- nitrobenzyl)piperidin-3-yl)(1-methyl-1H-pyrrolo[2,3-c]pyridin-7-yl) carbamoyl)piperazine-1-carboxylate (150 mg, 0.17 mmol) in THF (3 mL) at 0 °C was added 4N HCl (3 mL). The reaction was stirred at room temperature for 16 h and then concentrated. The obtained compound was washed with ethyl acetate and dried to afford Compound 3 (145 mg, 0.17 mmol) as a pale yellow solid. LC-MS: [$M+H^+$] = 832.65.

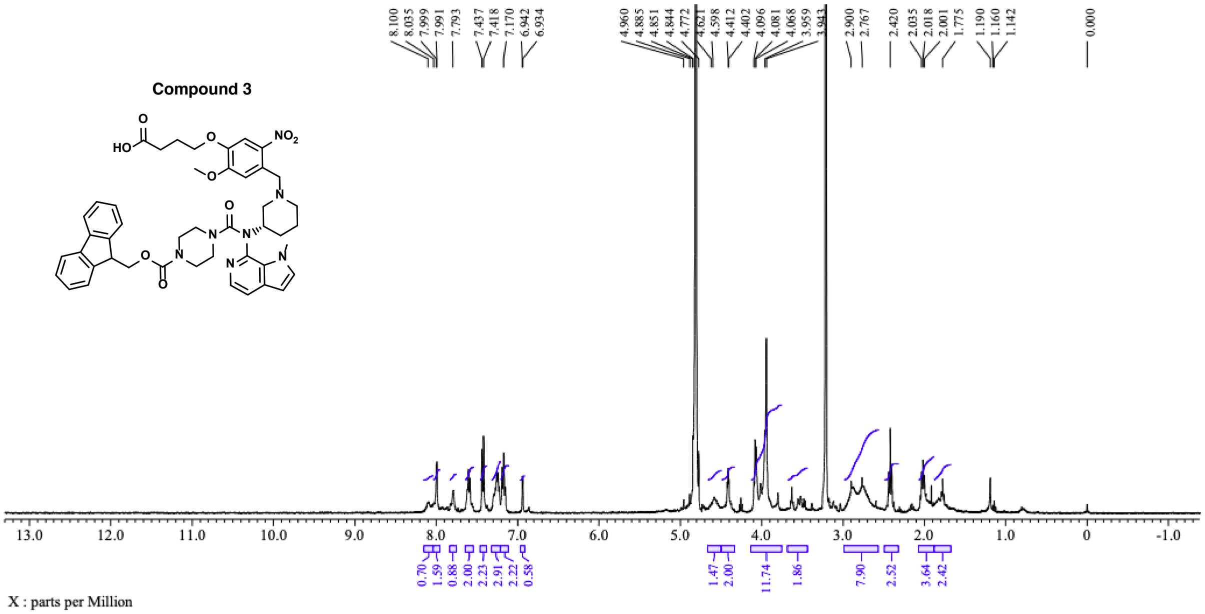

^{1}H NMR (CD_3OD, 400 MHz) δ 8.10-7.99 (m, 3H), 7.82-7.79 (m, 1H), 7.61-7.58 (m, 2H), 7.42 (d, J=7.6 Hz, 2H), 7.29-7.22 (m, 3H), 7.19-7.15 (m, 2H), 6.94-6.93 (m, 1H), 4.62-4.59 (m, 2H), 4.42-4.40 (m, 2H), 4.08-3.93 (m, 12H), 3.63-3.28 (m, 2H), 2.91-2.60 (m, 8H), 2.43-2.37 (m, 3H), 2.05-1.92 (m, 4H), 1.79-1.76 (m, 2H).

Synthesis of Tert-butyl (R)-3-(N-(8-methylisoquinolin-1-yl)piperidine-4-carboxamido) piperidine-1-carboxylate (Intermediate 1)

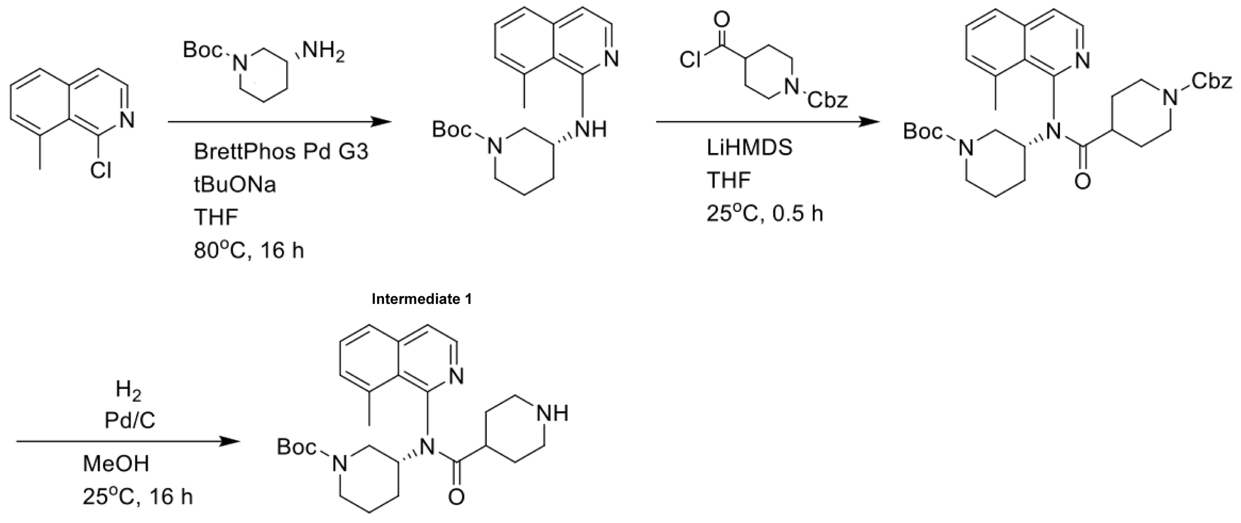

Step 1: To a solution of tert-butyl (R)-3-aminopiperidine-1-carboxylate (8.29 g, 41.3 mmol) and 1-chloro-8-methylisoquinoline (4.90 g, 27.59 mmol) in THF (200 mL) were added BrettPhos Pd G3 (750 mg, 827 µmol) and tBuONa (7.95 g, 82.7 mmol). The mixture was stirred at 80 °C for 16 h under N_2. The reaction mixture was concentrated to give a residue that was purified by flash silica gel chromatography (ISCO®; 80 g SepaFlash® Silica Flash Column, eluent of 0-10% ethyl acetate/petroleum ether gradient @ 100 mL/min) to afford tert-butyl (R)-3-((8-methylisoquinolin- 1-yl)amino)piperidine-1-carboxylate (8.00 g, 23.4 mmol yield) as a green oil. LC-MS: [M+H^+] = 342.1

Step 2: To a solution of tert-butyl (R)-3-((8-methylisoquinolin-1-yl)amino)piperidine-1-carboxylate (3.00 g, 8.79 mmol) in THF (60 mL) was added LiHMDS (1 M, 14.1 mL) under N_2. The mixture was stirred at 25 °C for 15 min under N_2. Then benzyl 4-(chlorocarbonyl) piperidine-1-carboxylate (3.20 g, 11.4 mmol) was added to the reaction mixture. The reaction was stirred at 25 °C for 15 min under N_2. The reaction mixture was quenched by adding saturated NH_4Cl (300 mL), and the product was extracted with ethyl acetate (3 x 150 mL). The combined organic layers were washed with brine (300 mL), dried over $MgSO_4$, filtered, and concentrated to give a residue that was purified by silica gel

(ISCO®; 20 g SepaFlash® Silica Flash Column, Eluent of 0 15% THF/PE @ 100 mL/min) to afford tert-butyl (R)-3-(1-((benzyloxy)carbonyl)-N-(8-methylisoquinolin-1- yl)piperidine-4-carboxamido)piperidine-1-carboxylate (2.26 g, 3.85 mmol) as a yellow solid. LC-MS: [M+H$^+$] = 587.3

Step 3: To a solution of tert-butyl (R)-3-(1-((benzyloxy)carbonyl)-N-(8-methylisoquinolin-1-yl)piperidine-4- carboxamido) piperidine-1-carboxylate (2.86 g, 4.87 mmol) in MeOH (60 mL) was added Pd/C (0.286 g, 269 µmol, 10% purity) under N_2. The suspension was degassed and purged with H_2 3 times. The mixture was stirred under H_2 (50 psi) atmosphere at 25 °C for 16 h. The reaction mixture was filtered through celite and concentrated. The crude product was triturated with 30 mL n-pentane at 25 °C for 180 min. Intermediate 1 (1.60 g, 4.69 mmol) was obtained as a yellow solid. LC-MS: [M+H$^+$] = 453.2

Synthesis of (R)-1-(3-cyano-1H-indole-7-carbonyl)-N-(8-methylisoquinolin-1-yl)-N-(piperidin-3-yl)piperidine-4-carboxamide (Compound 9)

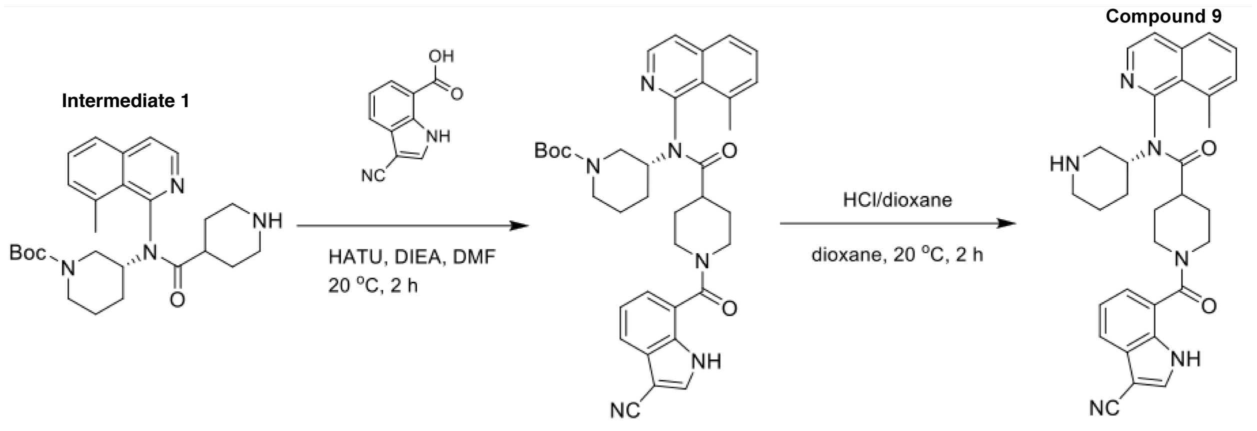

Step 1: To a solution of tert-butyl (R)-3-(N-(8-methylisoquinolin-1-yl)piperidine-4-carboxamido) piperidine-1-carboxylate (500 mg, 1.10 mmol) and 3-cyano-1H-indole-7-carboxylic acid (226 mg, 1.22 mmol) in DMF (5 mL) were added HATU (630 mg, 1.66 mmol) and DIEA (428 mg, 3.31 mmol, 577 µL). The mixture was stirred at 20 °C for 2 h, after which the reaction was poured into water (20 mL). The aqueous phase was extracted

with ethyl acetate (3 x 10 mL), and the combined organic phases were washed with brine (2 x 10 mL), dried with anhydrous Na_2SO_4, filtered, and concentrated. The crude product was used in the next step directly without purification. Tert-butyl (R)-3-(1-(3-cyano-1H-indole-7-carbonyl)-N-(8-methylisoquinolin-1-yl)piperidine-4-carboxamido) piperidine-1-carboxylate (0.30 g, 0.48 mmol) was obtained as a yellow oil. LC-MS: [M+H$^+$] = 621.3

Step 2: To a solution of tert-butyl (R)-3-(1-(3-cyano-1H-indole-7-carbonyl)-N-(8-methyl-isoquinolin-1-yl)piperidine-4-carboxamido) piperidine-1-carboxylate (0.30 g, 483 µmol) in dioxane (2 mL) was added HCl/dioxane (4 M, 3 mL). The mixture was stirred at 20 °C for 2 h and then concentrated. The residue was purified by preparative HPLC (column: Welch Xtimate C18 40 x 200 mm 7 µm; mobile phase: [water (NH_3H_2O+NH_4HCO_3)-ACN]; gradient: 20%-60% B over 25 min). Compound 9 (22.5 mg, 43 µmol) was obtained as a yellow solid. LC-MS: [M+H$^+$] = 521.3

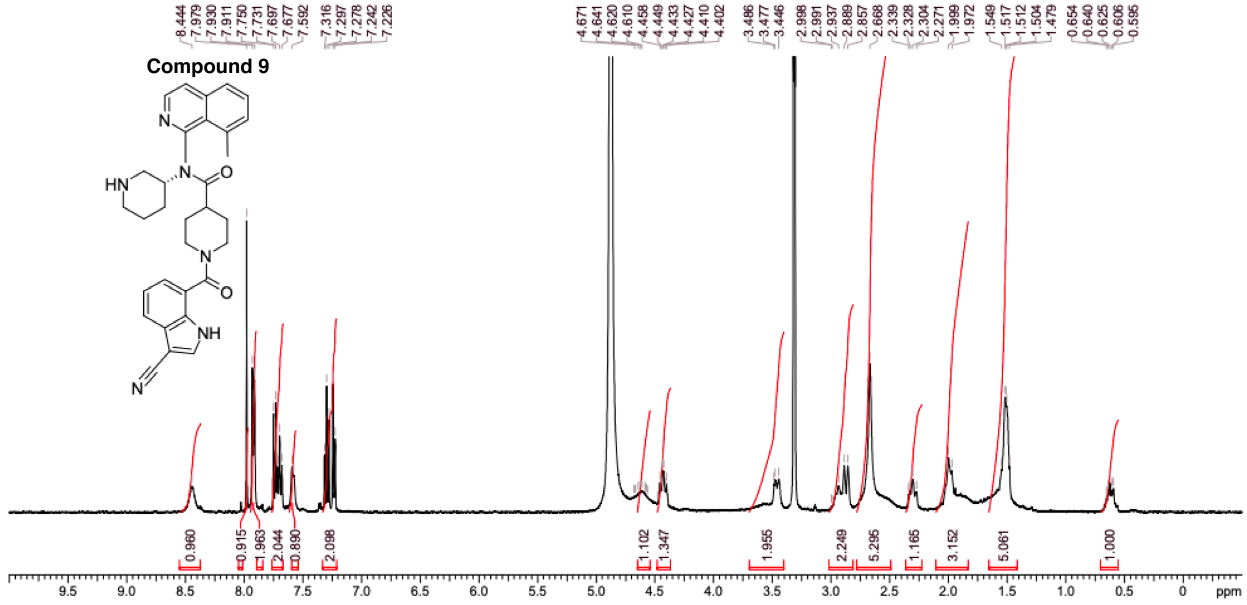

^{1}H NMR (400 MHz, CD_3OD) δ 8.44 (br s, 1H), 7.98 (s, 1H), 7.92 (br d, J=7.63 Hz, 2H), 7.67 - 7.76 (m, 2H), 7.58 (br d, J=5.88 Hz, 1H), 7.21 - 7.33 (m, 2H), 4.54 - 4.65 (m, 1H), 4.37 - 4.48 (m, 1H), 3.40 - 3.70 (m, 2H), 2.81 - 3.02 (m, 2H), 2.67 (br s, 5 H), 2.23 - 2.36 (m, 1H),

1.99 (br d, J=11.13 Hz, 3H), 1.41 - 1.66 (m, 5H), 0.55 - 0.71 (m, 1H)

Synthesis of (R)-1-(3-methyl-4-oxo-4,5,6,7-tetrahydro-1H-indole-2-carbonyl)-N-(8-methyl isoquinolin-1-yl)-N-(piperidin-3-yl)piperidine-4-carboxamide (Compound 11)

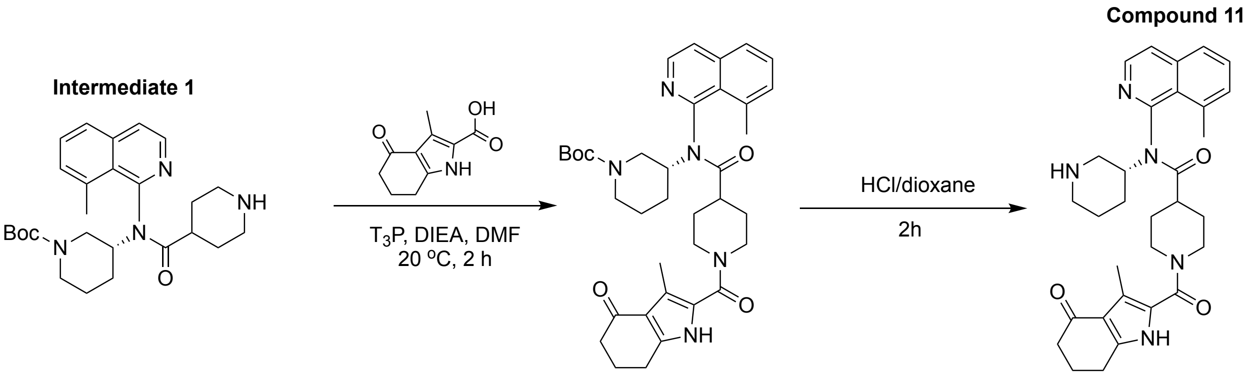

Step 1: To a solution of tert-butyl (R)-3-(N-(8-methylisoquinolin-1-yl)piperidine-4-carboxamido) piperidine-1-carboxylate (500 mg, 1.10 mmol) and 3-methyl-4-oxo-4,5,6,7-tetrahydro-1H-indole-2-carboxylic acid (226 mg, 1.22 mmol) in DMF (5 mL) were added T_3P (630 mg, 1.66 mmol) and DIEA (428 mg, 3.31 mmol, 577 µL). The mixture was stirred at 20 °C for 2 h, after which it was poured into water (20 mL). The aqueous phase was extracted with ethyl acetate (3 x 10 mL). The combined organic phases were washed with brine (2 x 10 mL), dried with anhydrous Na_2SO_4, filtered, and concentrated. The crude product was used in the next step directly without purification. Tert-butyl (R)-3-(1-(3-methyl-4-oxo-4,5,6,7-tetrahydro-1H-indole-2-carbonyl)-N-(8-methylisoquinolin-1-yl) piperidine-4-carboxamido)piperidine-1-carboxylate (0.30 g, 483 µmol) was obtained as a yellow oil. LC-MS: $[M+H^+]$ = 621.3

Step 2: To a solution of tert-butyl (R)-3-(1-(3-methyl-4-oxo-4,5,6,7-tetrahydro-1H-indole-2-carbonyl)-N-(8-methylisoquinolin-1-yl)piperidine-4-carboxamido)piperidine-1-carboxylate (0.30 g, 483 µmol) in dioxane (2 mL) was added HCl/dioxane (4 M, 3 mL). The mixture was stirred at 20 °C for 2 h, after which it was concentrated. The residue was purified by preparative HPLC (column: Welch Xtimate C18 40 x 200 mm 7 µm; mobile phase: [wa-

ter (NH_3H_2O+NH_4HCO_3)-ACN]; gradient: 20%-60% B over 25 min). Compound 11 (22.5 mg, 43 µmol) was obtained as a yellow solid. LC-MS: [$M+H^+$] = 521.3

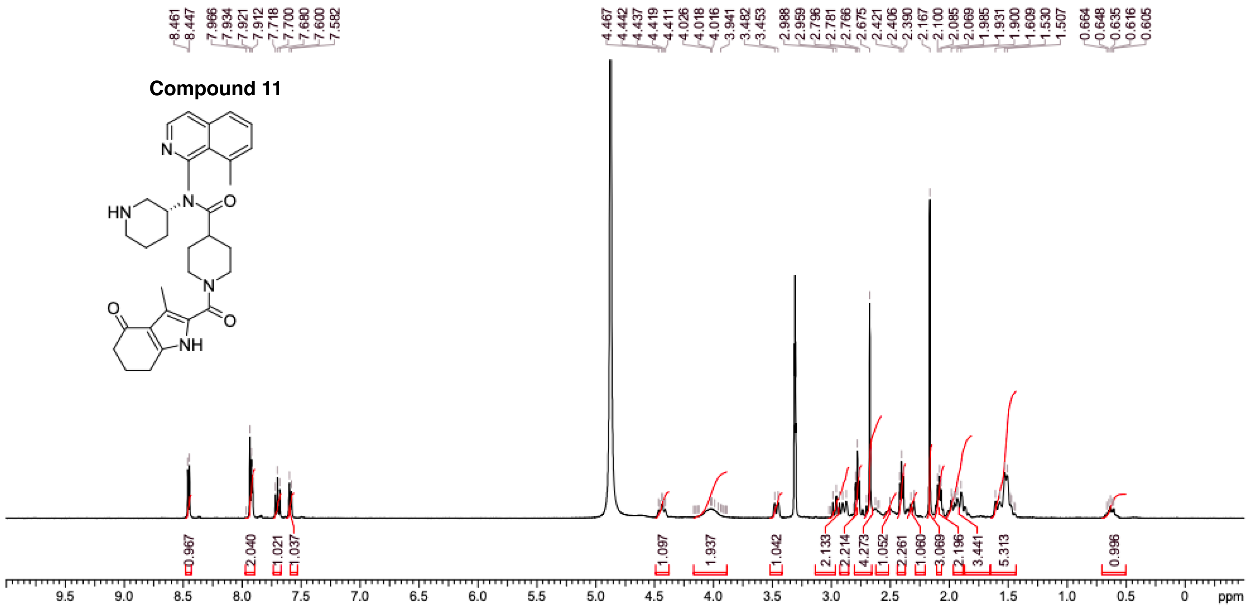

^1H NMR (400 MHz, CD_3OD) δ 8.45 (d, J=5.63 Hz, 1 H), 7.89 - 7.97 (m, 2 H), 7.70 (t, J=7.63 Hz, 1 H), 7.59 (d, J=7.00 Hz, 1 H), 4.38 - 4.49 (m, 1 H), 3.89 - 4.17 (m, 2 H), 3.47 (br d, J=11.51 Hz, 1 H), 2.85 - 3.02 (m, 2 H), 2.74 - 2.82 (m, 2 H), 2.58 - 2.72 (m, 4 H), 2.50 (br d, J=1.50 Hz, 1 H), 2.41 (t, J=6.32 Hz, 2 H), 2.31 (br d, J=10.63 Hz, 1 H), 2.17 (s, 3 H), 2.05 - 2.13 (m, 2 H), 1.81 - 2.03 (m, 3 H), 1.44 - 1.65 (m, 5 H), 0.50 - 0.71 (m, 1 H)

Synthesis of (R)-1-(3-(4-hydroxytetrahydro-2H-pyran-4-yl)propioloyl)-N-(8-methyl isoquinolin-1-yl)-N-(piperidin-3-yl)piperidine-4-carboxamide (Compound 12)

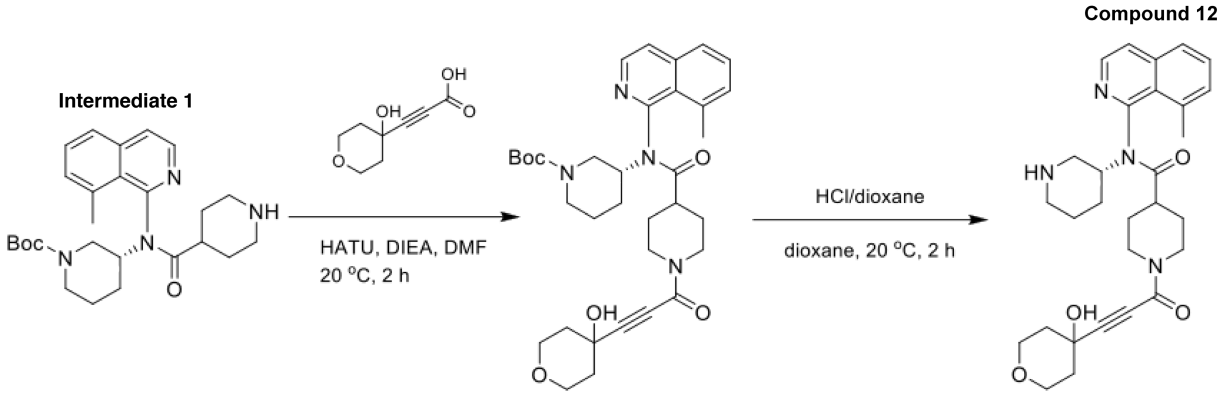

Step 1: To a solution of tert-butyl (R)-3-(N-(8-methylisoquinolin-1-yl)piperidine-4-carboxamido)piperidine-1-carboxylate (0.30 g, 663 µmol) and 3-(4-hydroxytetrahydro-2H-pyran-4-yl)propiolic acid (124 mg, 729 µmol) in DMF (5 mL) were added HATU (378 mg, 994 µmol) and DIEA (171 mg, 1.33 mmol, 231 µL). The reaction was stirred at 20 °C for 2 h, after which it was poured into water (200 mL). The aqueous phase was extracted with ethyl acetate (3 x 100 mL). The combined organic phases were washed with brine (2 x 100 mL), dried with anhydrous Na_2SO_4, filtered, and concentrated. The crude product, tert-butyl (R)-3-(1-(3-(4-hydroxytetrahydro-2H-pyran-4-yl)propioloyl)-N-(8-methylisoquinolin-1-yl)piperidine-4-carboxamido)piperidine-1-carboxylate was obtained as a yellow oil.

Step 2: To a solution of tert-butyl (R)-3-(1-(3-(4-hydroxytetrahydro-2H-pyran-4-yl)propioloyl)-N-(8-methylisoquinolin-1-yl)piperidine-4-carboxamido)piperidine-1-carboxylate (0.40 g, 661 µmol) in dioxane (4 mL) was added HCl/dioxane (4 M, 4.00 mL). The mixture was stirred at 20 °C for 2 h, after which it was concentrated. The residue was purified by preparative HPLC (column: Welch Xtimate C18 40 x 200 mm 7 µm; mobile phase: [water (NH_3H_2O+NH_4HCO_3)-ACN]; gradient: 6%-46% B over 25 min) and was further purified by preparative HPLC (column: Welch Xtimate C18 40 x 200 mm 7 µm; mobile phase:

[water (formic acid)-ACN]; gradient: 0%-32% B over 25 min. Compound 12 (10.5 mg, 21 µmol) was obtained as a yellow oil. LC-MS: $[M+H^+]$ = 505.3

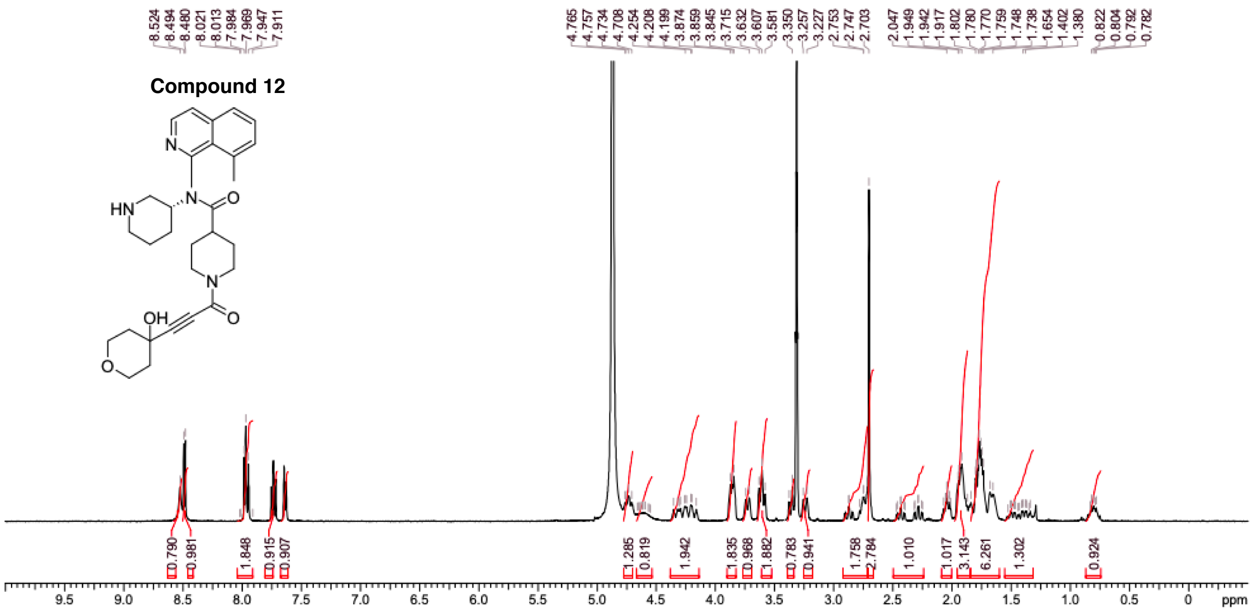

^{1}H NMR (400 MHz, CD_3OD) δ 8.49 (d, J=5.50 Hz, 1 H), 7.91 - 8.04 (m, 2 H), 7.70 - 7.77 (m, 1 H), 7.61 - 7.67 (m, 1 H), 4.70 - 4.78 (m, 1 H), 4.54 - 4.67 (m, 1 H), 4.14 - 4.38 (m, 2 H), 3.83 - 3.90 (m, 2 H), 3.73 (br d, J=11.88 Hz, 1 H), 3.56 - 3.65 (m, 2 H), 3.34 - 3.39 (m, 1 H), 3.24 (br d, J=12.26 Hz, 1 H), 2.72 - 2.92 (m, 2 H), 2.70 (s, 3 H), 2.24 - 2.50 (m, 1 H), 2.00 - 2.09 (m, 1 H), 1.87 - 1.98 (m, 3 H), 1.60 - 1.84 (m, 6 H), 1.32 - 1.56 (m, 1 H), 0.74 - 0.87 (m, 1 H)

Synthesis of (R)-N-(8-methylisoquinolin-1-yl)-N-(piperidin-3-yl)-1-(2-(pyridin-4-yl)-1H-benzo[d]imidazole-6-carbonyl)piperidine-4-carboxamide (Compound 14)

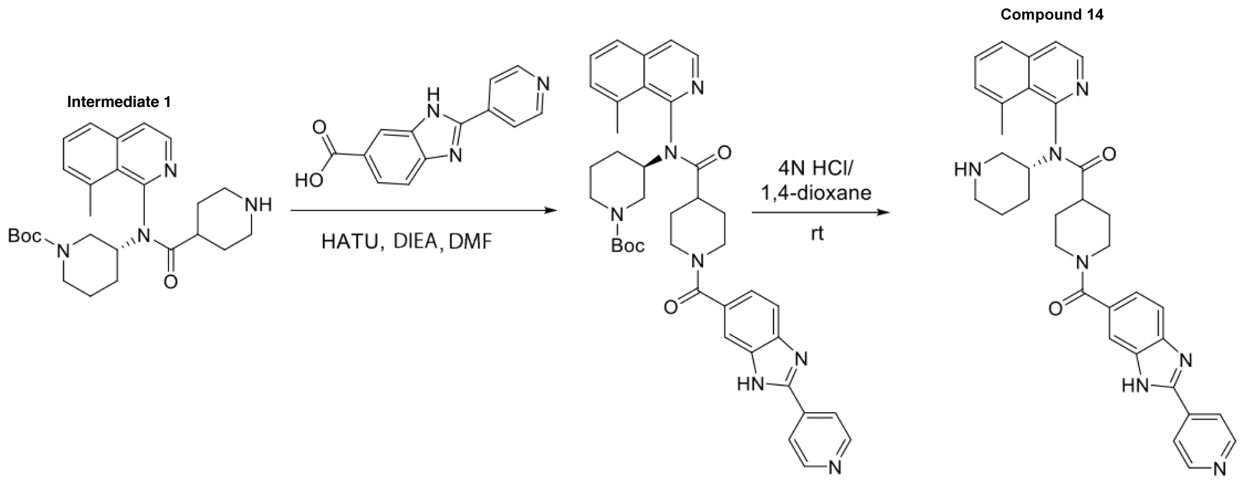

Step 1: To a solution of tert-butyl (R)-3-(N-(8-methylisoquinolin-1-yl) piperidine-4-carboxamido)piperidine-1-carboxylate (60 mg, 0.13 mmol) and 2-(pyridin-4-yl)-1H-benzo[d] imidazole-6-carboxylic acid (47 mg, 0.19 mmol) in DMF (3 mL) were added HATU (75 mg, 0.19 mmol) and DIEA (0.07 mL, 0.39 mmol) at 0 °C. The reaction was stirred at room temperature for 2 h. The reaction mixture was diluted with water (20 mL) and extracted with EtOAc (2 x 30 mL). The combined organic layers were washed with saturated NaCl, dried over Na_2SO_4, and concentrated. The obtained compound was purified by Combi-Flash, eluting with 10% MeOH in DCM, and was further purified by prep HPLC (Conditions: Luna OMEGA C-18 (250 x 21.2 x 5 µm), mobile phase A: 0.1% formic acid in water (100 %), Mobile phase B: 100 % acetonitrile, gradient: T %B: 0/40, 10/70, Flow: 15mL/min). Pure fractions were combined and dried to afford tert-butyl (R)-3-(N-(8-methylisoquinolin-1-yl)-1-(2-(pyridin-4-yl)-1H-benzo[d]imidazole-6-carbonyl)piperidine-4-carboxamido) piperidine-1-carboxylate (12 mg, 0.01 mmol) as a white solid. LC-MS: [M+H$^+$] = 674.40.

Step 2: Tert-butyl (R)-3-(N-(8-methylisoquinolin-1-yl)-1-(2-(pyridin-4-yl)-1H-benzo[d] imidazole-6-carbonyl)piperidine-4-carboxamido)piperidine-1-carboxylate (10 mg, 0.01 mmol) was dissolved in DCM (2 mL), and 4M HCl in 1,4-dioxane (2 mL) was added at 0 °C. The

reaction was stirred at room temperature for 2 h. The reaction mixture was concentrated to afford Compound 14 (7 mg, 0.01 mmol) as a light-brown solid. LC-MS: [M+H$^+$] = 574.30.

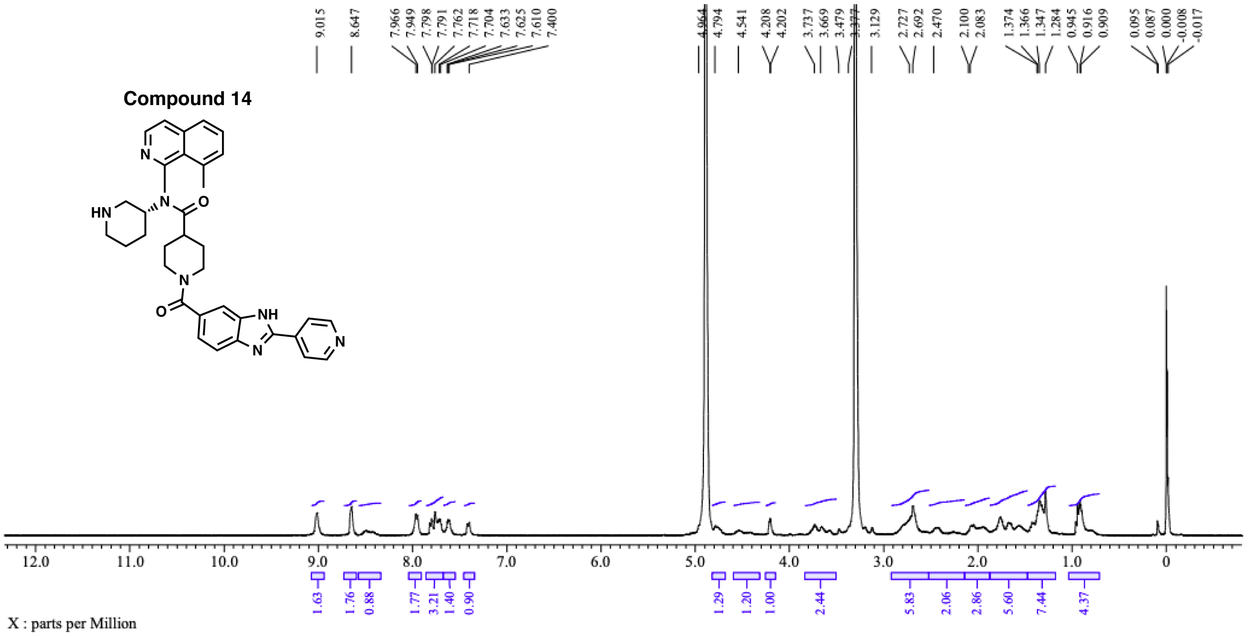

^{1}H NMR (CD_3OD, 400 MHz): δ 9.04-8.98 (m, 2H), 8.68-8.62 (m, 2H), 7.96-7.94 (m, 2H), 7.87-7.79 (m, 3H), 7.63-7.60 (m, 2H), 7.42-7.40 (m, 1H), 4.79-4.69 (m, 1H), 4.54-4.44 (m, 1H), 4.21-4.20 (m, 1H), 3.76-3.66 (m, 2H), 2.82-2.69 (m, 5H), 2.47-2.27 (m, 2H), 2.10 - 2.80 (m, 2H), 1.77 -1.58 (m, 5H), 1.38 -1.28 (m, 1H), 0.96 -0.89 (m, 1H).

Synthesis of Benzyl (S)-3-(((R)-1-(tert-butoxycarbonyl)piperidin-3-yl)(8-methylisoquinolin-1-yl)carbamoyl)piperidine-1-carboxylate (Intermediate 2)

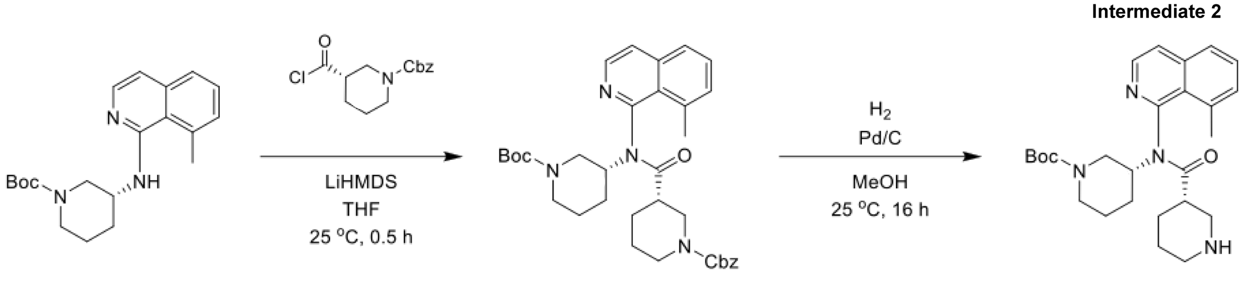

Step 1: To a solution of tert-butyl (R)-3-((8-methylisoquinolin-1-yl)amino)piperidine-1-carboxylate (4.50 g, 13.2 mmol) in THF (90 mL) was added LiHMDS (1 M, 21.1 mL) under N_2. The mixture was stirred at 25 °C for 15 min under N_2. Then, benzyl (S)-3-(chlorocarbonyl)piperidine-1-carboxylate (4.83 g, 17.1 mmol) was added to the reaction. The mixture was stirred at 25 °C for 15 min under N_2. The reaction mixture was quenched by adding saturated NH_4Cl (500 mL), and then extracted with ethyl acetate (3 x 250 mL). The combined organic layers were washed with brine (500 mL), dried over $MgSO_4$, filtered, and concentrated. The residue was purified by silica gel (ISCO®; 40 g SepaFlash® Silica Flash Column, Eluent of 0 15% THF/PE @ 100 mL/min). Benzyl (S)-3-(((R)-1-(tert-butoxycarbonyl)piperidin-3-yl)(8-methylisoquinolin-1-yl)carbamoyl)piperidine-1-carboxylate (4.70 g, 8.01 mmol) was obtained as a yellow solid. LC-MS: $[M+H^+]$ = 587.3

Step 2: To a solution of benzyl (S)-3-(((R)-1-(tert-butoxycarbonyl)piperidin-3-yl)(8-methyl isoquinolin-1-yl)carbamoyl)piperidine-1-carboxylate (4.70 g, 8.01 mmol) in MeOH (100 mL) was added Pd/C (470 mg, 442 µmol, 10% purity) under N_2. The suspension was degassed and purged with H_2. The mixture was stirred under H_2 (50 Psi) at 25 °C for 16 h. The reaction mixture was filtered through celite and concentrated. The crude product was triturated with 50 mL n-pentane at 25 °C for 180 min. Intermediate 2 (2.70 g, 5.97 mmol) was obtained as a yellow solid. LC-MS: $[M+H^+]$ = 453.8.

Synthesis of (S)-1-(3-cyano-1H-indole-7-carbonyl)-N-(8-methylisoquinolin-1-yl)-N-((R)-piperidin-3-yl)piperidine-3-carboxamide (Compound 8)

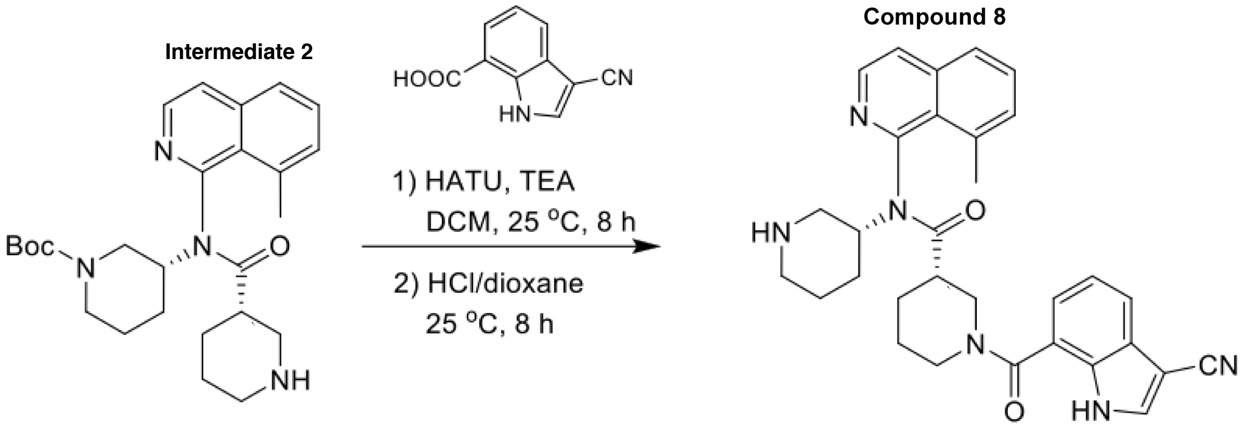

To a solution of tert-butyl (R)-3-((S)-N-(8-methylisoquinolin-1-yl)piperidine-3-carboxamido) piperidine-1-carboxylate (123 mg, 663 µmol) in DCM (9 mL) were added HATU (315 mg, 829 µmol) and TEA (101 mg, 994 µmol). The mixture was stirred at 25 °C for 30 min. Then, 3-cyano-1H-indole-7-carboxylic acid (300 mg, 663 µmol) was added to the reaction. The mixture was stirred at 25 °C for 7.5 h. After that, HCl/dioxane (2 M, 18 mL) was added to the reaction. The mixture was stirred at 25 °C for 8 h. The reaction mixture was concentrated and purified by preparative HPLC (formic acid condition: column: Welch Xtimate C18 40 x 200 mm 7 µm; mobile phase: [water (formic acid)-ACN]; gradient: 0%-38% B over 30 min). Compound 8 (115 mg, 0.19 mmol) was obtained as a white solid. LC-MS: $[M+H^+]$ = 521.3

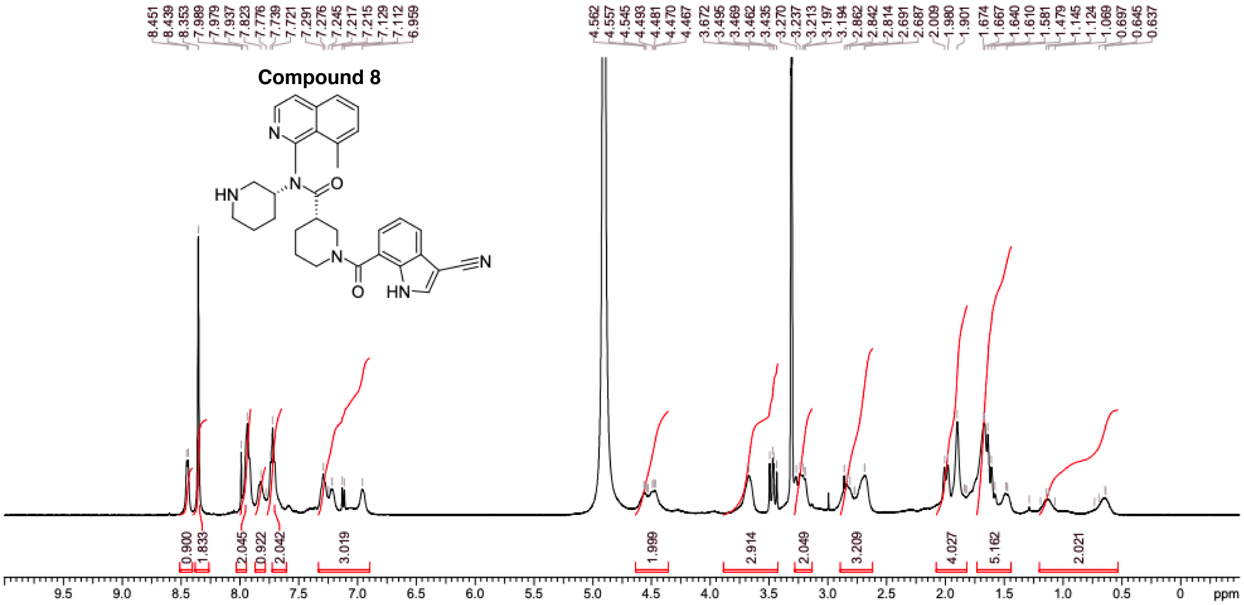

^{1}H NMR (400 MHz, CD_3OD) δ 8.45 (br d, J = 4.9 Hz, 1H), 8.35 (s, 2H), 8.00 - 7.91 (m, 2H), 7.82 (br s, 1H), 7.77 - 7.64 (m, 2H), 7.33 - 6.90 (m, 3H), 4.64 - 4.36 (m, 2H), 3.89 - 3.43 (m, 3H), 3.28 - 3.14 (m, 2H), 2.89 - 2.62 (m, 3H), 2.08 - 1.82 (m, 4H), 1.73 - 1.44 (m, 5H), 1.20 - 0.54 (m, 2H)

Synthesis of (S)-N-(8-methylisoquinolin-1-yl)-1-(4-(oxazol-5-yl)benzoyl)-N-((R)-piperidin-3-yl)piperidine-3- carboxamide (Compound 10)

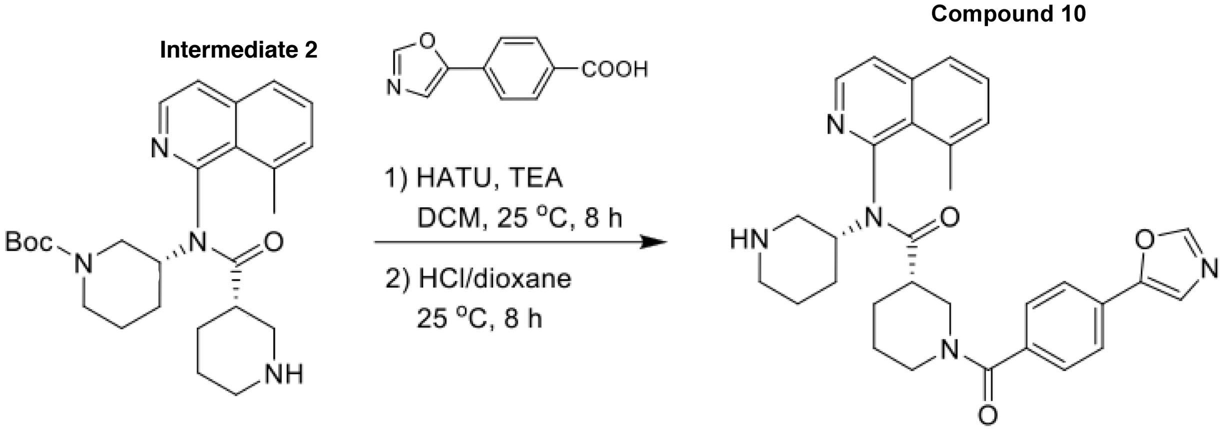

To a solution of tert-butyl (R)-3-((S)-N-(8-methylisoquinolin-1-yl)piperidine-3-carboxamido) piperidine-1-carboxylate (138 mg, 729 µmol) in DCM (9 mL) were added HATU (315 mg,

829 μmol) and TEA (101 mg, 994 μmol). The mixture was stirred at 25 °C for 30 min. Then, 4-(oxazol-5-yl)benzoic acid (300 mg, 663 μmol) was added to the reaction. The mixture was stirred at 25 °C for 7.5 h. After that, HCl/dioxane (2 M, 18 mL) was added to the reaction. The mixture was stirred at 25 °C for 8 h. The reaction mixture was concentrated to give a residue that was purified by preparative HPLC (formic acid condition: column: Welch Xtimate C18 40 x 200 mm 7 μm; mobile phase: [water (formic acid)-ACN]; gradient: 0%-36% B over 30 min). Compound 10 (155 mg, 0.27 mmol) was obtained as a light-yellow solid. LC-MS: [M+H$^+$] = 524.3

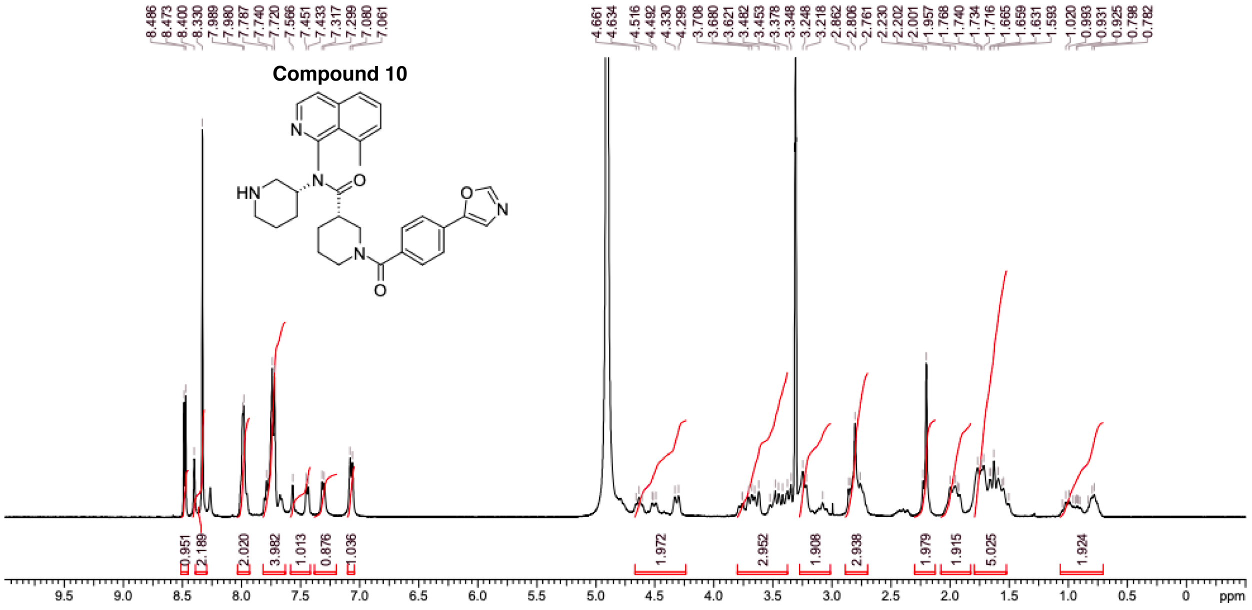

^{1}H NMR (400 MHz, CD_3OD) δ 8.48 (d, J = 5.5 Hz, 1H), 8.41 - 8.31 (m, 2H), 7.98 (br d, J = 3.5 Hz, 2H), 7.82 - 7.63 (m, 4H), 7.59 - 7.42 (m, 1H), 7.31 (br d, J = 7.0 Hz, 1H), 7.07 (br d, J = 7.6 Hz, 1H), 4.67 - 4.24 (m, 2H), 3.80 - 3.38 (m, 3H), 3.27 - 3.01 (m, 2H), 2.89 - 2.70 (m, 3H), 2.30 - 2.13 (m, 2H), 2.08 - 1.83 (m, 2H), 1.79 - 1.52 (m, 5H), 1.07 - 0.71 (m, 2H)

Synthesis of (S)-N-(8-methylisoquinolin-1-yl)-N-((R)-piperidin-3-yl)-1-(6-(pyrrolidin-1-yl)nicotinoyl)piperidine-3-carboxamide (Compound 13)

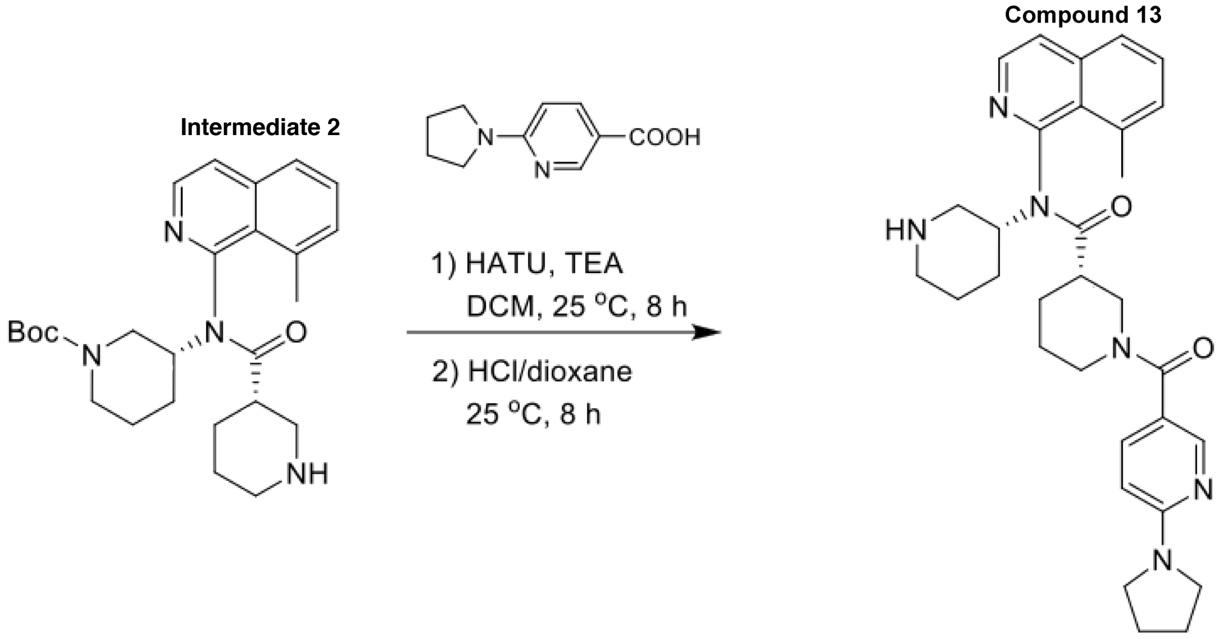

To a solution of tert-butyl (R)-3-((S)-N-(8-methylisoquinolin-1-yl)piperidine-3-carboxamido) piperidine-1-carboxylate (140 mg, 729 µmol) in DCM (9 mL) were added HATU (315 mg, 829 µmol) and TEA (101 mg, 994 µmol). The mixture was stirred at 25 °C for 30 min. Then, 6-(pyrrolidin-1-yl)nicotinic acid (300 mg, 663 µmol) was added to the reaction. The mixture was stirred at 25 °C for 7.5 h. After that, HCl/dioxane (2 M, 18 mL) was added to the reaction. The mixture was stirred at 25 °C for 8 h. The reaction mixture was concentrated to give a residue that was purified by prep-HPLC (formic acid condition: column: Welch Xtimate C18 40 x 200 mm 7 µm; mobile phase: [water (formic acid)-ACN]; gradient: 0%-28% B over 20 min). Compound 12 (95.0 mg, 0.17 mmol) was obtained as a light-yellow solid. LC-MS: $[M+H^+]$ = 527.4

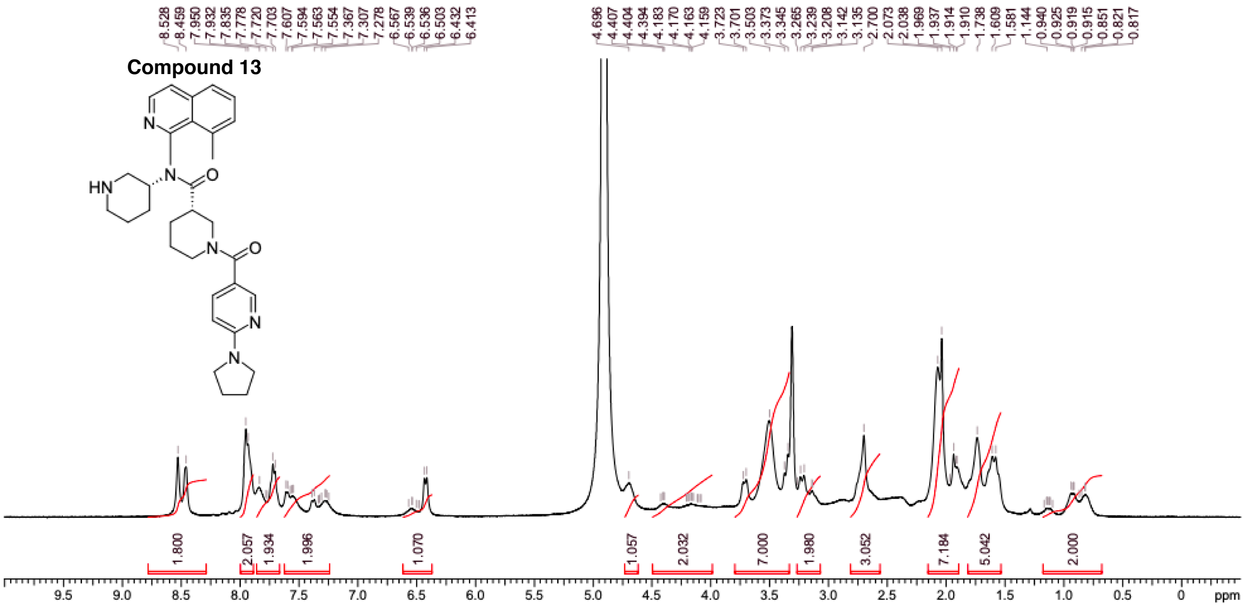

^{1}H NMR (400 MHz, CD_3OD) δ 8.78 - 8.29 (m, 2H), 7.94 (br d, J = 7.1 Hz, 2H), 7.86 - 7.66 (m, 2H), 7.62 - 7.24 (m, 2H), 6.61 - 6.37 (m, 1H), 4.70 (br s, 1H), 4.49 - 3.99 (m, 2H), 3.79 - 3.33 (m, 7H), 3.27 - 3.07 (m, 2H), 2.70 (br s, 3H), 2.16 - 1.89 (m, 7H), 1.82 - 1.54 (m, 5H), 1.18 - 0.68 (m, 2H)

Supporting Figures

Supporting Figure S1

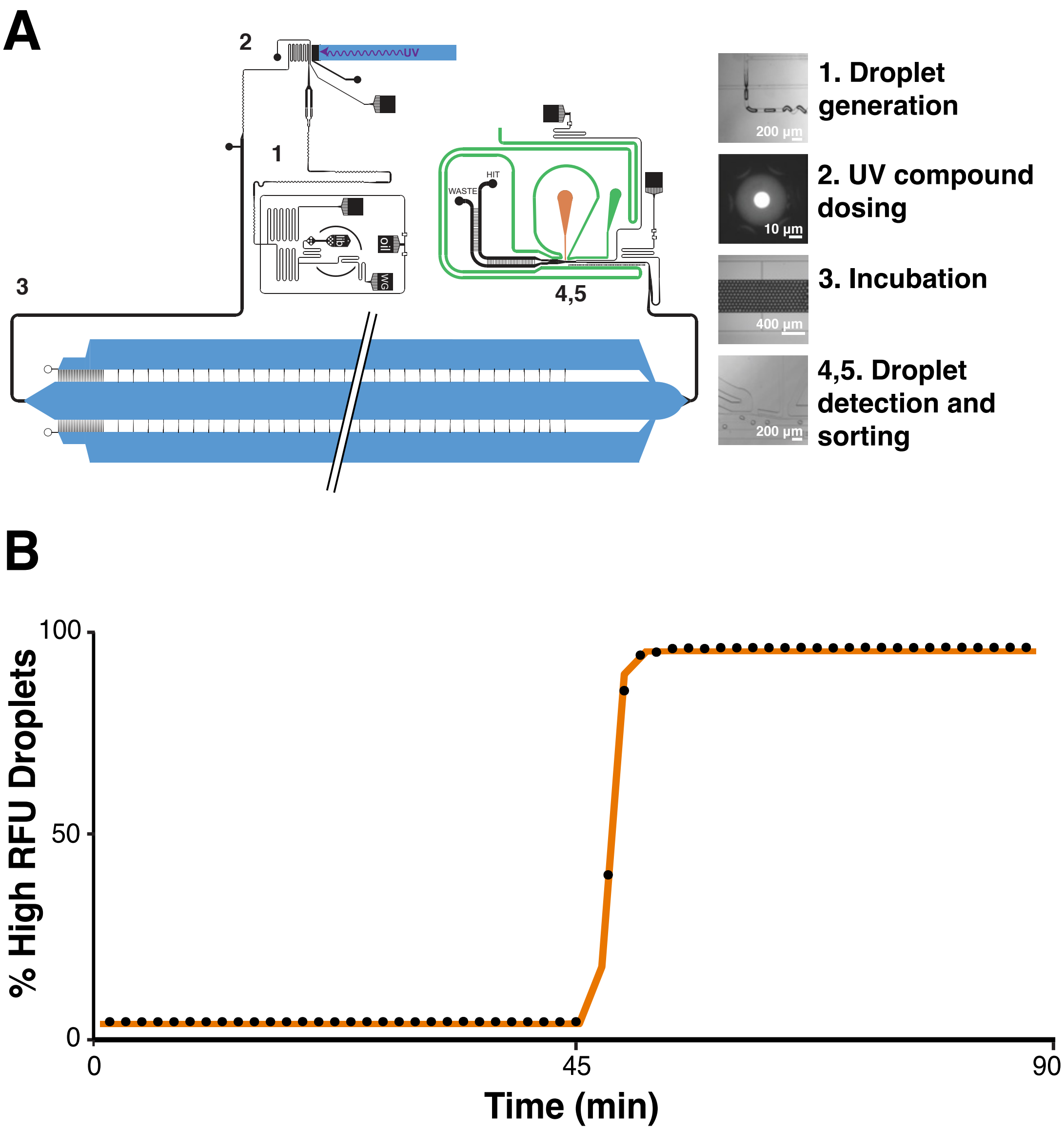

Figure S1. Microfluidic screening device design and characterization. (A) WG IVTT enters the circuit (WG) along with library beads (lib). Aqueous stream meets the flow focusing oil (OIL) junction to form droplets **(1)**. Droplets are irradiated with UV to release compound from beads **(2)**. Droplets then enter the incubator **(3)**. After emerging from the incubator, droplets are interrogated with laser-induced fluorescence and electrokinetically sorted based on their signal **(4,5)**. (B) Incubation time and dispersion were determined by alternating dye flow rates, calculating the time, and fitting a cumulative distribution function to the transition between high- and low-dye concentration droplets.

Supporting Figure S2

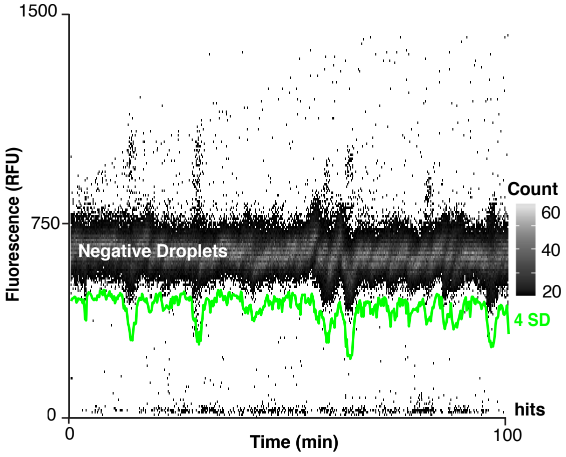

Figure S2. Hygromycin B bead activity in droplets. Transient histogram visualization of the PCSK9[1:33]-GFP droplets shows a population of negative droplets, the PC-HygB control 'hit' droplets, and the dynamic sorting threshold that is set 4σ below the mean of the negative droplet population. Droplet data were binned by time and fluorescence (30 s, 7 RFU).

Supporting Figure S3

Compound ID	Structure	Observed m/z (expected m/z, * truncate m/z)[1]
Linker	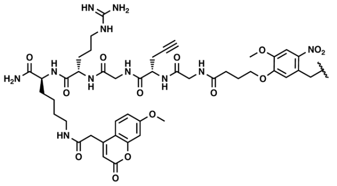	+ 974
QC-1	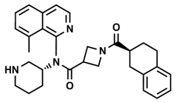	1458 (1457 $[M+H]^+$)
QC-2	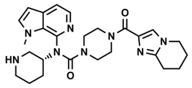	1466 (1466 $[M+H]^+$)
QC-3	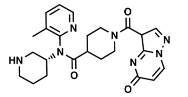	1439 (1439 $[M+H]^+$)
QC-4	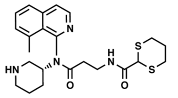	1434 (1434 $[M+H]^+$)
QC-5	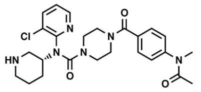	1474 (1474 $[M+H]^+$)
QC-6	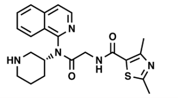	1260 (1399 $[M+H]^+$, *1260 $[M+H]^+$)
QC-7	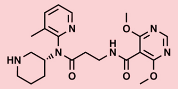	1274 (1403 $[M+H]^+$)
QC-8	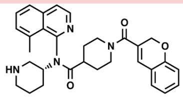	1487 (1486 $[M+H]^+$)
QC-9	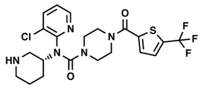	1477 (1477 $[M+H]^+$)
QC-10	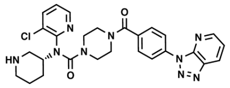	1521 (1521 $[M+H]^+$)

[1]m/z values displayed include mass of the linker, which remains covalently bound to the library member until photocleavage.

Figure S3. DEL quality control results 40 200-µm resins (1-10 shown here) were sequenced and the predicted m/z of the decoded structure was compared to the observed m/z by MALDI-TOF MS. The expected m/z was not observed in two samples (QC-7 shown here). The expected product of the full product or predicted truncate (*) was observed for the other samples.

Supporting Figure S4

Compound ID	Structure	Observed m/z (expected m/z, * truncate m/z)[1]
Linker	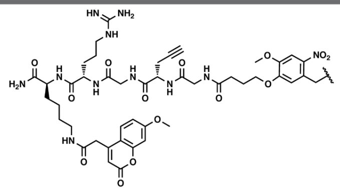	+ 974
QC-11	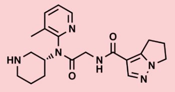	1476 (1358 [M+H]$^+$)
QC-12	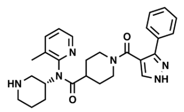	1448 (1448 [M+H]$^+$)
QC-13	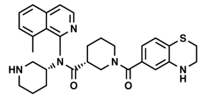	1505 (1505 [M+H]$^+$)
QC-14	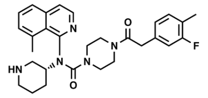	1479 (1479 [M+H]$^+$)
QC-15	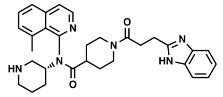	1501 (1500 [M+H]$^+$)
QC-16	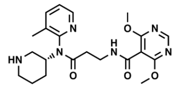	1404 (1403 [M+H]$^+$)
QC-17	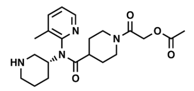	1378 (1378 [M+H]$^+$)
QC-18	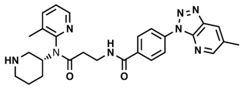	1474 (1473 [M+H]$^+$)
QC-19	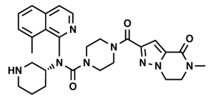	1506 (1506 [M+H]$^+$)
QC-20	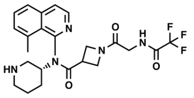	1453 (1452 [M+H]$^+$)

[1]m/z values displayed include mass of the linker, which remains covalently bound to the library member until photocleavage.

Figure S4. DEL quality control results 40 200-µm resins (11-20 shown here) were sequenced and the predicted m/z of the decoded structure was compared to the observed m/z by MALDI-TOF MS. The expected m/z was not observed in two samples (QC-11 shown here). The expected product of the full product or predicted truncate (*) was observed for the other samples.

Supporting Figure S5

Compound ID	Structure	Observed m/z (expected m/z, * truncate m/z)[1]
Linker	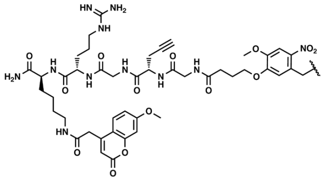	+ 974
QC-21	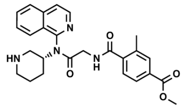	1260 (1436 $[M+H]^{+}$), 1260 $[M+H]^{+}$)
QC-22	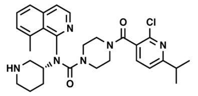	1510 (1510 $[M+H]^{+}$)
QC-23	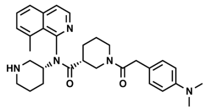	1489 (1489 $[M+H]^{+}$)
QC-24	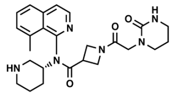	1440 (1439 $[M+H]^{+}$)
QC-25	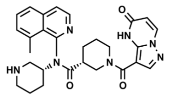	1489 (1489 $[M+H]^{+}$)
QC-26	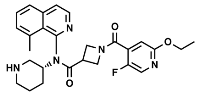	1467 (1466 $[M+H]^{+}$)
QC-27	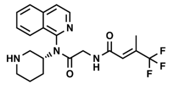	1260 (1396 $[M+H]^{+}$, *1260 $[M+H]^{+}$)
QC-28	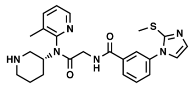	1224 (1440 $[M+H]^{+}$, *1224 $[M+H]^{+}$)
QC-29	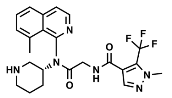	1274 (1450 $[M+H]^{+}$, *1274 $[M+H]^{+}$)
QC-30	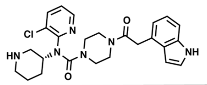	1459 (1456 $[M+H]^{+}$)

[1]m/z values displayed include mass of the linker, which remains covalently bound to the library member until photocleavage.

Figure S5. DEL quality control results 40 200-µm resins (21-30 shown here) were sequenced and the predicted m/z of the decoded structure was compared to the observed m/z by MALDI-TOF MS. The expected m/z was not observed in two samples. The expected product of the full product or predicted truncate (*) was observed for the other samples.

Supporting Figure S6

Compound ID	Structure	Observed m/z (expected m/z, * truncate m/z)[1]
Linker	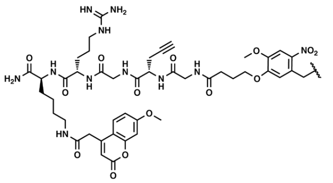	+ 974
QC-31	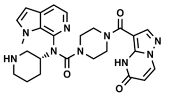	1479 (1479 $[M+H]^+$)
QC-32	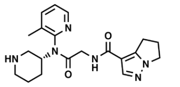	1224 (1358 $[M+H]^+$, *1224 $[M+H]^+$)
QC-33	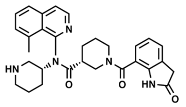	1487 (1487 $[M+H]^+$)
QC-34	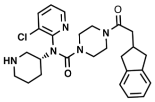	1457 (1457$[M+H]^+$)
QC-35	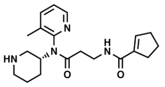	1332 (1331 $[M+H]^+$)
QC-36	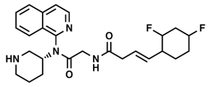	1260 (1440 $[M+H]^+$, *1260 $[M+H]^+$)
QC-37	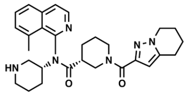	1476 (1476 $[M+H]^+$)
QC-38	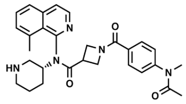	1475 (1474 $[M+H]^+$)
QC-39	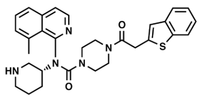	1503 (1503 $[M+H]^+$)
QC-40	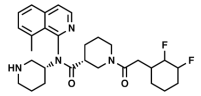	1482 (1482 $[M+H]^+$)

[1]m/z values displayed include mass of the linker, which remains covalently bound to the library member until photocleavage.

Figure S6. DEL quality control results 40 200-µm resins (31-40 shown here) were sequenced and the predicted m/z of the decoded structure was compared to the observed m/z by MALDI-TOF MS. The expected m/z was not observed in two samples. The expected product of the full product or predicted truncate (*) was observed for the other samples.

889 # Supporting Figure S7

	Theoretical	1500-Bead Samples			
k class	# beads	1	2	3	FDR (%)
0	3983	4355	4093	4153	NA
1	1195	847	1026	988	85
2	179	106	166	149	12
3	18	10	26	25	1.8
4	1	2	7	4	0.37
5	0.1	2	2	1	0.15

Figure S7. False discovery rate determination. Random samples of the DEL (1500 beads, 3 replicates) were sequenced, deconvoluted, and aggregated by *k* class (gold). The poisson distribution of *k* classes in a 1500-bead random sample is also shown (theoretical, blue). False discovery rate (FDR) was calculated as the average observed number of compounds at each *k* class in the random sample divided by the total number of compounds observed as hits at each *k* class.

Supporting Figure S8

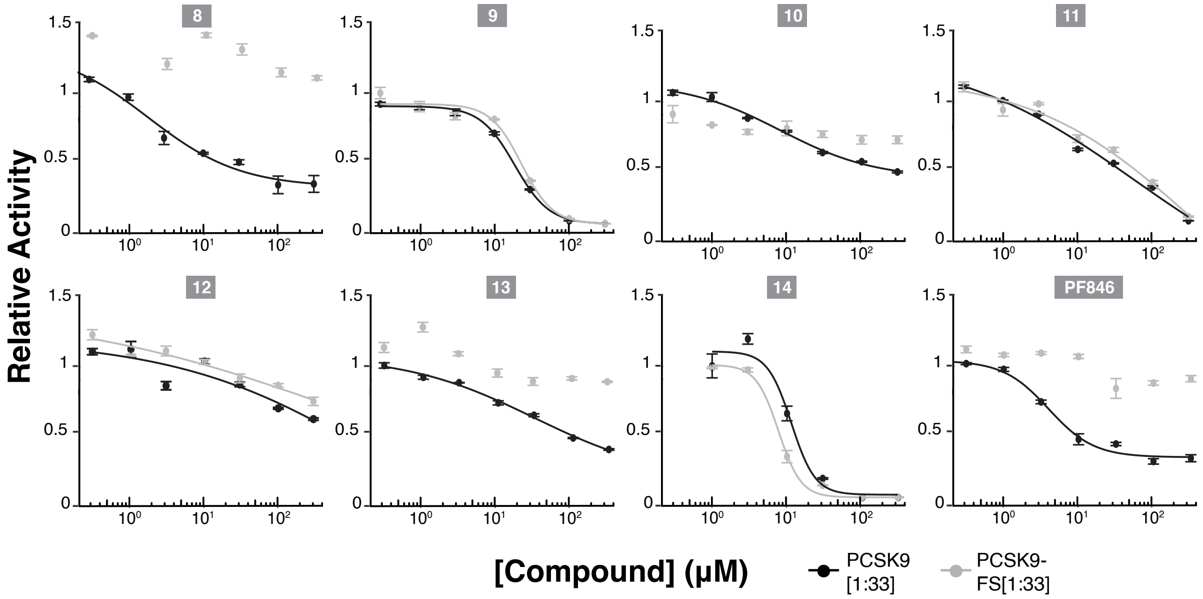

Figure S8. Screening hit IC_{50} analysis. Screening hits and PF846 were analyzed in dose-response WG IVTT activity assays. PCSK9[1:33]-GFP (black) and PCSK9-FS[1:33]-GFP (gray) templates were studied. Error bars indicate SDOM (n=3).

891 **Supporting Figure S9**

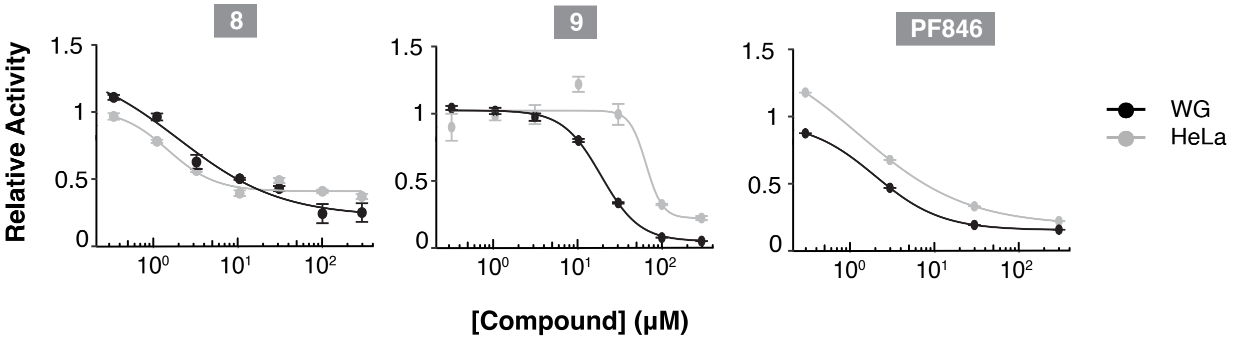

Figure S9. Compounds 8 and 9 validation in HeLa IVTT. Screening hits **8** and **9** were analyzed in dose-response activity assays using WG lysate (black) or HeLa lysate (gray). Error bars indicate SDOM (n=3).

Supporting Figure S10

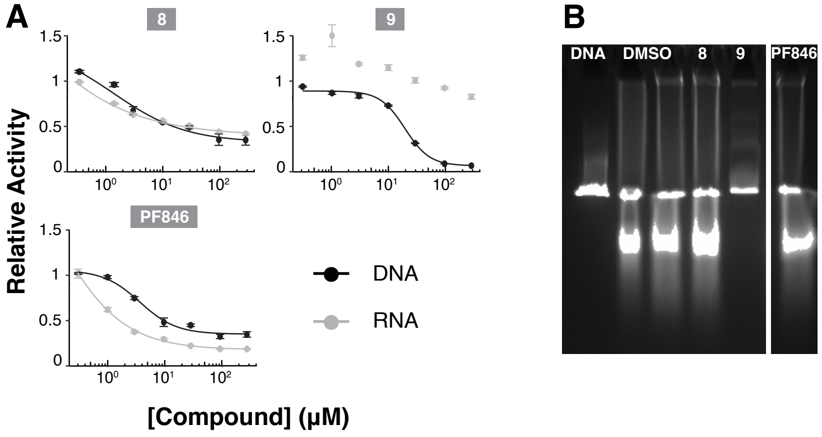

Figure S10. Activity of compounds 8 and 9 on transcription. (A) Screening hits **8** and **9** were analyzed in dose-response IVTT activity assays of PCSK9[1:33]-GFP initiated with DNA (black) and RNA (gray) template. (B) Products of PCSK9[1:33]-GFP T7 transcription reactions incubated with DMSO, screening hits **8** and **9**, or PF846 were visualized on a 1.5% agarose gel

Supporting Figure S11

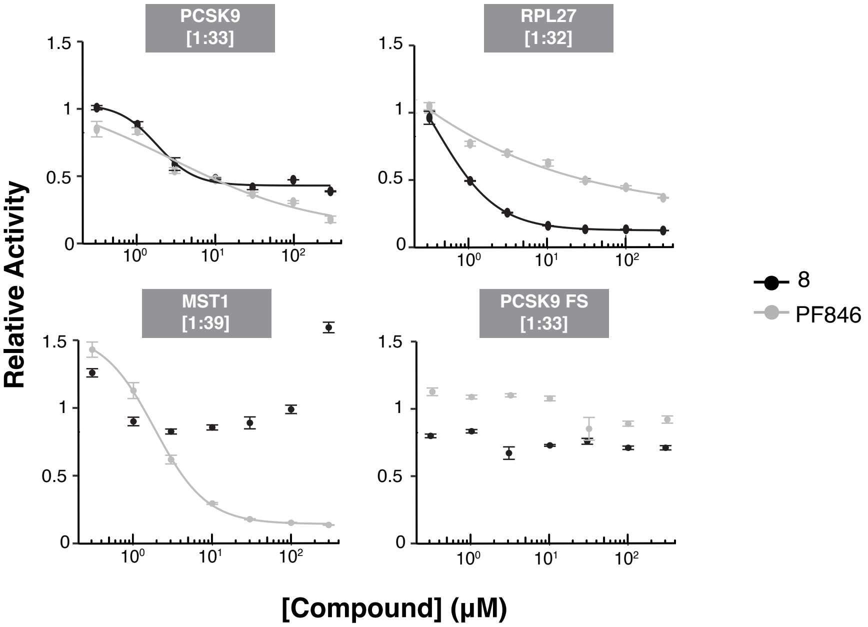

Figure S11. Off-target profiling of compound 8. Compound **8** (black) and PF846 (gray) were analyzed in dose-response WG IVTT activity assays. Templates PCSK9[1:33]-GFP, RPL27[1:32]-GFP, MST1[1:39]-GFP, and PCSK9-FS[1:33]-GFP were studied.

Supporting Figure S12

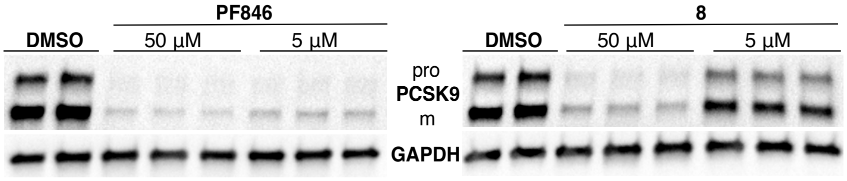

Figure S12. Acitivity of PF846 and compound 8 in HepG2 cells. PCSK9 expression in HepG2 cells treated 16 h with PF846 or screening hit **8** (5 or 50 µM) or vehicle control (DMSO) was analyzed in triplicate by Western blot.

Supporting Tables

Supporting Table S1

Table S1. High-Resolution Mass Spectra

Compound	Theoretical Mass	Observed Mass
1	864.3587 ([M+Na$^+$])	864.3586 ([M+Na$^+$])
2	842.3759	842.3759
3	832.3664	832.3656
8	521.2660	521.2665
9	521.2660	521.2665
10	524.2656	524.2662
11	528.2970	528.2975
12	505.2810	505.2815
13	527.3129	527.3135
14	574.2925	574.2930

(M+H$^+$ unless otherwise noted)

Supporting Table S2

Table S2. Vector

**Component**	**Sequence**
*Sgf*I site	GCGATCGC
*Pme*I site	GTTTAAAC
*Nde*I site	CATATG
*Xho*I site	CTCGAG
pF3A Kozak sequence	ACCACCATG
PT7CFE Kozak sequence	GATGATAATATG
Linker	GGGCCCTCCGGACTCAGATCACGAGCT
GFP	GAGCTTTTCACTGGCGTTGTTCCCATCCTGGTCGAGCTGGACGGCGACGTAAACGGCCACAAGTTCAGCGTGCGCGGCGAGGGCG<br>AGGGCGATGCCACCAACGGCAAGCTGACCCTGAAGTTCATCTGCACCACCGGCAAGCTGCCCGTGCCCTGGCCCACCCTCGTGAC<br>CACCCTGACCTACGGCGTGCAGTGCTTCAGCCGCTACCCCGACCACATGAAGCAGCACGACTTCTTCAAGTCCGCCATGCCCGAA<br>GGCTACGTCCAGGAGCGCACCATCTCCTTCAAGGACGACGGCACCTACAAGACCCGCGCCGAGGTGAAGTTCGAGGGCGACACC<br>CTGGTGAACCGCATCGAGCTGAAGGGCATCGACTTCAAGGAGGACGGCAACATCCTGGGGCACAAGCTGGAGTACAACTTCAAC<br>AGCCACAACGTCTATATCACGGCCGACAAGCAGAAGAACGGCATCAAGGCGAACTTCAAGATCCGCCACAACGTCGAGGACGG<br>CAGCGTGCAGCTCGCCGACCACTACCAGCAGAACACCCCCATCGGCGACGGCCCCGTGCTGCTGCCCGACAACCACTACCTGAG<br>CACCCAGTCCAAGCTGAGCAAAGACCCCAACGAGAAGCGCGATCACATGGTCCTGCTGGAGTTCGTGACCGCCGCCGGGATCTA

Supporting Table S3

Table S3. Target Genes

Gene	Sequence
PCSK9[1:33]	ATGGGCACCGTCAGCTCCAGGCGGTCCTGGTGGCCGCTGCCACTGCTGCTGCTGCTGCTGCTGCTCCTGGGTCCCGCGGGCGCCCGTGCGCAGGAGGAC
PCSK9-FS[1:33]	ATGAGACACCGTCAGCTCCAAGCAGTCCTCGTTGCAGCTGCCACTGCAGCAGCTGCAGCTGCAGCTCCTGGCTCCCGCGGACGCCCGTGCGCAGGAGGA
RPL27[1:32]	ATGGGCAAGTTCATGAAACCTGGGAAGGTGGTGCTTGTCCTGGCTGGACGCTACTCCGGACGCAAAGCTGTCATCGTGAAGAACATTGATGATGGC
MST1[1:39]	ATGGGCCTGTGGTGGGTGACCGTGCAGCCACCAGCCAGGAGGATGGGCTGGCTGCCACTCCTGCTCCTGCTCACCCAGTGCCTGGGCGTGCCAGGCCAGAGGAGCCCACTGAACGACTTC
USO1 [259:300]	ATGCGTATGAAACCTTGGTTTGAAGTTGGAGATGAAAATTCTGGCTGGTCTGCACAGAAAGTGACCAATCTACATCTAATGCTACAGCTTGTTCGTGTATTGGTATCTCCCACCAACCCTCCTGGTGCT
KRAS^{G12D}[1:189]	ATGACTGAATATAAACTTGTGGTAGTTGGAGCTGATGGCGTAGGCAAGAGTGCCTTGACGATACAGCTAATTCAGAATCATTTTGTGGACGAATATGATCCAACAATAGAGGATTCCTACAGGAAGCAAGTAGTAATTGATGGAGAAACCTGTCTCTTGGATATTCTCGACACAGCAGGTCAAGAGGAGTACAGTGCAATGAGGGACCAGTACATGAGGACTGGGGAGGGCTTTCTTTGTGTATTTGCCATAAATAATACTAAATCATTTGAAGATATTCACCATTATAGAGAACAAATTAAAAGAGTTAAGGACTCTGAAGATGTACCTATGGTCCTAGTAGGAAATAAATGTGATTTGCCTTCTAGAACAGTAGACACAAAACAGGCTCAGGACTTAGCAAGAAGTTATGGAATTCCTTTTATTGAAACATCAGCAAAGACAAGACAGAGAGTGGAGGATGCTTTTTATACATTGGTGAGAGAGATCCGACAATACAGATTGAAAAAAATCAGCAAAGAAGAAAAGACTCCTGGCTGTGTGAAAATTAAAAAATGCATTATAATG

References

(S1) MacConnell, A. B.; McEnaney, P. J.; Cavett, V. J.; Paegel, B. M. DNA-Encoded Solid-Phase Synthesis: Encoding Language Design and Complex Oligomer Library Synthesis. *ACS Combinatorial Science* **2015**, *17*, 518–534, DOI: 10.1021/acscombsci.5b00106.

(S2) Mendes, K. R.; Malone, M. L.; Ndungu, J. M.; Suponitsky-Kroyter, I.; Cavett, V. J.; McEnaney, P. J.; MacConnell, A. B.; Doran, T. M.; Ronacher, K.; Stanley, K.; Utset, O.; Walzl, G.; Paegel, B. M.; Kodadek, T. High-throughput Identification of DNA-Encoded IgG Ligands that Distinguish Active and Latent Mycobacterium tuberculosis Infections. *ACS Chemical Biology* **2017**, *12*, 234–243, DOI: 10.1021/acschembio.6b00855.

(S3) MacConnell, A. B.; Price, A. K.; Paegel, B. M. An Integrated Microfluidic Processor for DNA-Encoded Combinatorial Library Functional Screening. *ACS Combinatorial Science* **2017**, *19*, 181–192, DOI: 10.1021/acscombsci.6b00192.

(S4) Duffy, D. C.; McDonald, J. C.; Schueller, O. J. A.; Whitesides, G. M. Rapid Prototyping of Microfluidic Systems in Poly(dimethylsiloxane). *Analytical Chemistry* **1998**, *70*, 4974–4984, DOI: 10.1021/ac980656z.

(S5) Price, A. K.; MacConnell, A. B.; Paegel, B. M. hνSABR: Photochemical Dose–Response Bead Screening in Droplets. *Analytical Chemistry* **2016**, *88*, 2904–2911, DOI: 10.1021/acs.analchem.5b04811.

(S6) Cochrane, W. G.; Hackler, A. L.; Cavett, V. J.; Price, A. K.; Paegel, B. M. Integrated, Continuous Emulsion Creamer. *Analytical Chemistry* **2017**, *89*, 13227–13234, DOI: 10.1021/acs.analchem.7b03070.

(S7) Cochrane, W. G.; Malone, M. L.; Dang, V. Q.; Cavett, V.; Satz, A. L.; Paegel, B. M.

Activity-Based DNA-Encoded Library Screening. *ACS Combinatorial Science* **2019**, *21*, 425–435, DOI: 10.1021/acscombsci.9b00037.

(S8) MacConnell, A. B.; Paegel, B. M. Poisson Statistics of Combinatorial Library Sampling Predict False Discovery Rates of Screening. *ACS Combinatorial Science* **2017**, *19*, 524–532, DOI: 10.1021/acscombsci.7b00061.
